# *Gossypium hirsutum* Salt Tolerance Is Enhanced by Overexpression of *G. arboreum* JAZ1

**DOI:** 10.3389/fbioe.2020.00157

**Published:** 2020-03-10

**Authors:** Ge Zhao, Yun Song, Qianhua Wang, Dongxia Yao, Dongliang Li, Wenqiang Qin, Xiaoyang Ge, Zuoren Yang, Wenying Xu, Zhen Su, Xueyan Zhang, Fuguang Li, Jiahe Wu

**Affiliations:** ^1^Zhengzhou Research Base, State Key Laboratory of Cotton Biology, Zhengzhou University, Zhengzhou, China; ^2^State Key Laboratory of Cotton Biology, Institute of Cotton Research, Chinese Academy of Agricultural Sciences, Anyang, China; ^3^State Key Laboratory of Plant Physiology and Biochemistry, College of Biological Sciences, China Agricultural University, Beijing, China; ^4^Key Laboratory for Ecology of Tropical Islands, Ministry of Education, College of Life Sciences, Hainan Normal University, Haikou, China; ^5^State Key Laboratory of Plant Genomics, Institute of Microbiology Research, Chinese Academy of Sciences, Beijing, China

**Keywords:** *Gossypium arboreum*, *G. hirsutum*, *GaJAZ1*, salt tolerance, root architecture, transcriptome, JA signaling pathway

## Abstract

*Gossypium arboreum* possesses many favorable traits including robust defense against biotic and abiotic stress although it has been withdrawn from the market because of lower yield and fiber quality compared to *G. hirsutum* (upland cotton). It is therefore important to explore and utilize the beneficial genes of *G. arboretum* for *G. hirsutum* cultivar breeding. Here, the function of *G. arboreum JAZ1* in tolerance to salt stress was determined through loss-of-function analysis. GaJAZ1can interact with GaMYC2 to repress expression of downstream genes whose promoters contain a G-box *cis* element, affecting plant tolerance to salinity stress. The experimental data from NaCl treatments and a 2 year continuous field trial with natural saline-alkaline soil showed that the ectopically overexpressed *GaJAZ1* significantly increased salt tolerance in upland cotton compared to the wild type, showing higher growth vigor with taller plants, increased fresh weight, and more bolls, which is due to reprogrammed expression of tolerance-related genes and promotion of root development. High-throughput RNA sequencing of *GaJAZ1* transgenic and wild-type plants showed many differentially expressed genes involved in JA signaling and biosynthesis, salt stress-related genes, and hormone-related genes, suggesting that overexpressing *GaJAZ1* can reprogram the expression of defense-related genes in *G. hirsutum* plants to increase tolerance to salt stress. The research provides a foundation to explore and utilize favorable genes from *Gossypium* species for upland cotton cultivar breeding.

## Introduction

More than 800 million hectares of land worldwide, or about 6% of the world’s total land area, are salt-affected (Munns, 2005). Moreover, the amount of arable land adversely affected by salinization is increasing and this problem has been forecasted to have a devastating effect on global agriculture as it could potentially result in the loss of up to 50% of arable land by 2050^1^. Cotton, an economic crop that is important to many national economies, is relatively salt-tolerant (Rocha-Munive et al., 2018). However, it will still be unable to meet the challenge of increasing salinization; therefore, breeding new cotton varieties with greater salinity tolerance is a high priority in plant biotechnology programs (Shao et al., 2005).

When confronted with external biotic or abiotic stimuli, plants respond by producing jasmonic acid (JA). The bioactive JA form is the amino acid conjugate JA-Ile, which is perceived by the F-box protein coronatine insensitive 1 (COI1). Subsequently, the Skp–Cullin–F-box protein complex containing COI1 (SCF^COI1^) facilitates the degradation of the JAZ (jasmonate ZIM domain) transcriptional repressors via the ubiquitin-26S proteasome pathway. JAZ proteins are degraded by the 26S proteasome and release TOPLESS (TPL) and Novel Interactor of JAZ (NINJA), relieving the downstream inhibitory effect on bHLHzip transcription factor 2 (MYC2), leading to the release of transcription factors (TFs) and consequently to the activation of its target genes by directly targeting their promoters (Dombrecht et al., 2007; Cheng et al., 2011; Fernandez-Calvo et al., 2011), which regulate gene expression through specific binding to *cis*-acting elements in the promoters of target genes (De Geyter et al., 2012). The closely related bHLH factors– MYC2, MYC3, MYC4, and MYC5 – are among the known direct targets of JAZ repressors (Fernandez-Calvo et al., 2011). Some MYCs interact with MYB proteins to regulate defense by binding to a G-box motif found in the promoter of glucosinolate biosynthesis genes (Schweizer et al., 2013). Another subgroup of bHLH factors, including jasmonate associated MYC2-LIKE (JAM), are negative regulators of the JA pathways, allowing fine regulation of the expression of defense genes (Sasaki-Sekimoto et al., 2013; Song et al., 2013).

A previous study demonstrated that application of exogenous JA can promote the re-initiation of growth after the onset of salinity stress by priming JAZ transcription (Ismail et al., 2012). JA accumulation and signaling is a necessary step for escaping salinity-induced cell death and in activating salinity adaptation (Zhu et al., 2012). Multiple different jasmonate compounds are produced and their different modes of action with many important regulatory factors allow plants to respond specifically and flexibly to salt stress (Kurotani et al., 2015a, b; Wasternack and Strnad, 2016). For example, *AtLOX3* involvement has been detected in salinity stress responses (Ding et al., 2016). Constitutive overexpression of *AOC* in wheat leads to elevated JA levels and JA responses, and also confirms the involvement of JA in salinity responses (Cai et al., 2014). The functions of JAZ homologs in the response to salt stress have been identified in many plant species. In addition, it has been shown that the bHLH factors RICE SALT SENSITIVE3 (RSS3)/OsbHLH094 interact with JAZs to regulate tolerance to salt (Toda et al., 2013). Furthermore, overexpression of *OsbHLH148* in a rice mutant induces high expression of *OsDREB* and *OsJAZ* upon drought stress (Seo et al., 2011). Similarly, overexpression of *OsJAZ9* significantly improves salt and drought tolerance in rice (Ye et al., 2009).

Cotton (*Gossypium* spp.), an important economic crop, is sown worldwide to provide fiber material for textile industries. In addition, cotton side products are also used as oil, feed, foodstuffs, and biofuels (Sunilkumar et al., 2006). There are about 50 species in the *Gossypium* genus, but only four species are agriculturally cultivated including two allotetraploids (*G. hirsutum* and *G. barbadense*) and two diploids (*G. herbaceum* and *G. arboreum*) (Wendel, 2000; Hong-Bin et al., 2008). At present, most cotton cultivars grown worldwide are the two allotetraploid species; few cultivars of diploid species are used in production. However, different *Gossypium* species possess various desirable traits or genes, which can be used to improve allotetraploid cultivars although the interbreeding seeds are difficult to obtain among these species through sexual hybridization. Notably, *G. arboreum* has many favorable traits/genes which can confer high resistance to biotic and abiotic stresses. However, *G. arboreum* is not widely sold due to lower fiber quality and yield compared to upland cotton (*G. hirsutum*) cultivars. It is therefore important to explore and identify desirable genes from *G. arboreum* and introduce these genes into upland cotton.

Up to date, in cotton, some JAZ proteins have been researched, which involved in multiple aspects of plant development and defense (Li et al., 2017; Sun Q. et al., 2017). For instance, *GaJAZ1a-like* and *GhJAZ2* can be induced by ABA and NaCl treatment, indicating that they participate in cotton abiotic stress (You et al., 2016; Sun H. et al., 2017). GhJAZ1 can activate the expression of GbWRKY1, which is a critical regulator mediating the plant defense-to-development transition (Li et al., 2014). GhJAZ2 protein was reported that it suppresses fiber initiation by interacted GhMYB25-like suppressing transcriptional activity (Hu et al., 2016), and that it can restrain the defense response by interacting with GhbHLH171 (He et al., 2017).

In this study, we characterized the *G. arboreum JAZ1* function in plant tolerance to salt stress through loss-of-function analysis. GaJAZ1 interacts with GaMYC2 and inhibits its transcriptional activity, resulting in increasing plant tolerance to salt stress. The ectopic overexpression of *GaJAZ1* in *G. hirsutum* cotton significantly increased plant salt tolerance, due to provision of a set of instructions to express salt tolerance-related genes (reprogrammed expression of salt tolerance-related genes) and promotion of root growth. This study provides a foundation for introducing favorable genes of other *Gossypium* species into upland cotton after evaluation and identification.

## Materials and Methods

### Plant Materials and Growth Conditions

Seeds of the receptor material CCRI24 (wild type, WT) and T5 generation of the genetically modified overexpression *GaJAZ1* (*GaJAZ1-OE*) cotton came from the Cotton Institute of the Chinese Academy of Agricultural Sciences, Anyang, China.

To determine expression patterns with different treatments using seedlings grown in Hoagland’s solution, 10 μM jasmonate methyl ester (MeJA), 0.5 mM salicylic acid (SA), 50 mM abscisic acid (ABA), 100 mM auxin, 0.5 mM Ethephon, were added to Hoagland’s solution for the WT plants (3 weeks), alcohol solution was added to 2.5% as the mock. And, 17% PEG6000, or 300 mM NaCl were added to Hoagland’s solution for the WT plants (3 weeks), H_2_O as the mock. In order to test whether high or low temperature can induce the expression of *GaJAZ1*, the WT plants (3 weeks) were placed in an incubator at 40°C or 4°C, respectively. Tissues including root, stem, leaf, seed, pistil, stamen, petal, calycle, −1 DPA ovule, 0 DPA ovule, and 3 DPA, 5 DPA, 10 DPA, 12 DPA, 15 DPA fiber were then harvested at the indicated time intervals for RNA extraction.

The WT and *GaJAZ1*-OE cotton plants were planted at Anyang (Henan, China) in 2014. The WT and *GaJAZ1*-OE cotton plants were planted at the Yellow River Delta (Dongying, Shandong, China) and the Yangtze River Delta (Yancheng, Jiangsu, China) in 2015 and 2016. In 2014, three plot experiments were used in artificial salt plot at Anyang, each plot had three repeats, each repeat had two transgenic lines and the WT, and each line had approximately 20 plants. In 2015, three plot experiments were used in Dongying, each plot had two repeats, each repeat had two transgenic lines and the WT, and each line had approximately 50 plants. The same experiment was performed in Yancheng. In 2016, three different salinity areas were chosen in Yancheng and Dongying, and the experimental designs were the same as those in 2015. The saline concentrations of each test plot in each year, the data on germination rates, seedling biomass, plant heights, and number of bolls were analyzed statistically (Table 1).

**TABLE 1:** The seedling emergence rate, plant height, boll number, and lint yield in transgenic cotton lines and the control.

Year	Zone	Salinity	Line	Seedling Emergence rate (%)	Plant height (cm)	Bolls number	Lint(g)
2014	AY	3.06‰	L1	96.00%^b^	75.0^c^	15.0^d^	28.95^c^
			L2	92.00%^b^	75.0^c^	16.0^c^	29.92^d^
			L3	94.00%^b^	70.0^b^	12.0^b^	21.60^b^
			WT	61.00%^a^	65.0^a^	9.0^a^	15.75^a^
2015	YC	2.38‰	L1	50.0%^a^	106.0^c^	20.0^c^	38.00^c^
			L2	52.0%^a^	92.0^b^	18.0^b^	32.76^b^
			WT	55.6%^a^	85.0^a^	10.0^a^	16.50^a^
	DY	3.12 ‰	L1	38.0%^c^	64.0^c^	11.0^c^	18.81^b^
			L2	26.0%^b^	48.0^b^	13.0^b^	21.58^c^
			WT	20.0%^a^	44.5^a^	9.0^a^	14.04^a^
2016	YC-Z1	2.13‰	L1	83.3%^b^	99.0^c^	38.0^c^	66.50^c^
			L2	79.0%^ab^	87.8^b^	29.0^b^	49.01^b^
			WT	75.0%^a^	80.0^a^	19.0^a^	29.64^a^
	YC-Z2	4.36‰	L1	29.2%^a^	55.0^c^	6.0^c^	7.98^c^
			L2	24.4%^a^	53.0^b^	5.0^b^	6.20^b^
			WT	25.0%^a^	49.7^a^	4.0^a^	4.64^a^
	DY	2.72 ‰	L1	68.8%^b^	113.2^c^	31.0^c^	50.53^c^
			L2	63.2%^b^	108.0^b^	28.0^b^	44.24^b^
			WT	56.3%^a^	91.2^a^	23.7^a^	35.97^a^

*Lint = Boll number × yield/boll. L1, L2, L3, the three transgenic cotton of overexpressing GaJAZ1 plants. Control, receptor material CCRI24. Because L3 had lower yield in 2014, this line was not grown in the 2015 or 2016 trials. Within each column, means that are not followed by the same letter are significantly different according to Tukey’s multiple range test at 0.05 level. The plot means of seedling emergence rate, plant height, boll number, and lint yield value were based on the average over 15 individual plants. AY, Anyang (Henan, China). YC, Yancheng (Jiangsu, China). DY, Dongying (Shandong, China).*

The seeds of WT and *GaJAZ1-OE* genotypes were surface-sterilized with 0.1% mercuric chloride for 5 min and rinsed five times with sterile water. The sterilized seeds were sown on modified MS medium (Murashige and Skoog, 1962) (50 ml/L MS + 25 g/L sucrose + 2.3 g/L agar, pH 5.8 to 6.0), and maintained in a growth chamber (25°C, 16 h light/8 h dark). The WT and the *GaJAZ1-OE* plants were subjected to various treatments: MS, MeJA (10 μM), NaCl (3‰) + MS, NaCl (3‰) + MeJA (10 μM), and both genotypes were treated with MG132 (a proteasome-specific inhibitor in JA signaling research, 100 μM) for 6 h and then planted on MS or NaCl (3‰) + MS media. MS medium was set as a control in this study. Five replicates per group and five-ten individual seedlings per replicate were used.

### Sequence Alignment, Phylogenetic Analysis, and Vector Construction

A phylogenetic tree for *GaJAZ1* (KT312824) was performed using MEGA 6.0, and the modified 35S:*GaJAZ1*:pSuper1300 vector was constructed as described previously (Zhao et al., 2016). The 35S:*GaJAZ1*:GFP vector was constructed and transformed into tobacco leaves by *Agrobacterium tumefaciens* GV3101-mediated transformation. The transformed tobaccos were incubated at room temperature for 24 h with no light, and with normal light (25°C, 16 h light/8 h dark) for 48 h. GFP images were subsequently obtained with a laser scanning confocal microscope (OLYMPUS FV1200).

The pYL156-*GaJAZ1* and pYL156-*GaMYB59* vectors were constructed and transformed into GV3101. The PYL156-*GaJAZ1* and pYL156-*GaMYB59* were injected into the receptor material CCRI24 (WT), separately. Each treatment had at least 45 individual plants. The injected cottons were cultured at 25°C, 16/8 h (day/night). All the materials were treated with 3‰ NaCl after the control pYL156-PDS showed a whitening phenotype. The silencing efficiency was detected by qRT-PCR. The survival rate was the proportion of plants surviving compared to total plants after 300 mM NaCl treatment.

### Yeast Two-Hybrid Assay

Yeast two-hybrid assays were performed using the Clontech yeast two-hybrid system and following the user manual of the Matchmaker Gold Yeast Two-Hybrid System (Clontech Inc.). The cDNAs of *GaJAZ1*, *GaJAZ1*-Δ*Jas*, *AtJAZ1*, and *AtJAZ1*-Δ*Jas* were subcloned into pGBKT7 as bait, while AtMTC2 and putative MYC2-like of cotton cDNAs *GaMTC2.1* and *GaMTC2.2* were subcloned into pGADT7 as prey. The bait and prey plasmids with various combinations were co-transformed into Y2H Gold Yeast strain. Transformed yeast cells were first grown on SD/–Leu/–Trp media for 3 days. To test the interaction between the two proteins, the colonies were picked out and grown on SD/–Ade/–His/–Leu/–Trp agar media supplemented with X-a-Gal and Aureobasidin A.

### Firefly Luciferase Complementation Assays

The CDS of *GaMYC2.1* and *GaMYC2.2* were fused to the C-terminus of the pCAMBIA-cLUC vector, while *GaJAZ1* and *GaJAZ1*-Δ*Jas* were fused to the N-terminus of the pCAMBIA-nLUC vector. Firefly luciferase complementation assays were conducted as previously described (Kong et al., 2015).

### Transient GUS Activity Assays

To detect the transcriptional repression activity of *GaJAZ1*, the CDS of *GaJAZ1* and *GaJAZ1*-Δ*Jas* were fused to GAL4BD to generate GAL4BD-*GaJAZ1/GaJAZ1*-Δ*Jas*, which was then cloned into pBI121 under the control of the 35S promoter. Transient expression assays were performed as previously described (Duan et al., 2017). For the transient analysis of *GaJAZ1* repressing the expression of *GaMYB59*, a 2500bp upstream fragment of *GaMYB59* was cloned in to pBI121 to drive the expression of the GUS reporter gene. The vector was constructed and transformed into tobacco leaves by *Agrobacterium tumefaciens* GV3101-mediated transformation. 14 h before taking GUS staining, MeJA (10 μM), MG132 (a proteasome-specific inhibitor in JA signaling research, 100 μM) was injected into the leaves, and DMSO was used as the mock. Each treatment had at least 5 individual plants, every individual plant was injected at least 3 leaves. The transformed tobaccos were incubated at room temperature for 24 h with no light, and with normal light (25°C, 16 h light/8 h dark) for 48 h. The analysis was performed as previously described (Duan et al., 2017).

### Phenotyping and Physiological Characterization

The WT and *GaJAZ1-*OE plants were grown in a greenhouse (28 ± 3°C/23 ± 3°C, 16 h light/8 h dark) under a light intensity of 600 μmol/m^2^/s and 70% relative humidity. The growth conditions (nutrient medium, water, or NaCl concentration, temperature, humidity, soil composition, and weight) were kept constant throughout the growth period. The following NaCl concentrations were used: 2, 3, and 4‰. Each treatment included at least 30 individual plants. Leaves at the three- to four-leaf stage without excess water and veins were collected to determine chlorophyll a and b content. Each sample had three biological replicates. The amounts of chlorophyll a and b were quantified based on the molar extinction coefficients of these molecules (Lewkowitsch, 1928), each sample was repeated three times. Roots, including lateral roots and root hairs, were removed from the soil as completely as possible, cleaned, photographed, and scanned under a root scanner using WINRHIZO software^2^.

A plant tissue sample (0.2 g) was frozen in liquid nitrogen, ground into powder, and then diluted with homogenate medium to the appropriate concentration for determining antioxidant enzymatic activities (catalase, superoxide dismutase, malondialdehyde, and total antioxidant capacity). Total soluble protein amount was determined using the Bradford method (Bradford, 1976). The contents of catalase (CAT), superoxide dismutase (SOD), malondialdehyde (MDA), and total antioxidant capacity (T-AOC) were measured with ammonium molybdate (Góth, 1991), pyrogallol autooxidation (Brown et al., 1997), thiobarbituric acid (TBA) (Guillensans and Guzmanchozas, 1995), and phenanthroline (Benzie and Strain, 1996), respectively.

### RNA-Seq and Data Analysis

Wild type and *GaJAZ1*-OE plants were planted with culture conditions based on the Hoagland solution (Tocquin et al., 2003), with minor modifications (Supplementary Table S1). Cultures were kept under a 16 h light/8 h dark cycle and a relative humidity of 60%. Young leaves, stems, and roots were sampled at the three- to four-leaf stage; plants treated with 3‰ NaCl solution or water (control) for 0, 6, 12, and 24 h were collected for RNA extraction. Samples were obtained from three individual plants for each stage. A total of 3 μg RNA per sample was used for the RNA sample preparations. Sequencing libraries were generated using NEBNext Ultra^TM^ RNA Library Prep Kit for Illumina (NEB, United States) following the manufacturer’s recommendations and index codes were added to attribute sequences to each sample. The fragments per million mapped reads per kilobase (FPKM) for each gene was calculated using the HTSeq v0.6.1 (Trapnell et al., 2012), which was used to map to upland cotton transcripts with the aid of the *G. hirsutum* genome annotation (Zhang et al., 2015).

Differential expression analysis after treatment with control and NaCl was performed using the DESeq R package (1.18.0) (Wang and Cairns, 2014). Genes with an adjusted *P*-value < 0.05 found by DESeq were assigned as differentially expressed genes (DEGs). Gene Ontology (GO) enrichment analysis of DEGs was implemented in the GOseq R package (Wang and Cairns, 2014). GO terms with corrected *P*-value less than 0.05 were considered significantly enriched by DEGs. KOBAS software (Mao et al., 2005) was used to test the statistical enrichment of DEGs in KEGG pathways^3^.

### Accession Numbers

Cotton gene names and identifiers referred to in this article are:

AtJAZ1 (AT1G19180), AtJAZ2 (AT1G74950), AtJAZ5 (AT1G17380), AtJAZ6 (AT1G72450), AtMYC2 (AT1G32640), GaJAZ1 (Cotton_A_11862), GaJAZ5 (Cotton_A_18896), GaJAZ9 (Cotton_A_09418), GaJAZ10 (Cotton_A_27840), GaMYC2.1 (Cotton_A_07316), GaMYC2.2 (Cotton_A_07908), GaMYB59 (Cotton_A_10538), GaLOX3 (Cotton_A_04987), GaAOS (Cotton_A_02059), GaAOC4 (Cotton_A_12629), GaACS6 (Cotton_A_32717), GaERF2 (Cotton_A_38436), GaERF4 (Cotton_A_33523), GaABR1 (Cotton_A_25067), GaABA2 (Cotton_A_10162), GaCBF4 (Cotton_A_39051), GaRD26 (Cotton_A_06324), GaCIPK9 (Cotton_A_20598), GaMYB13 (Cotton_A_14960), GahDFL2 (Cotton_A_14381), GhJAZ2 (CotAD_46116), GhJAZ8 (CotAD_26962), GhJAZ11 (CotAD_09712), GhJAZ13 (CotAD_00351), GhJAZ15 (CotAD_ 06544), GhJAZ17 (CotAD_21952), GhJAZ18 (CotAD_62298), GhJAZ22 (CotAD_22999), GhMYB59 (Gh_D13G2393),_ GhLOX3 (Gh_A06G1802), GhAOS (Gh_D12G0393), GhACS6 (Gh_A12G2673), GhERF2 (Gh_A05G1332), GhERF4 (Gh_D01 G0450), GhABR1 (Gh_D10G1019), GhABA2 (Gh_A01G2141), GhCBF4 (Gh_D12G2494), GhRD26 (Gh_A04G1303), GhG H3.6 (Gh_A01G0547), GhCIPK9 (Gh_Sca010555G01), GhMY B13 (Gh_A04G0921), GhDFL2 (Gh_D11G1005), GrJAZ4 (Cotton_D_10010230), GrJAZ6 (Cotton_D_10033602), GrJAZ8 (Cotton_D_10037314), GrJAZ10 (Cotton_D_10031747), GrJA Z13 (Cotton_D_10039035), GrJAZ15 (Cotton_D_10023394), PtJAZ2 (POPTR_0001s16640), PtJAZ3 (POPTR_0003s06670), PtJAZ6 (POPTR_0006s14160), GmJAZ1 (GLYMA_01G204400), GmJAZ4 (GLYMA_07G041400), GmJAZ7 (GLYMA_09G07 1600), GmJAZ11 (GLYMA_11G038600), GmJAZ12 (GLYMA_ 13G112000), GmJAZ16 (GLYMA_15G179600), GmJAZ18 (GLYMA_16G010000), GmJAZ20 (GLYMA_17G047700).

RNA-seq data, including gene accession numbers, are available in the NCBI SRA under accession number PRJNA439356.

### DNA/RNA Isolation and Quantitative Real-Time PCR Analysis Confirmation of Salt-Responsive Genes

Protocols to isolate DNA or RNA and the quantitative real-time PCR were followed as described previously (Zhao et al., 2016). Quantitative real-time PCR was used to confirm the expression level of the selected DEGs. The gene-specific primers are listed in Supplementary Table S2.

### Statistical Analysis

Student’s *t*-test and Tukey’s ANOVA test was used to perform all statistic analyses of data to generate *P*-value. The data represent the mean ± SD of *n* ≥ 3 independent experiments. All the tests were two-tailed. The data were normalized, and all samples were normally distributed with homogeneity of variance.

## Results

### Identification and Expression Pattern of *GaJAZ1*

**FIGURE 1:**
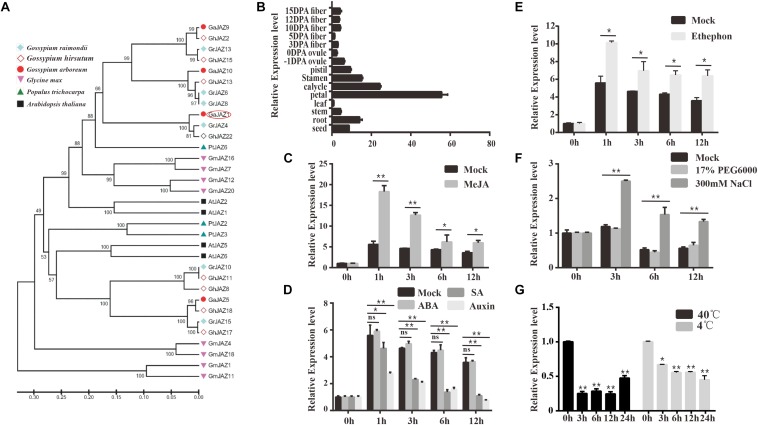
Identification and expression pattern of *GaJAZ1* in cotton. **(A)** Phylogenetic analysis of *GaJAZ1* in cotton with *JAZ1* from other five species using the MEGA 6.0 program. JAZ1 sequences from *Gossypium raimondii*, *G. hirsutum*, *Gossypium arboreum*, *Glycine max*, *Populus trichocarpa*, and *Arabidopsis thaliana* were used to construct the phylogenetic tree. **(B)** Expression level of *GaJAZ1* in 15 tissues. DPA, days post anthesis. **(C)** Expression level of *GaJAZ1* in roots at 0, 1, 3, 6 and 12 h after treating with 10 μM MeJA, the 2.5% alcohol solution as the mock. **(D)** Expression level of *GaJAZ1* in roots at 0, 1, 3, 6, and 12 h after treating with 0.5 mM SA, 50 mM ABA, and 100 mM auxin, the 2.5% alcohol solution as the mock. **(E)** Expression level of *GaJAZ1* in roots at 0, 1, 3, 6, and 12 h after treating with 0.5 mM ethephon, the 2.5% alcohol solution as the mock. **(F)** Expression level of *GaJAZ1* in roots at 0, 3, 6, and 12 h after treating with 17% PEG6000 and 300 mM NaCl, the H_2_O as the mock. **(G)** Expression level of GaJAZ1 in roots at 0, 3, 6, 12, and 24 h after high temperature treatment (40°C) and low temperature treatment (4°C). Bar represents standard deviation (SD) of three independent biological replicates. The single asterisk indicates statistical significance at *P* < 0.05. The double asterisk indicates statistical significance at *P* < 0.01.

*Gossypium arboreum* has mostly been with drawn from the market because of its lower quality and yield of fiber in comparison with *G. hirsutum*, but it possesses potentially higher tolerance to abiotic stresses including salinity. Our previous study showed that *GaJAZ1* belongs to the JAZ subfamily based on the presence of TIFY and Jas motifs, and is considered to participate in plant resistance to abiotic stresses (Zhao et al., 2016). The reconstructed phylogenetic tree of JAZs (Figure 1A) from six plant species confirms this result. Similarities of *GaJAZ1* to *JAZ1* from other plant species were high based on sequence alignment and TIFY and Jas motif identification (Supplementary Figure S1). Results of qPCR analysis showed that *GaJAZ1* was preferentially expressed in flower parts and roots (Figure 1B), almost consistent with the results in upland cotton obtained from the database (Supplementary Figure S2) (Zhang et al., 2015).

To determine whether *GaJAZ1* is associated with the JA signaling pathway, hormone-induced expression analyses were carried out. *GaJAZ1* was significantly induced by MeJA in roots compared to the mock (Figure 1C). Given that JA signaling can be affected by a crosstalk of other hormones, we performed the analyses of plant response to SA, auxin, ABA and ethylene. The *GaJAZ1* expression levels in roots treated with SA or auxin was significantly reduced at 1, 3, 6 and 12 h compared to the mock, whereas ABA treatment did not change the *GaJAZ1* expression level (Figure 1D). *GaJAZ1* was induced by ethylene in roots compared to the mock (Figure 1E). Thereby, supposing that *GaJAZ1* has a regulatory role in JA signaling, these results raise a possibility that such signaling is modulated depending on response to JA response itself and other hormones.

JAZs have been reported to regulate plant tolerance to abiotic stresses (Wasternack and Strnad, 2016). To determine the function of *GaJAZ1* in stress tolerance, the expression responses to salt, drought, and extreme temperature stresses were examined. The results showed that the *GaJAZ1* expression in roots significantly increased under NaCl treatment compared to the mock over time, whereas polyethylene glycol (PEG) treatment did not affect the gene expression, suggesting that *GaJAZ1* was not involved in plant tolerance to drought (Figure 1F). When plants were placed into a 4°C or 40°C light incubator, the *GaJAZ1* expression significantly decreased at 3, 6, 12, and 24 h compared to the control in roots (0 h treated sample in a 25°C incubator; Figure 1G) and leaves (Supplementary Figure S3). These results indicated that *GaJAZ1* potentially participates in plant tolerance to various abiotic stresses, especially salt stress.

### Knockdown of *GaJAZ1* Reduced Plant Tolerance to Salt Stress

**FIGURE 2:**
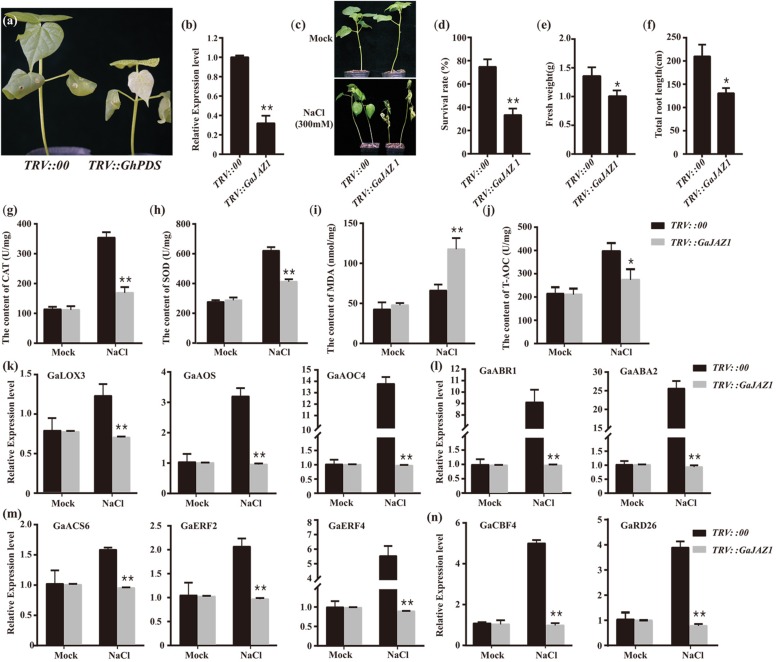
Knockdown of *GaJAZ1* reduced the tolerance to salt stress. **(a)** The phenotypes of *TRV:PDS* plants. *TRV*, tobacco rattle virus. **(b)** The silencing efficiency of *GaJAZ1* in*TRV:00* (*n* = 43) and *TRV:GaJAZ1* plants (*n* = 43). **(c)** The phenotypes of *TRV:00* and *TRV:GaJAZ1* plants 16 days after treatment with 300 mM NaCl, the H_2_O as the mock. **(d–f)** The survival rates, fresh weight and the root length in *TRV:00* (*n* = 43) and *TRV: GaJAZ1* (*n* = 37) plants after treatment. **(g–j)** The activities of catalase (CAT) and superoxide dismutase (SOD), malondialdehyde (MDA) contents and antioxidant capacity (T-AOC) in roots in *TRV:00* and *TRV:GaJAZ1* plants after treatment. **(k–n)** Quantitative real-time PCR analysis of the expression patterns of key genes involved in salt stress responses in *TRV:00* and *TRV:GaJAZ1* plants after treatment with 300 mM NaCl. Bars represent SD of three independent biological replicates and four technical repeat experiments. The single asterisk indicates statistical significance at *P* < 0.05. The double asterisk indicates statistical significance at *P* < 0.01.

To further evaluate the role of *GaJAZ1* in plant tolerance to salt stress, loss-of-function plants should be developed. However, to date, the transformation technique has not yet been developed in *G. arboreum*. The virus-induced gene silencing (VIGS) method is a robust tool to dissect gene function, especially defense-related genes (Burch-Smith et al., 2006; Choong-Min et al., 2010). *GaJAZ1*-silenced plants were therefore generated using the VIGS technique in this study. Fifteen days after agro-infiltration, the *PDS*-silenced plants as a marker control showed a photobleaching phenotype in emerging leaves, indicating that VIGS was effective in cotton (Figure 2a). At this time point, *GaJAZ1* expression in the silenced plants was strongly inhibited, at only 0.32-fold of the level in control plants injected with empty vector pYL156 (Figure 2b). The *GaJAZ1*-silenced plants showed comparable phenotypes with the control during the entirety of vegetative development of plants.

*GaJAZ1*-silenced plants were treated with 300 mM NaCl to test their tolerance to salinity stress. The phenotypes of the control and silencing plants are similar under mock treatment, while the silencing *GaJAZ1* plants treated with NaCl showed more serious wilt compared to the control (Figure 2c). Sixteen days after treatment, the survival rate of silenced plants was 33.3%, a significant decrease compared to the control with 74.7% survival (Figure 2d). The fresh weight and the root length of silenced plants treated by NaCl solution were significantly decreased (Figures 2e,f).

When plants are challenged by environmental stress, their physiology and biochemistry changes. To identify the mechanism of salt tolerance in *GaJAZ1*, physiological and biochemical analysis including reactive oxygen species (ROS) scavenging enzymes, MDA, and T-AOC were performed in plants treated with NaCl solution at 3 days after treatment. The activities of two ROS scavenging enzymes, CAT and SOD, significantly decreased in *GaJAZ1*-silenced plant roots compared to the control (Figures 2g,h). The MDA content in silenced plants was significantly higher than that in the control, indicating that the knockdown of *GaJAZ1* increases plasma membrane damage under salt stress (Figure 2i). T-AOC activity in roots of *GaJAZ1* knockdown plants was significantly lower than that in the control (Figure 2j). In addition, physiological and biochemical changes in leaves of *GaJAZ1*-silenced plants treated with NaCl solution were consistent with the results in root samples (Supplementary Figure S4). These results suggested that knockdown of *GaJAZ1* can decrease CAT, SOD and T-AOC activities and increase cell membrane damage, possibly due to changes of cell physiological and biochemical balance under salinity stress.

To evaluate the mechanism of *GaJAZ1* function in plant tolerance to salt stress, the expression profiles of salt tolerance-related genes were monitored. We firstly examined expression of these genes involved in plant hormone pathways, including ethylene, JA, and ABA. *GaLOX3*, *GaAOS* and *GaAOC4* (JA-related genes), *GaACS6*, *GaERF2*, and *GaERF4* (ethylene-related genes) and *ABR1* and *ABA2* (ABA-related genes) showed significantly down-regulated expression in silenced plants treated with NaCl solution compared to the control (Figures 2k–m). The expression of salt-related marker genes *CBF4*, and *RD26* significantly decreased in knockdown plants challenged by salt stress (Figure 2n). Collectively, *GaJAZ1* likely participates in plant resistance to salinity stress, resulting in reprogrammed salt-tolerant gene expression when plants were challenged by NaCl stress, possibly involving JA, ethylene, and ABA signaling pathways or crosstalk of these hormone pathways.

### Interaction Partner and Repression Activity of GaJAZ1

**FIGURE 3:**
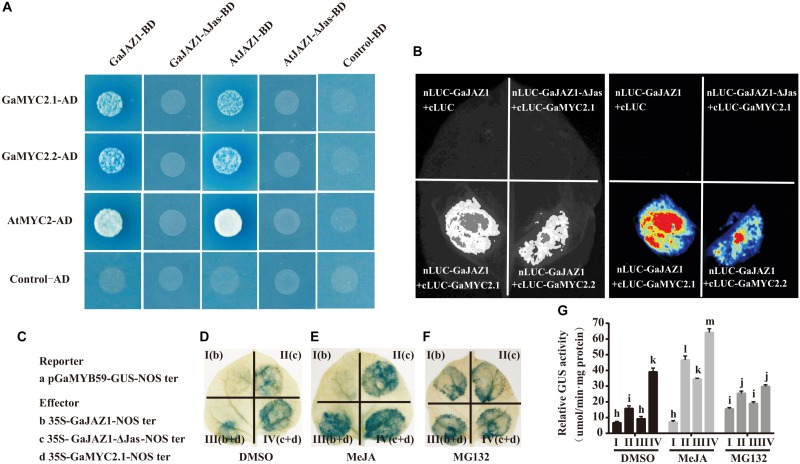
Interaction partner and repression activity of GaJAZ1. **(A)** Yeast two-hybrid assays. *GaMYC2.1* and *GaMYC2.2* are two orthologous genes of *AtMYC2*. GaJAZ1-ΔJas/AtJAZ1-ΔJas lacks the Jas domain region. The interactions were determined by growth on SD/–Ade/–His/–Leu/–Trp agar media supplemented with X-a-Gal and Aureobasidin A. Mating with pGBKT7 empty vector was used as the negative control. **(B)** GaJAZ1 interacts with GaMYC2.1 and GaMYC2.2 in a split firefly luciferase complementation assay. GaMYC2.1 and GaMYC2.2 fused to the C-terminus of LUC (cLUC-GaMYC2.1/GaMYC2.2) was co-expressed with a GaJAZ1-fused N-terminus of LUC (nLUC-GaJAZ1) in *Nicotiana benthamiana* leaves. Images were collected 3 days after infiltration. **(C–G)** GaJAZ1 repressing GaMYC2 transcriptional activity. **(C)** A 2 kb upstream fragment of *GaMYB59* was obtained by genomic sequence alignment and PCR cloning as a promoter of *GaMYB59* (p*GaMYB59*), p*GaMYB59* fragment driving GUS was used as reporter a; the b–d are different effectors. The panels **(D–F)** were the different treatment, MeJA, MG132, DMSO, respectively. MeJA, jasmonate methyl ester. MG132, a proteasome-specific inhibitor in JA signalling research. DMSO was used as the mock. Panel I (b) and panel II (c) represent reporter was co-injected with effector b, effector c into a tobacco leaf, respectively. Panel III (b+d) and panel IV (c+d), represent reporter was co-injected with effector b and d, effector c and d, into a tobacco leaf, respectively. **(G)** The GUS activity in Panel I, II, III, III in **(D–F)** measured by transient GUS activity assays. Bars represent SD of three independent biological replicates and four technical repeat experiments (Tukey’s multiple range test).

Given that *GaJAZ1* participates in plant tolerance to salt stress, the molecular mechanism was explored. The *GaJAZ1* distribution in cells was firstly identified using a transient expression system in tobacco. *GaJAZ1* was mainly localized in the nucleus through subcellular localization of the GFP fusion protein (Supplementary Figure S5). In the JA signal pathway, *JAZ1* may repress expression of downstream genes by binding to MYC2 (Thines et al., 2007). GaMYC2.1 and GaMYC2.2, orthologs of AtMYC2, were identified, and the results of a yeast two-hybrid assay showed that GaMYC2.1 and GaMYC2.2 interacted with GaJAZ1 protein (Figure 3A). In a parallel experiment, Arabidopsis MYC2 strongly interacted with AtJAZ1. Cotton JAZ1 can interact with Arabidopsis MYC2 and AtJAZ1 can interact with GaMYC2.1 and GaMYC2.2. When the Jas domain in GaJAZ1 and AtJAZ1 was mutated (designated GaJAZ1-ΔJas or AtJAZ1-ΔJas), there was no interaction between JAZ1 and MYC2. The same results were shown by two fluorescence complementarity assays (Figure 3B), suggesting that the GaJAZ1 interaction with GaMYC2 in cells may determine the expression levels of downstream genes.

To determine the transcriptional repression activity of GaJAZ1 to GaMYC2, the GUS fusion reporter was analyzed. Previous reports showed that the TF MYC2 can bind G-box *cis*-elements and regulate the expression of downstream genes (Cheng et al., 2011; Wasternack and Hause, 2013). For example, the *AtMYB59* gene can be regulated by the MYC2 TF acting in the G-box of its promoter (Wasternack and Hause, 2013). The promoter of the *GaMYB59* gene, an orthologous gene of *AtMYB59*, was cloned, and replaced the CaMV35S promoter in the pBI121 vector, generating a pGaMYB59:GUS reporter vector, and the GaJAZ1 and GaMYC2.1 effecter vectors were constructed (Figure 3C). The leaves injected with pGaMYB59:GUS reporter vector showed normal GUS staining color, while GaMYC2.1 effecter vector was co-injected, the blue color of stained leaves was deepen, suggesting that GaMYC2.1 can promote *GaMYB59* expression (Supplementary Figure S6). When the GaJAZ1 effector vector was co-injected with reporter vector, there was weak GUS staining present in the injected spot (Figure 3D, panel I), suggesting that GhJAZ1 possibly inhibited MYC2 transcriptional activity in tobacco. When the GaJAZ1-ΔJas vector was co-injected, the GUS staining color was more intense than that co-injected with GhJAZ1 (Figure 3D, panel II). When GaMYC2.1 was transiently co-expressed in tobacco leaf, the density of GUS staining color in GaJAZ1 co-injected leaf decreased compared to the GaJAZ1-ΔJas co-injected leaf (Figure 3D, panel III and IV).

Based on reports that JAZ1 can be degraded through JA induction and accumulated by exogenous application of MG132, which had been reported as a proteasome-specific inhibitor in JA signaling research (Chini et al., 2007), we supposed that JA can promote GaJAZ1 degradation and MG132 can accumulate GaJAZ1 protein in cell (Thines et al., 2007). To further confirm GaJAZ1 repressing transcriptional activity of GaMYC2, two parallel experiments were preformed including JA induced treatment and MG132 inhibitor treatment. As expected, JA treatment effectively released JAZ1 repression and strengthened the GUS staining color in the treated leaves (Figure 3E), while MG132 treatment to some extent changed GUS staining color (Figure 3F). Combined with these results, the GUS activities in different treated leaf parts were examined by 4-methylumbelliferone (4-MU) substrate reaction (Figure 3G). These results confirmed that GaJAZ1 can repress transcriptional activity of GaMYC2 to regulate the expression of downstream genes in the JA signal pathway.

### Ectopically Overexpressing *GaJAZ1* in *G. hirsutum* Enhances Plant Salt Tolerance

Given that *GaJAZ1* participates in plant tolerance to salinity stress, we introduced it into the *G. hirsutum* genome for breeding salt-tolerant cultivars. *G. hirsutum* cannot sexually cross with *G. arboreum* to perform gene exchange. The genetic transformation method is a great tool to carry out gene flow between plant species. Eight independent transgenic *G. hirsutum* lines with overexpressing *GaJAZ1* (*GaJAZ1-*OE) were obtained using the *Agrobacterium*-mediated transformation method. Based on semi-RT-PCR analysis, *GaJAZ1* expression levels were higher in three independent lines (Lines 2, 4, and 5; Supplementary Figure S7). The three transgenic lines seemed to be comparable aboveground phenotypes with WT and showed similar fiber quality in the field (Supplementary Figure S8 and Supplementary Table S3). Thereby, the three lines were employed to perform all the following experiments.

**FIGURE 4:**
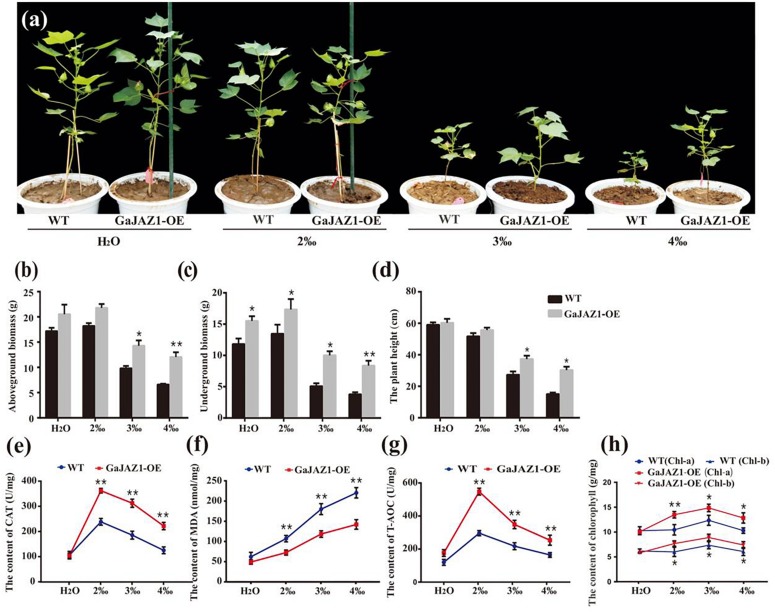
Overexpression of *GaJAZ1* enhances tolerance to salt stress under greenhouse conditions. **(a)** The phenotypes of WT and *GaJAZ1*-OE plants in the greenhouse after treatment with 2‰, 3‰, or 4‰ NaCl during the developmental period; the control was treated with H_2_O, photos were taken 70 days after germination. Both phenotypic and physiological data of the three independent lines were similar. Here, we presented a representative plant/line for exhibiting. **(b–d)** The aboveground biomass, underground biomass, and plant height of WT and *GaJAZ1*-OE plants after treatment. **(e–h)** The activity of catalase (CAT), malondialdehyde (MDA) contents, antioxidant capacity (T-AOC), and levels of chlorophyll a/b in WT and *GaJAZ1*-OE plants after treatment. Bar represents SD of three independent biological replicates. The single asterisk indicates statistical significance at *P* < 0.05. The double asterisk indicates statistical significance at *P* < 0.01.

Since salt stress inhibits seed germination, seed germination analysis was carried out first. The results showed that the seed germination rates of *GaJAZ1*-OE lines were similar to the WT at 4‰ NaCl solution, while they were significantly higher in NaCl solution above 6‰ (Supplementary Figure S9). The transgenic and WT plants were grown under various NaCl content soils (for simulating naturally saline soil) for their whole growth period. Without salinity stress, we found that the growth of *GaJAZ1*-OE plants in the pots seems to be more vigorous than the control plants under the conditions unlike those in the field. Under 2‰ treatments, there were minimal differences between the growth of transgenic and WT plants, indicating that the cotton plant itself is moderately salt-tolerant (Figure 4a). However, the growth potential of *GaJAZ1-OE* was significantly better than WT in 3‰ and 4‰ salt soils (Figure 4a). The phenotypes at three time points of cotton grown under various NaCl soils confirmed that *GaJAZ1*-OE lines grew better than the WT (Supplementary Figure S10). The biomass of the aboveground tissues of *GaJAZ1-*OE was slightly higher than WT plants at H_2_O and 2‰ NaCl conditions, while there were significant differences in soils above 3‰ NaCl (Figure 4b). The underground biomass of *GaJAZ1-*OE was significantly higher in H_2_O and salt soils compared to the WT, indicating that *GaJAZ1* not only increases plant tolerance to salt stress but also promotes root development and growth (Figure 4c). Overexpressing *GaJAZ1* can significantly increase plant height when NaCl is above 3‰ compared to the WT (Figure 4d). These results suggested that over-expressing *GaJAZ1* enhanced plant tolerance to salt stress.

To analyze the *GaJAZ1* mechanism in salt tolerance, a series of physiological changes in *GaJAZ1*-OE lines were measured when planted in soils with various salt contents. Under NaCl soils, the 2-week-old transgenic plants had significantly higher CAT activity than WT plants (Figure 4e). The MDA content of *GaJAZ1*-OE plants was significantly lower than in WT plants under the NaCl soils (Figure 4f). The T-AOC of *GaJAZ1*-OE plants was higher than in WT plants grown in NaCl soils (Figure 4g). Compatible with these results, the contents of both chlorophyll a and b in the *GaJAZ1*-OE leaves were significantly higher than those in WT plants (Figure 4h). The results indicated that the *GaJAZ1*-OE plants can more strongly change their physiological status to enhance salt tolerance compared to the control.

In addition, to investigate whether overexpressing *GhJAZ1* can increase plant tolerance to salt stress, *GhJAZ1*-OE plants were developed. The upland cotton JAZ1 (*GhJAZ1*) has high identity with *GaJAZ1*: only three amino acids differ between them (Supplementary Figure S11). The results of salt stress analysis showed that five independent *GhJAZ1*-OE plants possessed slightly increased salinity tolerance compared to the WT (Supplementary Figure S12), which suggested that *GaJAZ1*, not *GhJAZ1*, can be regarded as a foreign salt tolerance gene to use in upland cotton breeding.

### Overexpression of *GaJAZ1* Promotes Root Growth

**FIGURE 5:**
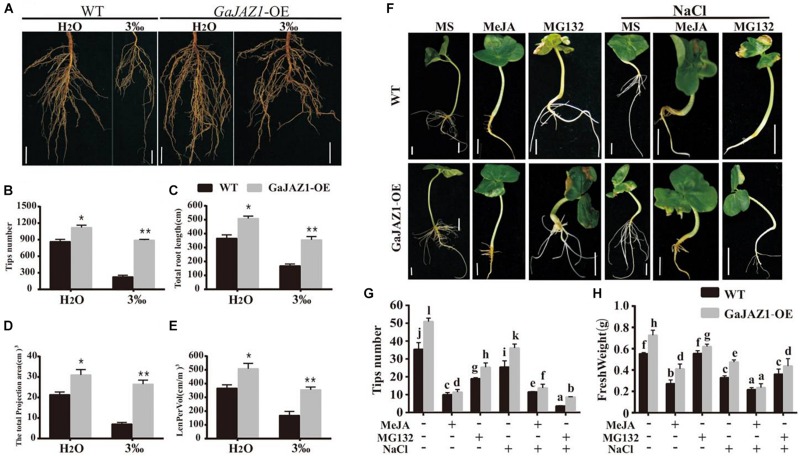
Overexpression of *GaJAZ1* promotes roots growth. **(A)** The root phenotypes of WT and *GaJAZ1*-OE plants treated with H_2_O and 3‰ NaCl solution. Bars = 2cm. **(B–E)** The root tips number, total root length, total projection area, and LenPerVol (total root length of the unit soil volume) of WT and *GaJAZ1*-OE plants after treatment. Bars represent SD of three independent biological replicates. The single asterisk indicates statistical significance at *P* < 0.05. The double asterisk indicates statistical significance at *P* < 0.01. **(F)** The phenotype of cotton seedlings with the treatment of MS (Murashige and Skoog), 10 uM MeJA, 100 uM MG132, and their combinations with 3‰ NaCl for 8 days. Bars = 1 cm. MeJA, jasmonate methyl ester. MG132, a proteasome-specific inhibitor in JA signalling research. MS was used as the mock. **(G,H)** The root tips number, fresh weight of WT and *GaJAZ1*-OE seedlings in **(F)**, respectively. MS was used as the negative control. Bars represent SD of at least 10 independent biological replicates and four technical repeat experiments (Tukey’s multiple range test).

Results of the above analysis indicated that the underground part of the *GaJAZ1*-OE plants grew better compared to WT (Figure 4c). Further analysis showed that root systems of *GaJAZ1*-OE plants were more developed than those of WT including longer roots and a higher density of lateral roots in H_2_O-treated soil (Figure 5), which suggests that *GaJAZ1* can promote root development and growth, consistent with previous reports in Arabidopsis (Grunewald et al., 2009; Zhu et al., 2012). When exposed to 3‰ NaCl soil, WT plant root architecture including root length and density was strongly inhibited compared to H_2_O-treated plants. In contrast, the root system of *GaJAZ1*-OE plants treated with salt was moderately inhibited compared to the WT, indicating that overexpressing *GaJAZ1* can attenuate salt damage to root growth and development (Figure 5A). The number of root tips, primary root length, projection area, total root length of the unit soil volume (LenPerVol), root volume, and other indicators of root development were higher in *GaJAZ1*-OE plants than WT (Figures 5B–E and Supplementary Figure S13). Therefore, *GaJAZ1*-OE plants had stronger salt tolerance partially due to better development of its roots and root hairs.

Arabidopsis *JAZ1* had been previously reported to promote lateral root growth (Grunewald et al., 2009). Thereby, we further studied *GaJAZ1* function in root growth and development, which can contribute to plant tolerance to salt stress. As shown in Figure 5F, a 100 μM MeJA treatment had a stronger inhibitory effect on root growth of WT seedlings, especially lateral root density compared to the control (MS medium); while overexpression of *GaJAZ1* attenuated the JA inhibitory effect on root growth. However, 10 μM MeJA treatment resulted in similar root growth and development between transgenic plants and the WT. Application of MG132, a potent, non-specific proteasome inhibitor, can promote root growth and development of the transgenic plants and WT, consistent with a previous report (Thines et al., 2007). We further examined the effect of *GaJAZ1*-OE on salt stress responses in this system by adding NaCl to the medium (3‰). The results showed that the transgenic seedlings had a better root system compared to the WT with MeJA or MG132, partially resulting in higher tolerance to salt stress. The data on tips number and fresh weight in these plants were consistent with the phenotypic data we described (Figures 5G,H). These results further confirmed that *GaJAZ1* can promote root growth and development, similar with Arabidopsis *JAZ1* function and partially contributing to plant salt tolerance.

### Overexpression of *GaJAZ1* Enhances Plant Salt Tolerance in Saline-Alkaline Fields

To extensively evaluate the tolerance of *GaJAZ1*-OE lines to salt stress, transgenic and WT plants were sown in artificial salt soils (3.06‰ NaCl) in Anyang city in 2014. The three *GaJAZ1*-OE lines showed better growth including taller plants, more bolls, and more roots compared to the WT over the whole life cycle. Results of statistical analysis showed that the three transgenic lines had significantly higher seedling emergence rates, plant height, bolls, and yields compared to the WT (Table 1), which confirmed that overexpressing *GaJAZ1* confers *G. hirsutum* plant tolerance to salt stress.

To further investigate whether the *GaJAZ1*-OE plants can adapt to grow in native saline-alkaline soils, the two transgenic lines were directly sown in five fields with various natural saline-alkaline contents, located in Dongying (DY) city of Shandong province and Yancheng (YC) city of Jiangsu province, in 2015 and 2016. Compatible with the artificial salt soil, in different native saline-alkaline fields, the two *GaJAZ1*-OE lines grew better than the WT (Supplementary Figure S14). Seedling emergence rates, plant heights, boll numbers, and yield of *GaJAZ1*-OE plants and the WT in 2 years in five regions were investigated and statistically analyzed. The results showed that plant heights, boll numbers, and yield in two *GaJAZ1*-OE lines were significantly higher than in the WT (Table 1), while seedling emergence rates showed higher compared to the WT, not consistent with the results in artificial salt soils of Anyang city. In 2015, the DY region contained higher salt content than the YC region, but in both area the two transgenic lines showed higher salt tolerance compared to the WT. Combined with these results, in 2016, two transgenic lines in three areas with different salt contents also exhibited higher tolerance compared to the WT. These data indicate that the two transgenic lines have good salt tolerance and can be sown in moderate saline-alkaline fields for cotton production.

### RNA Sequencing of *GaJAZ1*-OE and WT Reveals That JA-Related Genes Prime Plant Salinity Tolerance

To explore the molecular mechanism of the role of *GaJAZ1* in salt tolerance, the transcriptomes of *GaJAZ1*-OE and WT plants grown in hydroponic culture with 3‰ NaCl were sequenced. The samples were collected from NaCl treated seedlings after 0, 6, 12, or 24 h with three biological replicates for RNA-seq analysis. All data is shown in Supplementary Tables S4–S7. The sample correlation analysis *R*^2^ was more than 0.85, indicating that the transcriptome data was reliable (Supplementary Figure S15a). We identified 1146 differentially expressed genes (DEGs) between the *GaJAZ1*-OE and the WT at all four time-point treatments (FDR adjusted *P*-value < 0.05, | log2 fold-change| ≥ 2) (Supplementary Figure S15b), among which 358 DEGs were down-regulated and 788 DEGs were up-regulated. For all screened DEGs, biological process and molecular function were highly represented between *GaJAZ1*-OE and WT. Molecular function enrichment analysis revealed that the group was mainly enriched in enzyme activity, molecular role, ion binding, response to oxidative stress, and oxidation-reduction process based on corrected *p*-value < 0.05 (Supplementary Figure S15c).

**FIGURE 6:**
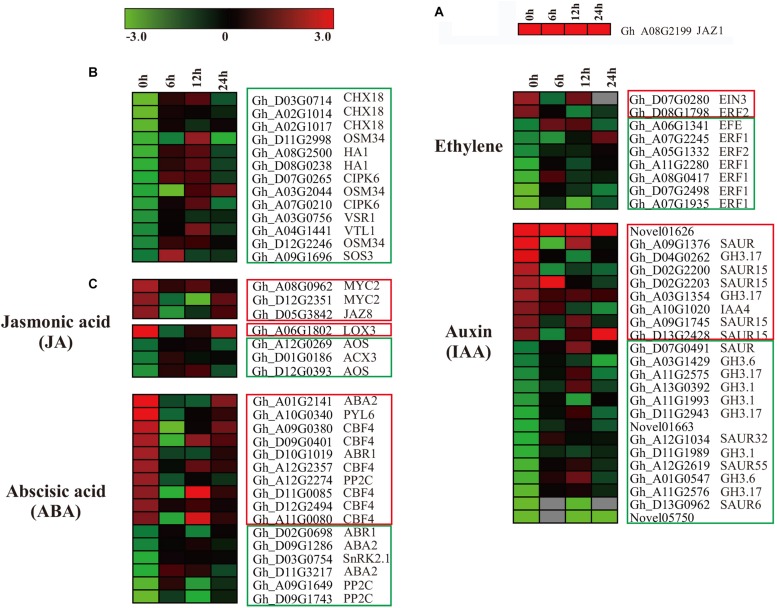
The differentially expressed genes assigned to hormone signal transduction pathways, and the genes related to salt stress in the WT and *GaJAZ1*-OE plants. **(A–C)** Heatmap of the differentially expressed genes assigned to hormone signal transduction pathways, and the genes related to salt stress in the WT and *GaJAZ1*-OE plants of the transcriptome data (FDR adjusted *P*-value < 0.05, | log2 fold-change| ≥ 1.5). The log-transformed expression values range from –3 to 3. Red and blue colors indicate up- and down-regulated transcripts, respectively. The genes in red boxes are up-regulated without treatment, and the genes with a blue solid line are down-regulated.

In our data, ectopic expression of *GaJAZ1* level (contained in *GhJAZ1*, Gh_A08G2199), as a marker (Figure 6A) in *GaJAZ1*-OE plants was significantly higher compared to the WT. In the RNA-seq data, overexpressing *GaJAZ1* affected the expression levels of *G. hirsutum* JAZ genes, including *JAZ1/3/6/8/10*. *JAZ1, JAZ3*, *JAZ6*, *JAZ8*, and *JAZ10* were up-regulated without NaCl treatment (0 h treatment) compared to the WT, but it was surprised that for 6 h and 12 h treatment, thesegenes were down-regulated, for 24 h treatment, their expression levels upregulated, indicated that under NaCl treatment, these genes’ expression was sophisticated in *GaJAZ1*-OE plants (Supplementary Figure S16a). In the JA signal pathway, expression levels of most *MYC2* significantly upregulated in *GaJAZ1*-OE plants compared to the WT without NaCl treatment, *JAR1* was downregulated expression (Supplementary Figure S16b). These results suggested that overexpressing *GaJAZ1* can affect the expression of JA signal pathway components in upland cotton. However, under NaCl treatment, these genes’ expression took place sophisticated changes, which reasoned that JA-related genes’ expression are possibly affected by not only overexpressing *GaJAZ1* but also other factors, including hormone-crossing signal and salt-inducible genes. Additionally, the expression changes of JA synthesis-related genes, including *LOXs*, *AOS*, *AOC4*, *ACXs*, *MFP2*, and *JMT*, were detected between *GaJAZ1*-OE plants and the WT without NaCl treatment (Supplementary Figure S16c), which indicated that overexpressing *GaJAZ1* affected JA biosynthesis.

According to our RNA-seq data, most salt stress-related genes showed differential expression between *GaJAZ1*-OE plants and the WT. As shown in Figure 6B, two vacuolar-associated proteins (*VTL1*, *VSR1*), an osmotic protein (*OSM34*), two plasma membrane ion exchange related genes (*HA1*, *CHX18*), and salt-response genes (*CIPK6*, *SOS3*) showed significantly down-regulated expression without NaCl treatment, which suggested that ectopic expression of *GaJAZ1* resulted in the accumulation of osmolytes that help to re-establish the osmotic balance and protect the plant from salt stress damage. While at 12 h NaCl treatment, the expression levels of most genes significantly increased (Figure 6B), indicating that salinity tolerance genes are regulated by many factors including overexpressing *GaJAZ1* through mediating the osmotic and ionic homeostasis.

**FIGURE 7:**
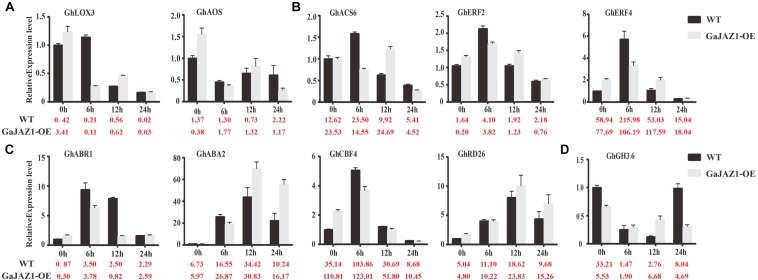
Quantitative real-time PCR analysis of the expression patterns of key genes involved in salt stress responses after treatment with 3‰ NaCl. The *Y*-axis is the qRT-PCR analysis of the key genes involved in salt stress responses. The number below the *X*-axis is the average value of FPKMs from transcriptome data. Bars are from three biological replicates and four technical repeat experiments **(A)**, qRT-PCR analysis of jasmonic acid-related genes after treatment with 3‰ NaCl. **(B–D)** were qRT-PCR analysis of ethylene-related genes, abscisic acid-related genes and auxin-related genes, respectively.

In the RNA-seq data of *GaJAZ1*-OE plants and the WT treated by NaCl solution, a fairly large number of hormone-related genes including those for signal transduction and biosynthesis showed differential expression (FDR adjusted *P*-value < 0.05, | log2 fold-change| ≥ 1.5), including *ABA2*, *ABR1*, *CBF4*, *PP2C*, *PYL6*, *SnRK2.1*, *EFE*, *ERF1*, *ERF2*, *EIN3*, *GH3.1*, *GH3.6*, *GH3.17* and *SAURs* (Figure 6C). Compatible with these data, results of qRT-PCR analysis confirmed that some hormone-related genes reprogrammed their expression in *GaJAZ1*-OE plants compared to the WT without NaCl treatment, including *LOX3* and *AOS* (JA-related genes); *ACS*, *ERF2*, and *ERF4* (ethylene); *ABR1*, *ABA2*, *CBF4*, and *RD26* (ABA); and *GH3.6* (auxin) (Figure 7), indicating good reproducibility of transcript abundance through qRT-PCR analysis. These candidate genes indicated that the hormones played a vital role in response to salt stress.

## Discussion

The functions of JA in wound and defense responses have been thoroughly investigated in plants, and the hormone has also been demonstrated to be essential in plant growth and development (Turner et al., 2002). In this study, we evaluated the function of *GaJAZ1* in tolerance to salt stress through gene expression knockdown analysis and showed the molecular interaction of GaJAZ1 and GaMYC2 in plant salt tolerance. Moreover, ectopically overexpressing *GaJAZ1* in *G. hirsutum* plants conferred increased plant tolerance to salt stress due to reprogrammed expression of salt tolerance-related genes and promotion of root growth.

### *GaJAZ1* Participates in *G. arboreum* Plant Tolerance to Salt Stress

*GaJAZ1* expression can be induced by JA treatment, salinity, and extreme temperature stresses. Silencing of *GaJAZ1* increased the susceptibility of plants to salt stress. These results showed that *GaJAZ1* has a positive effect on salt tolerance in *G. arboretum*, possibly by repression of JA signaling. The *GaJAZ1*-silenced plants reduced the plant salinity tolerance with lower fresh weight and shorter root length compared to the control with NaCl treatment. In grapevine, priming JAZ transcription can promote the re-initiation of growth after the onset of salinity stress (Ismail et al., 2012). When *Glycine soja* JAZ2 was over-expressed in Arabidopsis, the plants performed better than WT under salinity with significant accumulation of NHX1 after 6 h (Zhu et al., 2012). In rice, *JAZs* (especially *JAZ1* and *JAZ3*) suppress *OsbHLH148* expression to promote tolerance to high-salt stresses (Dubouzet et al., 2003). JAZs can interact with RICE SALT SENSITIVE3 (RSS3), which regulates tolerance to salt (Toda et al., 2013); and over-expressing JAZ9 significantly improved plant tolerance to salt stress (Ye et al., 2009). Taken together, the accumulated data show that JAZ proteins are essential for improving salinity stress tolerance by fine-tuning JA signaling.

### *GaJAZ1* Is an Important Component in the JA Signaling Pathway

In the JA signaling pathway, JAZs are coreceptors with COI1 that recognize the JA compound to perform JA functions in plant defense and development (Turner et al., 2002; Wasternack, 2007; Kazan and Manners, 2008). Generally, JAZs can interact with MYC2 to inhibit downstream gene expression. JA promotes JAZ degradation by the 26S proteasome, which releases the inhibition of gene expression (PÉRez and Goossens, 2013; Wasternack and Hause, 2013). In this study, GaJAZ1 was verified to interact with GaMYC2, which inhibited expression of the downstream gene *GaMYB59* based on an experiment using a GUS fusion reporter system. In order to confirm the role of *GhMYB59* in the response to salt stress, we also silenced *GhMYB59* expression in WT plants. As shown in Supplementary Figure S17, *GhMYB59*-silenced plants had higher salt tolerance relative to the control, almost consistent with the results of *GaJAZ1*-OE plants. Additionally, the downstream genes’ expression levels of cotton *MYB59*, *DFL2*, *MYB13* and *CIPK9*, were investigated in *GaJAZ1*-silenced plants and *GaJAZ1*-OE plants (Supplementary Figure S18). Our data showed that *GaJAZ1* regulates the expression of *DFL2*, *MYB13* and *CIPK9* in the presence of cotton MYB59. These results further illustrated that *GaJAZ1* participates in plant salt tolerance through the JA signaling pathway.

Moreover, the *AtMYB59* gene regulated by the MYC2 TF is involved in root growth and tolerance to abiotic stress (Mu et al., 2009; Wasternack and Hause, 2013; Hickman et al., 2017; Du et al., 2019). Overexpressing *AtMYB59* plants have shorter roots compared with wild-type plants, suggesting that *AtMYB59* may inhibit root growth. However, no significant phenotypic change was observed in the aerial parts of the overexpressing *AtMYB59* lines or the mutant lines (Mu et al., 2009), which is consistent with our results that the overexpressing *GaJAZ1* did not affect aboveground phenotype of cotton plants but promoted root development possibly through repressing *GhMYB59* expression level. Hickman et al. (Hickman et al., 2017) reported that *MYB59* may function as a negative regulator to inhibit root growth through regulating *DFL2* expression. In rice, *MYB59* is reported as a negative regulator to participate in tolerance to abiotic stress (Du et al., 2019). Additionally, in the *myb48 myb59* double mutant, two salt-related genes, *MYB13* and *CIPK9*, have a reduced expression level, indicating that *MYB59* participates in plant tolerance to salt stress (Ding et al., 2013; Hickman et al., 2017; Song et al., 2018; Mohammad et al., 2019). Together, our results indicated that cotton *MYB59* is a negative regulator to participate in root growth and tolerance to salt stress.

### Ectopically Overexpressing *GaJAZ1* Increases *G. hirsutum* Plant Salt Tolerance

Given that *GaJAZ1* positively regulates plant tolerance to salt stress, we employed this gene for cotton salt tolerance breeding. Currently, upland cotton cultivars are universally planted around the world because of higher yield and quality compared to other *Gossypium* species (Zhang et al., 2015). In this study, *GaJAZ1* was ectopically expressed in upland cotton by *Agrobacterium*-mediated transformation. Overexpression of *GaJAZ1* significantly improved salt tolerance in plants grown either in the greenhouse or in the field. The measurements of physiological and biochemical parameters including antioxidant enzymes (CAT, SOD, MDA, T-AOC), chlorophyll, and the expression of salt tolerance-related genes supported that *GaJAZ1*-OE plants possess higher tolerance to salt stress compared to WT. Collectively, overexpressing *GaJAZ1* probably increases tolerance to salinity stress in upland cotton through the JA signaling pathway.

Notably, *GaJAZ1*-OE plants showed better root architectures including root length and density compared to the WT, consistent with the knockdown of *GaJAZ1* in *G. arboreum*. This result indicates that *GaJAZ1* promotes root growth and development. JA is well known to inhibit root growth, which has been exploited to screen plants with altered JA sensitivity (Srivastava et al., 2018). JAZ1 and JAZ2 are essential in root growth (Ingle et al., 2015). JAZ1 recruits HDA6 and promotes the expression of *ERF1/ORA59* to enhance resistance and root hair formation (Zhu et al., 2011). When *GaJAZ1*-OE plants were exposed to salinity stress, phenotypic and physiological traits of their root architectures were remarkably better compared to the WT, consistent with the results under normal conditions, which suggested that *GaJAZ1* promoting root growth and development can partially relieve salt damage to plants.

### Effects of Ectopically Overexpressing *GaJAZ1* on Salt Tolerance-Related Genes

Given that overexpressing *GaJAZ1* confers increased tolerance to salt stress in upland cotton, we performed RNA-seq to globally explore expression changes of salt tolerance-related genes in *GaJAZ1*-OE plants. In RNA-seq data, the expression levels of many components of the JA signaling pathway were significantly different in plants ectopically overexpressing *GaJAZ1* compared to the WT, such as *JAZ3*, *JAZ6*, *JAZ8*, *JAZ10*, *COI1*, *MYC2*, *MYC4*, and *JAR1*, which demonstrated a close relationship in expression regulation among these JA signal components (Wasternack and Song, 2017). Feedback from expression changes of JA signal components affect the JA biosynthesis pathway to regulate plant defense and growth (Wasternack and Song, 2017). In this study, JA synthesis-related genes, such as *LOXs*, *AOS*, and *AOC*, were differentially expressed between *GaJAZ1*-OE plants and the WT. These data indicated that ectopically overexpressing *GaJAZ1* in upland cotton plants affected the expression reprogramming of JA signaling and biosynthesis pathways.

The characterization of *G. arboreum* JAZ1 was addressed by loss-of-function coupled with physiological and biochemical analyses, resulting in identifying *GaJAZ1* as a positive regulator of plant salinity tolerance through the JA signaling pathway. Ectopically expressing *GaJAZ1* can confer increasing tolerance of upland cotton to salt stress, resulting from reprogrammed expression of salt tolerance-related genes and promotion of root growth. Together, this research provides a new way to utilize desirable genes from different *Gossypium* species to improve upland cotton through breeding.

## Significance Statement

Based on analyses of genetic, biochemistry, physiology, and transcriptomics, we found that *GaJAZ1* can reprogram the expression profiles of tolerance-related genes and promote the root development, resulting in higher increase of *GaJAZ1*-overexpressing plant salt tolerance compared to WT. Our study provides a new idea to explore and utilize favorable genes from *Gossypium* species for *G. hirsutum* cultivar breeding.

## Data Availability Statement

The datasets generated for this study can be found in the PRJNA439356.

## Author Contributions

GZ, XZ, FL, and JW conceived and designed the experiments. GZ, YS, QW, DL, and WQ performed the experiments. GZ, DY, WX, and ZS analyzed the data. GZ and JW wrote the manuscript. ZY and XG revised the manuscript. All authors read and approved the final manuscript.

## Conflict of Interest

The authors declare that the research was conducted in the absence of any commercial or financial relationships that could be construed as a potential conflict of interest.

## Footnotes

1 http://www.isaaa.org/

2 https://plantscience.psu.edu/research/labs/roots/images/winrhizo/view

3 http://www.genome.jp/kegg/
