# Supplementary material for: Gossypium hirsutum Salt Tolerance Is Enhanced by Overexpression of G. arboreum JAZ1
Source: Front Bioeng Biotechnol. 2020 Mar 10;8:157. doi: 10.3389/fbioe.2020.00157 (PMC7076078; doi:10.3389/fbioe.2020.00157)
Supplement: Supplementary file 1 [file Data_Sheet_1.pdf]

Fig S1

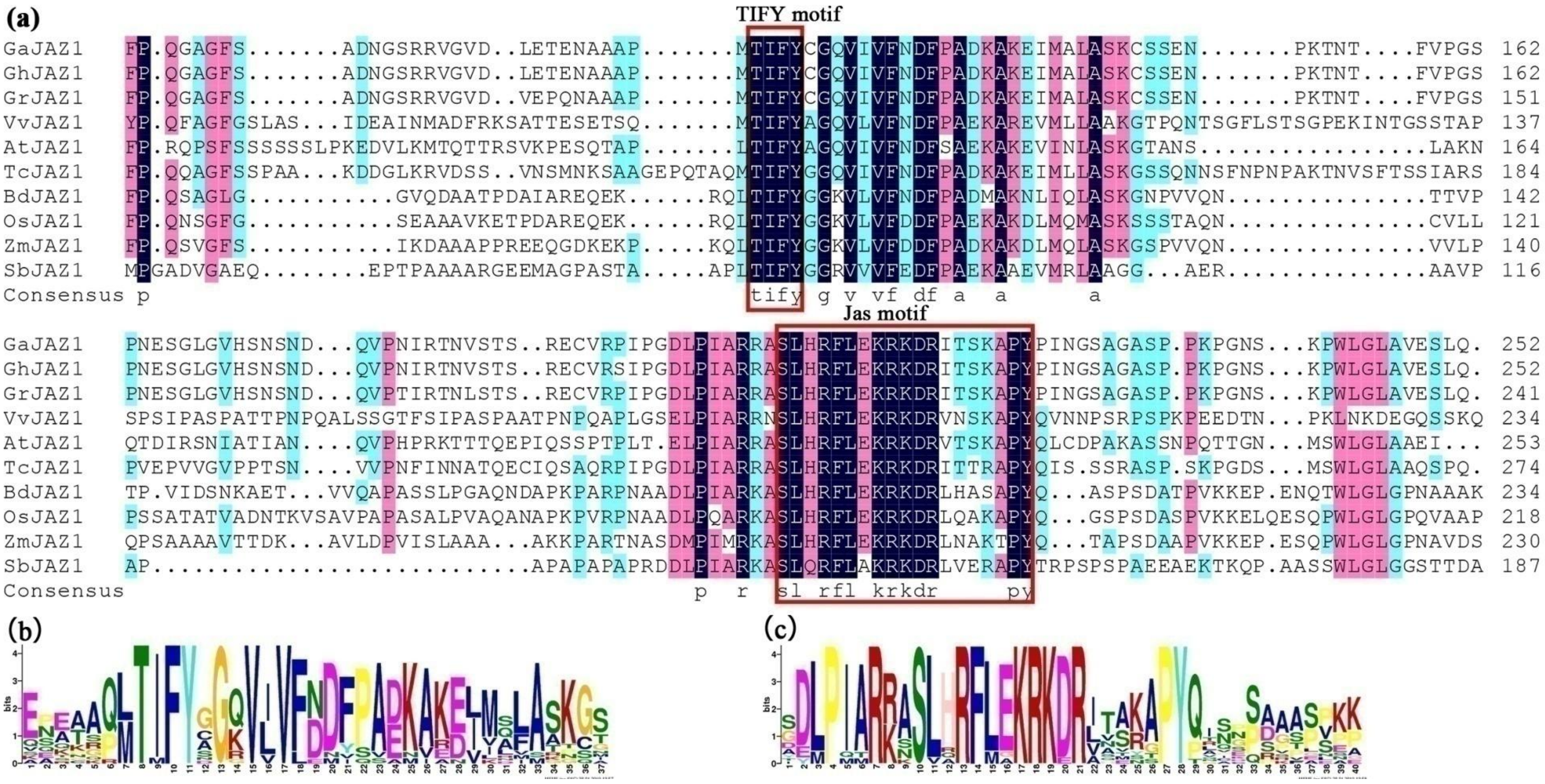
(a)
TIFY motif
GaJAZ1 FP.QGAGFS.......ADNGSRRVGVD..LETENAAAP.......MTIFYCGQVIVFNDFPADKAKEIMALASKCSSEN.......PKTNT....FVPGS 162
GhJAZ1 FP.QGAGFS.......ADNGSRRVGVD..LETENAAAP.......MTIFYCGQVIVFNDFPADKAKEIMALASKCSSEN.......PKTNT....FVPGS 162
GrJAZ1 FP.QGAGFS.......ADNGSRRVGVD..VEPQNAAAP.......MTIFYCGQVIVFNDFPADKAKEIMALASKCSSEN.......PKTNT....FVPGS 151
VvJAZ1 YP.QFAGFGSLAS...IDEAINMADFRKSATTESETSQ.......MTIFYAGQVLVFNDFPAEKAREVMLLAAKGTPQNTSGFLSTSGPEKINTGSSTAP 137
AtJAZ1 FP.RQPSFSSSSSSLPKEDVLKMTQTTRSVKPESQTAP.......LTIFYAGQVIVFNDFSAEKAKEVINLASKGTANS...............LAKN 164
TcJAZ1 FP.QQAGFSSPAA...KDDGLKRVDSS..VNSMNKSAAGEPQTAQMTIFYGGQVIVFNDFPADKAKEIMLLASKGSSQNNSFNPNPAKTNVSFTSSIARS 184
BdJAZ1 FP.QSAGLG..........GVQDAATPDAIAREQEK......RQLTIFYGGKVLVFNDFPADMAKNLIQLASKGNPVVQN..............TTVP 142
OsJAZ1 FP.QNSGFG..........SEAAAVKETPDAREQEK......RQLTIFYGGKVLVFDDFPAEKAKDLMQMASKSSSTAQN..............CVLL 121
ZmJAZ1 FP.QSVGFS..........IKDAAAPPREEQGDKEKP.....KQLTIFYGGKVLVFDDFPADKAKDLMQLASKGSPVVQN..............VVLP 140
SbJAZ1 MPGADVGAEQ.......EPTPAAAARGEEMAGPASTA.....APLTIFYGGRVVVFEDFPAEKAAEVMRLAAGG...AER...............AAVP 116
Consensus p tify g v vf df a a a
Jas motif
GaJAZ1 PNESGLGVHSNSND...QVPNIRTNVSTS..RECVRPIPGDLPIARRASLHRFLEKRKDRITSKAPYPINGSAGASP.PKPGNS...KPWLGLAVESLQ. 252
GhJAZ1 PNESGLGVHSNSND...QVPNIRTNVSTS..RECVRSIPGDLPIARRASLHRFLEKRKDRITSKAPYPINGSAGASP.PKPGNS...KPWLGLAVESLQ. 252
GrJAZ1 PNESGLGVHSNSND...QVPTIRTNLSTS..RECVRPIPGDLPIARRASLHRFLEKRKDRITSKAPYPINGSAGASP.PKPGNS...KPWLGLAVESLQ. 241
VvJAZ1 SPSIPASPATTPNPQALSSGTFSIPASPAATPNPQAPLGSELPIARRNSLHRFLEKRKDRVNSKAPYQVNNPSRPSPKPEEDTN...PKLNKDEGQSSKQ 234
AtJAZ1 QTDIRSNIATIAN....QVPHPRKTTTQEPIQSSPTPLT.ELPIARRASLHRFLEKRKDRVTSKAPYQLCDPAKASSNPQTTGN...MSWLGLAAEI... 253
TcJAZ1 PVEPVVGVPPTSN....VVPNFINNATQECIQSAQRPIPGDLPIARRASLHRFLEKRKDRITTRAPYQIS.SSRASP.SKPGDS...MSWLGLAAQSPQ. 274
BdJAZ1 TP.VIDSNKAET...VVQAPASSLPGAQNDAPKPARPNAADLPIARKASLHRFLEKRKDRLHASAPYQ...ASPSDATPVKKEP.ENQTWLGLGPNAAAK 234
OsJAZ1 PSSATATVADNTKVSAVPAPASALPVAQANAPKPVRPNAADLPQARKASLHRFLEKRKDRLQAKAPYQ...GSPSDASPVKKELQESQPWLGLGPQVAAP 218
ZmJAZ1 QPSAAAAVTTDK...AVLDPVISLAAA...AKKPARTNASDMPIMRKASLHRFLEKRKDRLNAKTPYQ...TAPSDAAPVKKEP.ESQPWLGLGPNAVDS 230
SbJAZ1 AP...........................APAPAPAPRDDLPIARKASLQRFLAKRKDRLVERAPYTRPSPSPAEEAEKTKQP.AASSWLGLGGSTTDA 187
Consensus p r sl rfl krkdr py
(b)
(c)
bits

Fig S2

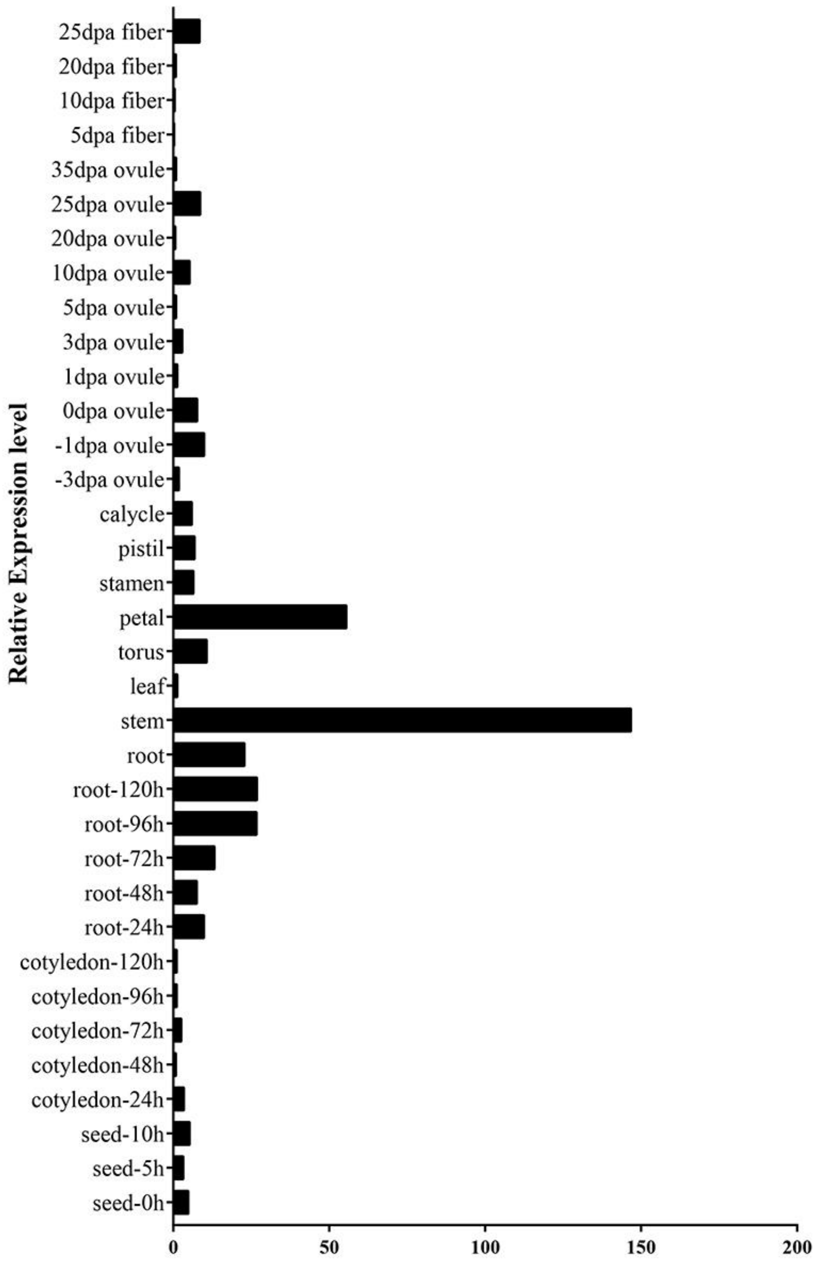

Relative Expression level
25dpa fiber
20dpa fiber
10dpa fiber
5dpa fiber
35dpa ovule
25dpa ovule
20dpa ovule
10dpa ovule
5dpa ovule
3dpa ovule
1dpa ovule
0dpa ovule
-1dpa ovule
-3dpa ovule
calycle
pistil
stamen
petal
torus
leaf
stem
root
root-120h
root-96h
root-72h
root-48h
root-24h
cotyledon-120h
cotyledon-96h
cotyledon-72h
cotyledon-48h
cotyledon-24h
seed-10h
seed-5h
seed-0h
0
50
100
150
200

Fig S3

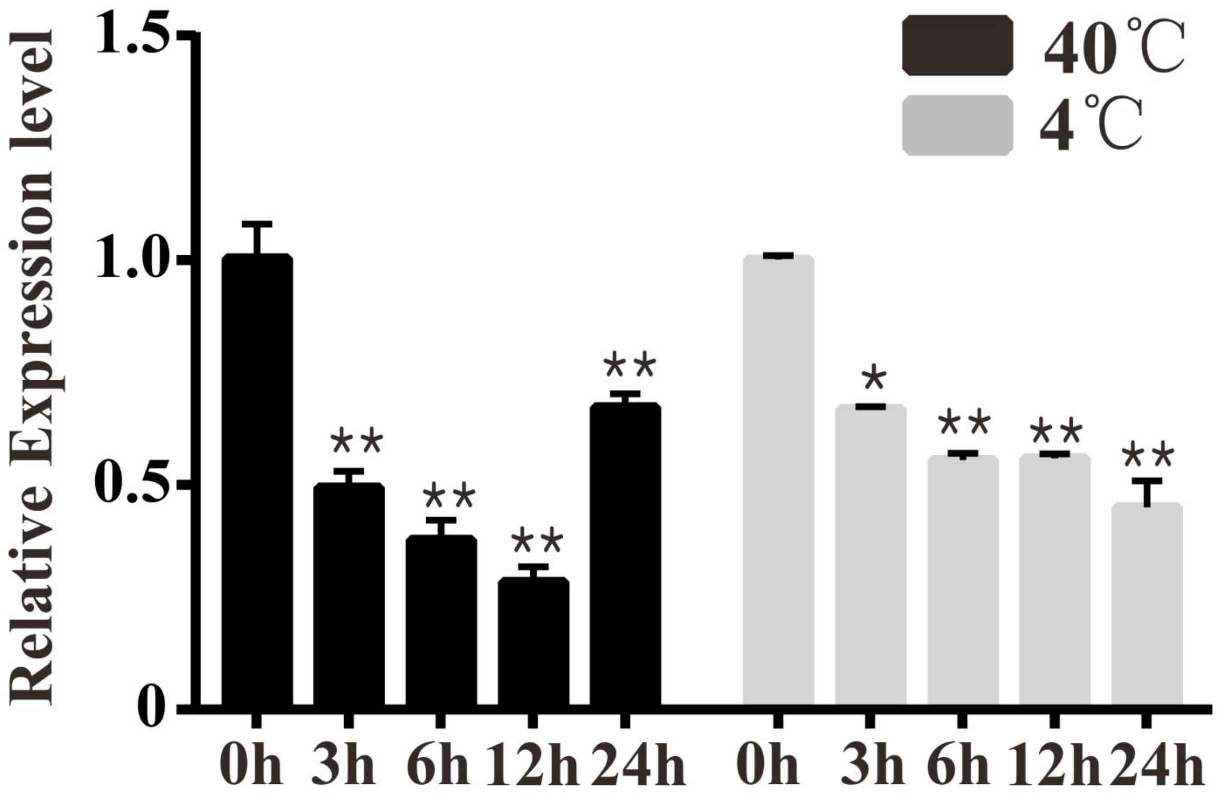
Relative Expression level
1.5
1.0
0.5
0
40℃
4℃
**
**
**
**
*
**
**
**
0h
3h
6h
12h
24h
0h
3h
6h
12h
24h

Fig S4

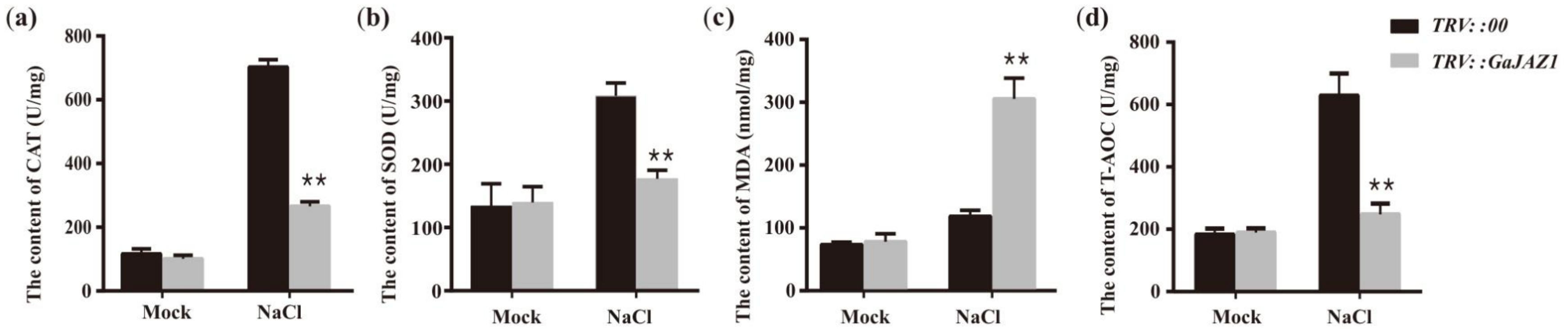

(a)
The content of CAT (U/mg)
0
200
400
600
800
Mock
NaCl
**
(b)
The content of SOD (U/mg)
0
100
200
300
400
Mock
NaCl
**
(c)
The content of MDA (nmol/mg)
0
100
200
300
400
Mock
NaCl
**
(d)
The content of T-AOC (U/mg)
0
200
400
600
800
Mock
NaCl
**
TRV: :00
TRV: :GaJAZ1

Fig S5

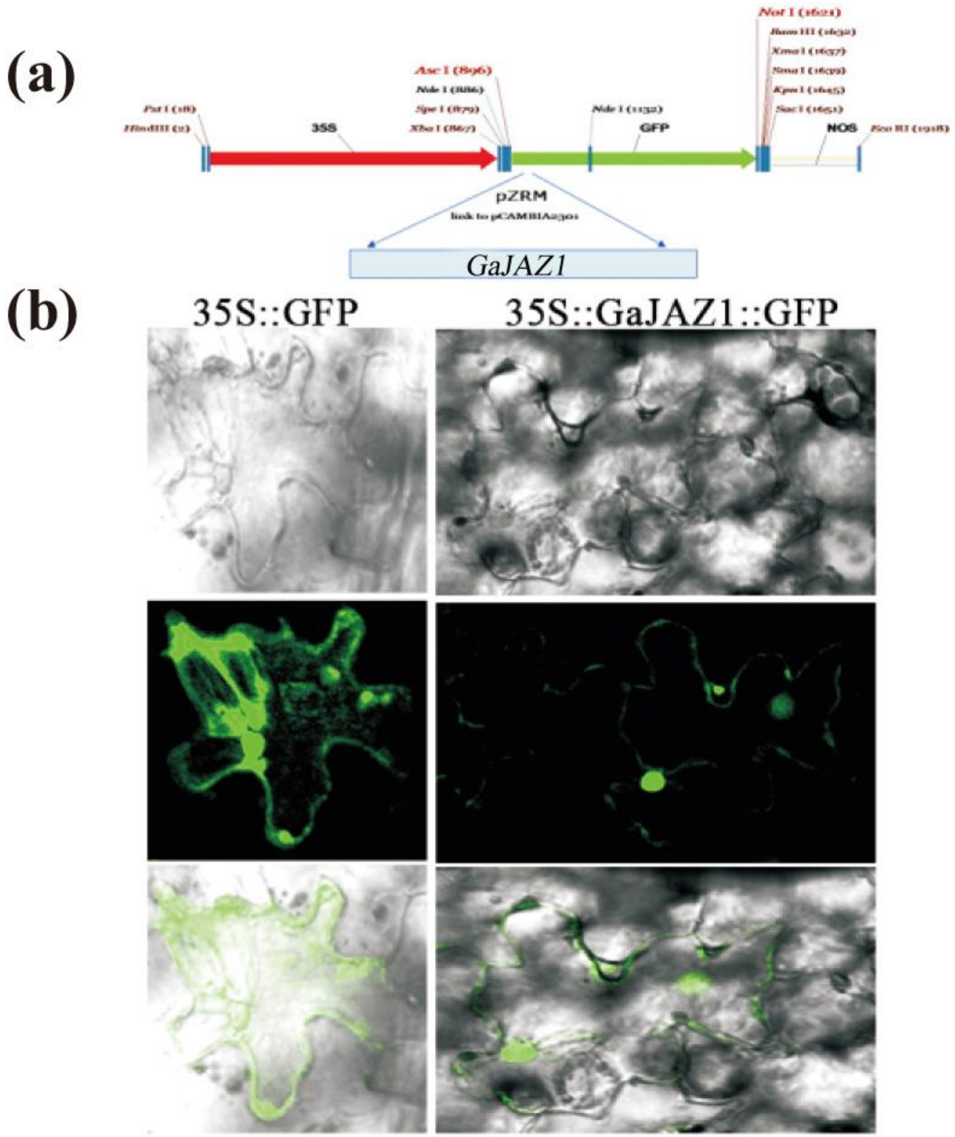
(a)
Not I (1621)
Bam HI (1632)
Xma I (1637)
Sma I (1639)
Kpn I (1645)
Sac I (1651)
Asc I (896)
Nde I (886)
Spe I (879)
Xba I (867)
Pst I (18)
HindIII (2)
35S
Nde I (1132)
GFP
NOS
Eco RI (1918)
pZRM
link to pCAMBIA2301
GaJAZ1
(b)
35S::GFP
35S::GaJAZ1::GFP

Fig S6

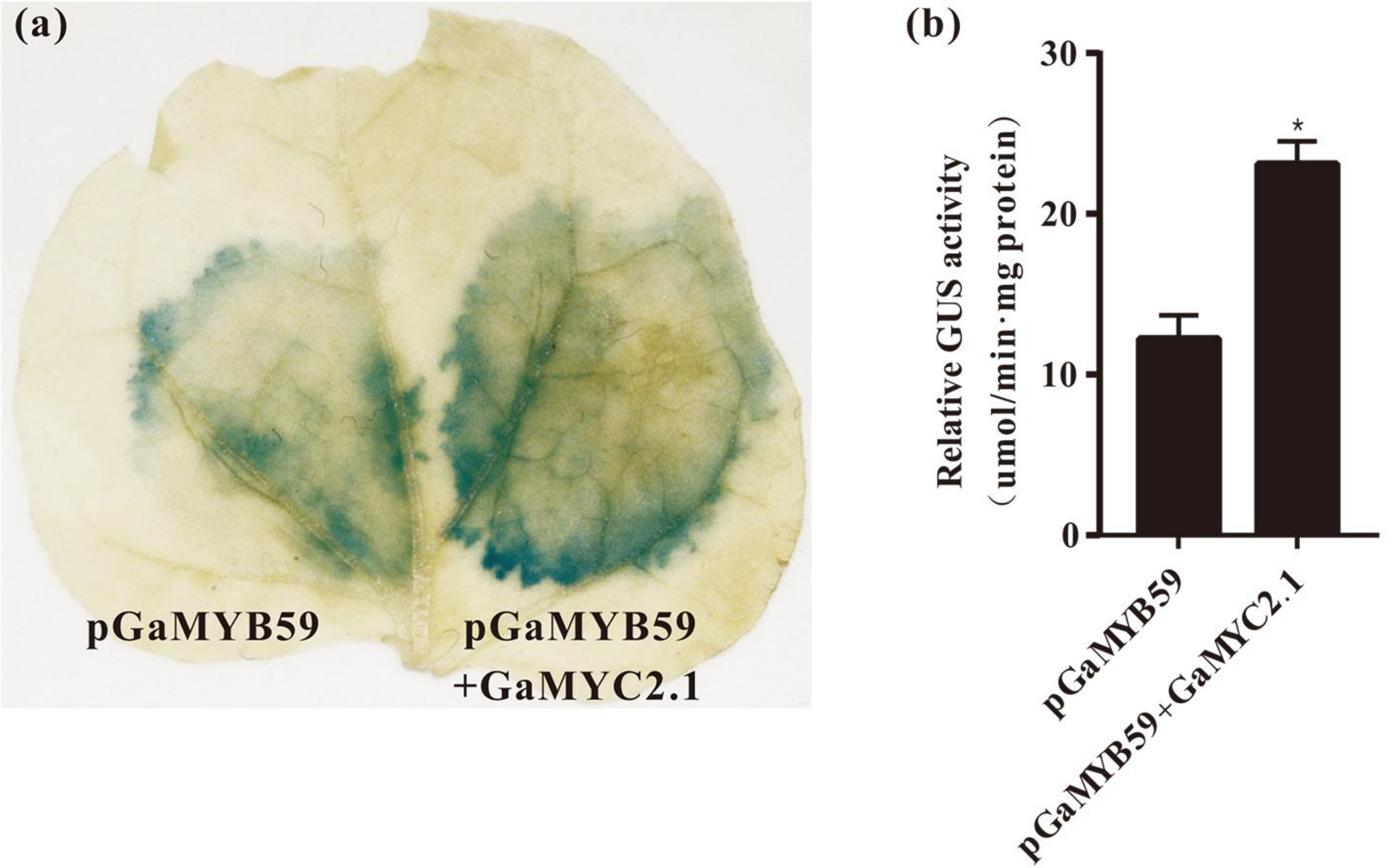
(a)
pGaMYB59
pGaMYB59
+GaMYC2.1
(b)
Relative GUS activity
(umol/min·mg protein)
30
20
10
0
*
pGaMYB59
pGaMYB59+GaMYC2.1

Fig S7

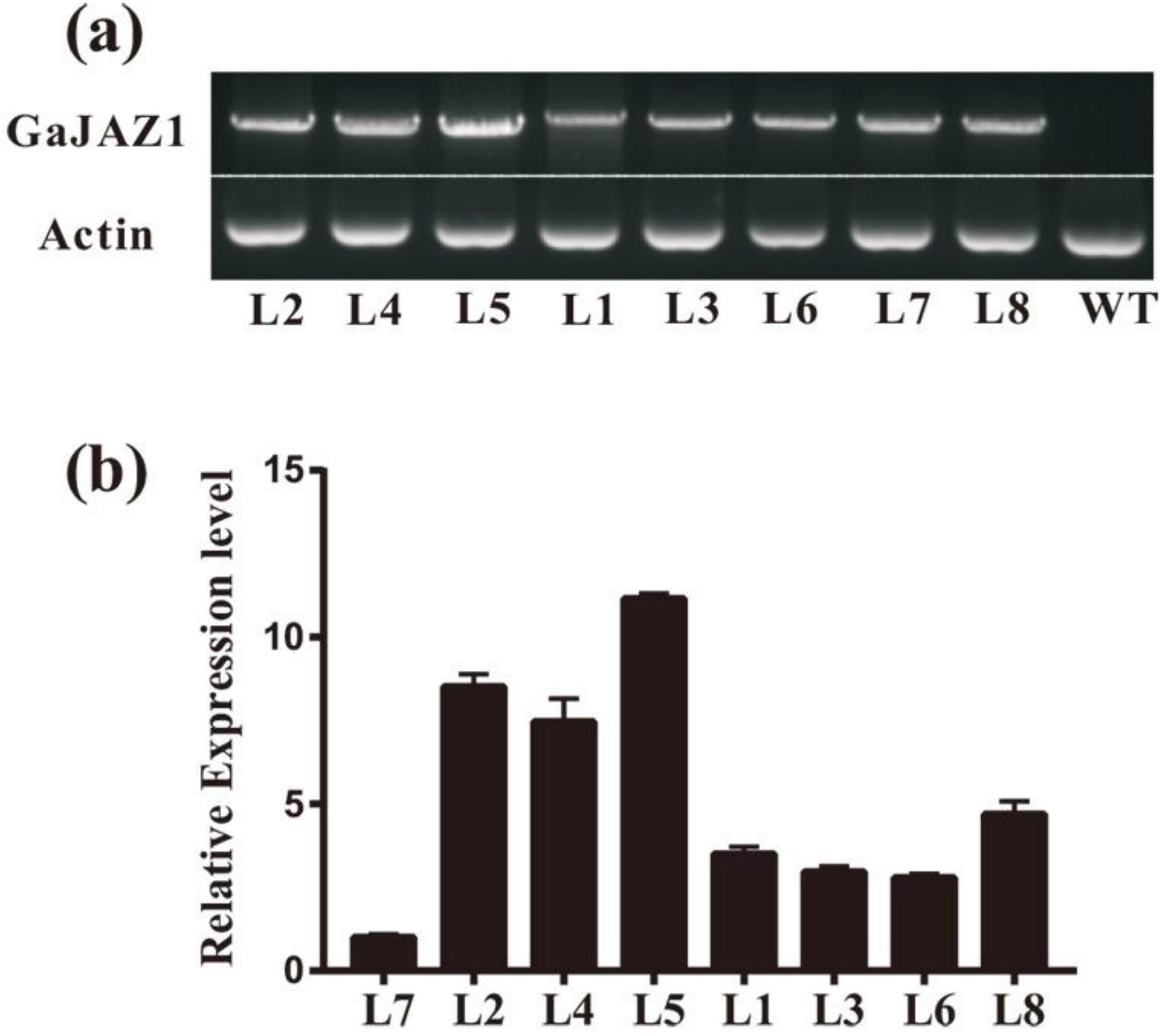
(a)
GaJAZ1
Actin
L2
L4
L5
L1
L3
L6
L7
L8
WT
(b)
Relative Expression level
15
10
5
0
L7
L2
L4
L5
L1
L3
L6
L8

Fig S8

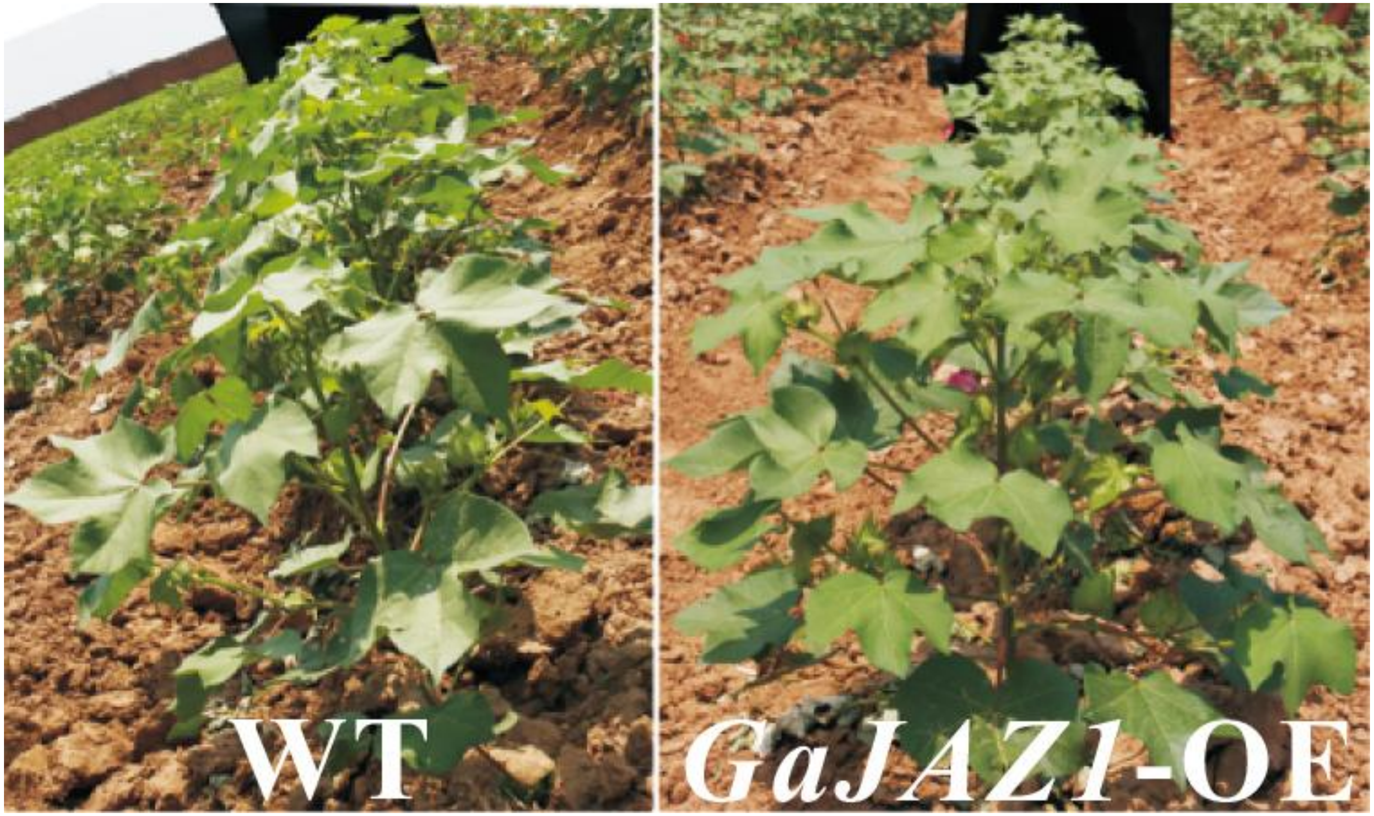

Fig S9

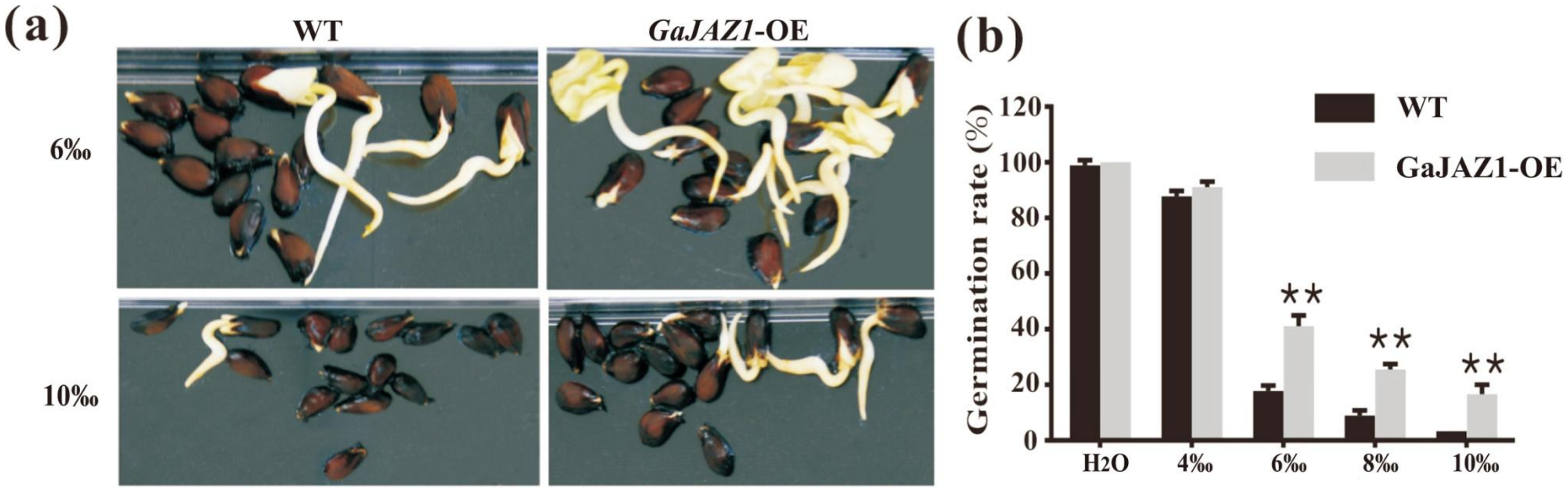
(a)
WT
GaJAZ1-OE
6‰
10‰
(b)
Germination rate (%)
120
100
80
60
40
20
0
WT
GaJAZ1-OE
**
**
**
H2O
4‰
6‰
8‰
10‰

Fig S10

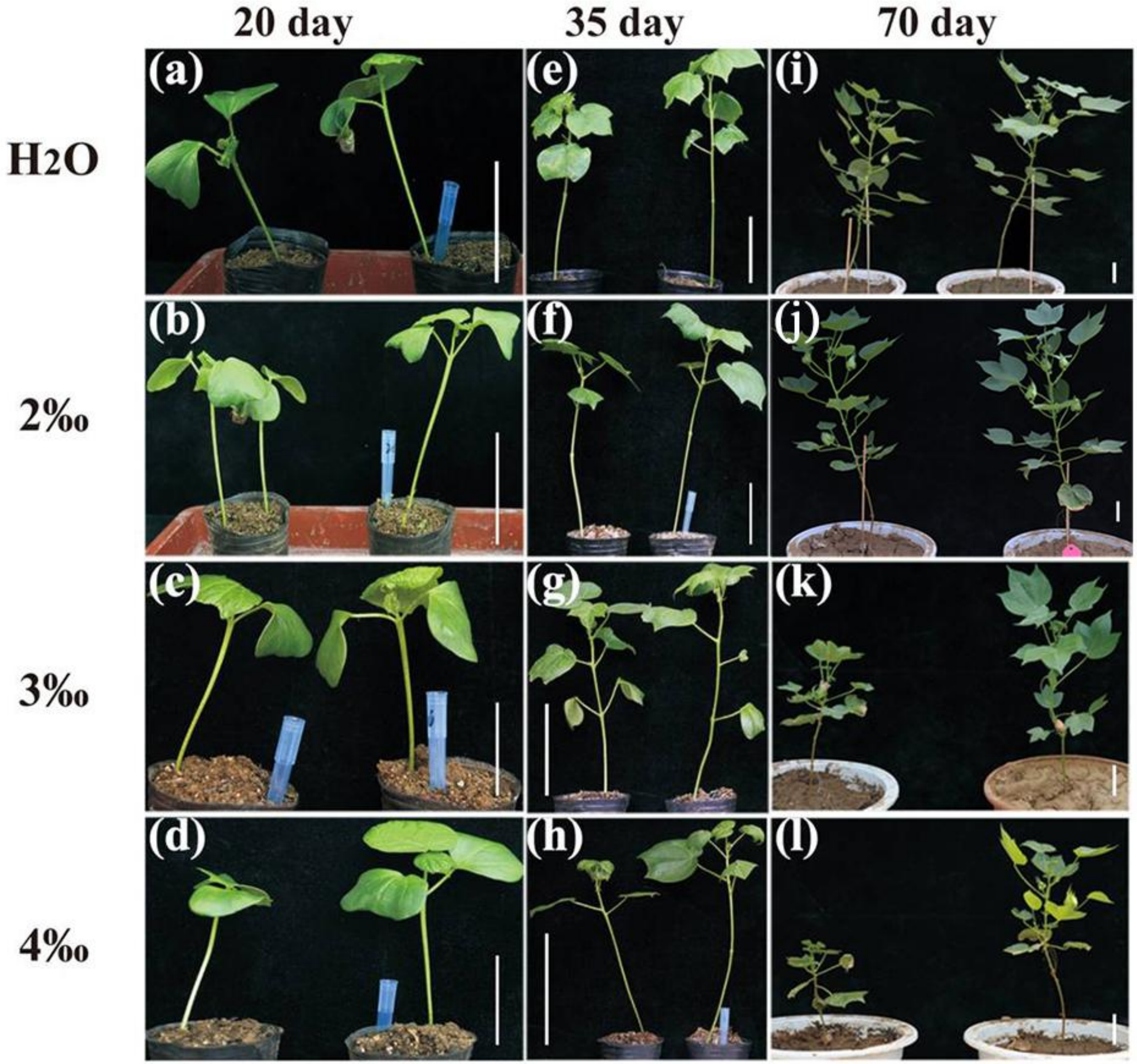

Fig S11

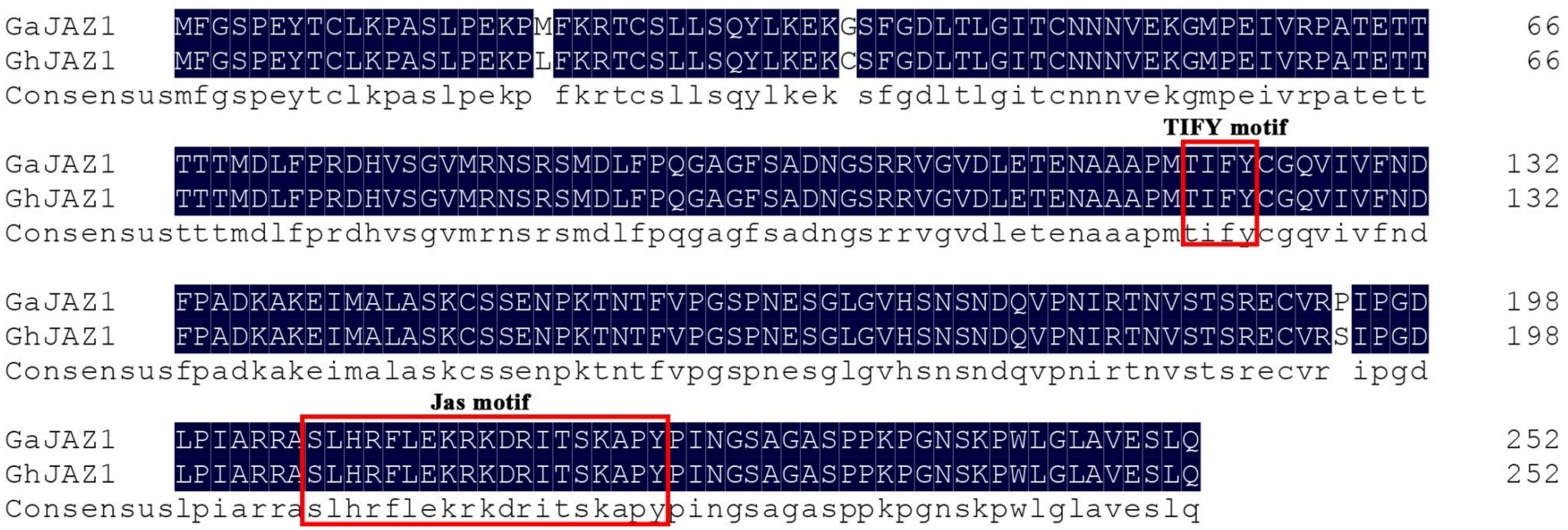
GaJAZ1 MFGSPEYTCLKPASLPEKPMFKRTCSLLSQYLKEKGSFGDLTLGITCNNNVEKGMPEIVRPATETT 66
GhJAZ1 MFGSPEYTCLKPASLPEKPLFKRTCSLLSQYLKEKCSFGDLTLGITCNNNVEKGMPEIVRPATETT 66
Consensus mfgspeytclkpaslpekp fkrtcsllsqylkek sfgdltlgitcnnnvekgmpeivrpatett
TIFY motif
GaJAZ1 TTTMDLFPRDHVSGVMRNSRSMDLFPQGAGFSADNGSRRVGVDLETENAAAPMTIFYCGQVIVFND 132
GhJAZ1 TTTMDLFPRDHVSGVMRNSRSMDLFPQGAGFSADNGSRRVGVDLETENAAAPMTIFYCGQVIVFND 132
Consensus tttmdlfprdhvsgvmrnsrsmdlfpqgagfsadngsrrvgvdletenaaapmtifycgqvivfnd
GaJAZ1 FPADKAKEIMALASKCSSENPKTNTFVPGSPNESGLGVHSNSNDQVPNIRTNVSTSRECVRPIPGD 198
GhJAZ1 FPADKAKEIMALASKCSSENPKTNTFVPGSPNESGLGVHSNSNDQVPNIRTNVSTSRECVRSIPGD 198
Consensus fpadkakeimalaskcssenpktntfvpgspnesglgvhsnsndqvpnirtnvstsrecvr ipgd
Jas motif
GaJAZ1 LPIARRASLHRFLEKRKDRITSKAPYPINGSAGASPPKPGNSKPWLGLAVESLQ 252
GhJAZ1 LPIARRASLHRFLEKRKDRITSKAPYPINGSAGASPPKPGNSKPWLGLAVESLQ 252
Consensus lpiarraslhrflekrkdritskapypingsagasppkpgnskpwlglaveslq

Fig S12

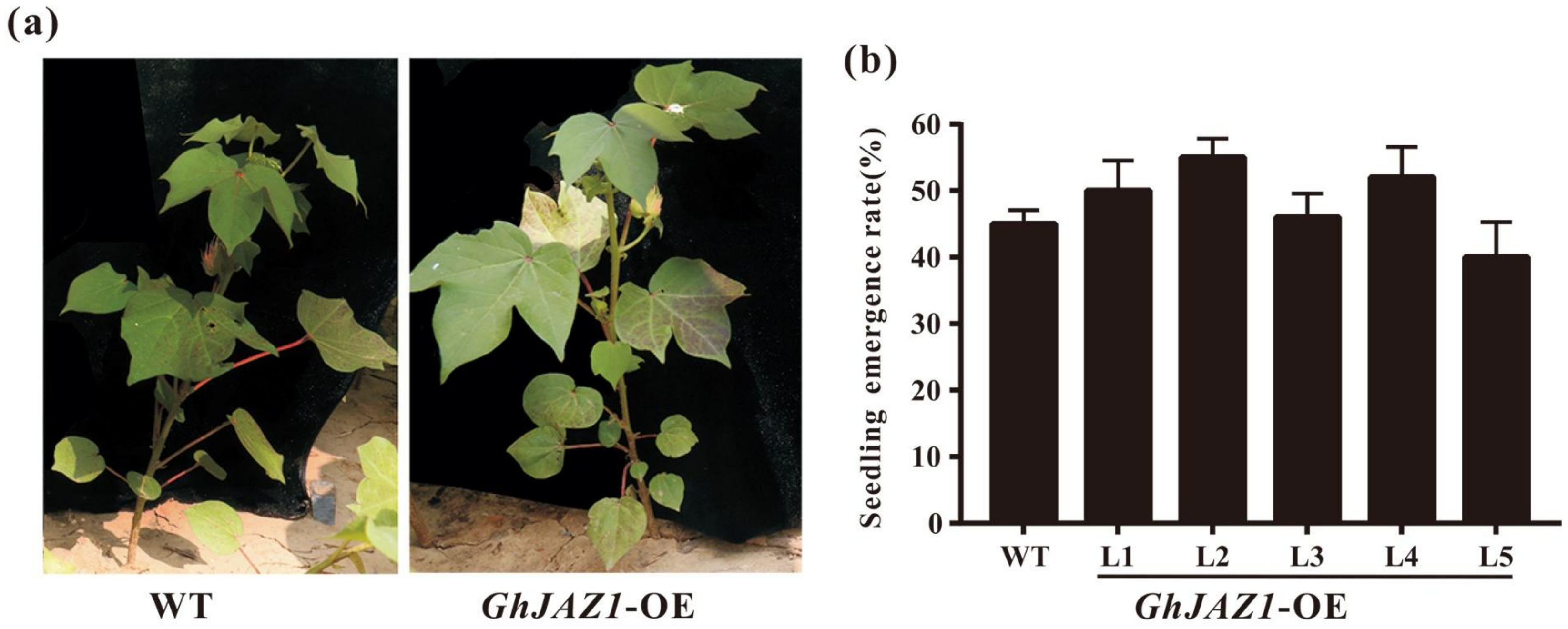
(a)
WT
GhJAZ1-OE
(b)
Seedling emergence rate(%)
60
50
40
30
20
10
0
WT
L1
L2
L3
L4
L5
GhJAZ1-OE

Fig S13

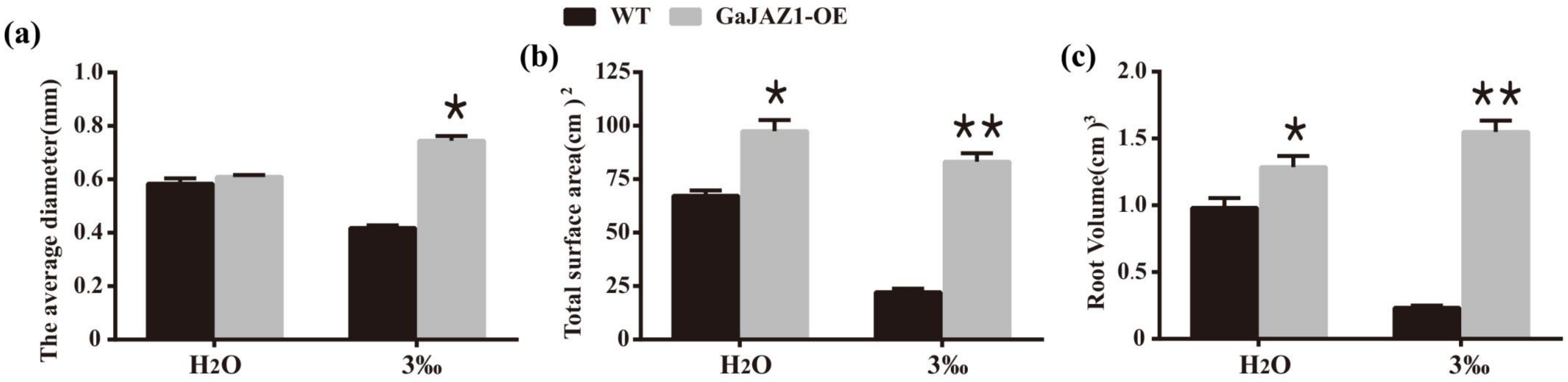
WT
GaJAZ1-OE
(a)
The average diameter(mm)
1.0
0.8
0.6
0.4
0.2
0
H2O
3‰
(b)
Total surface area(cm)2
125
100
75
50
25
0
H2O
3‰
(c)
Root Volume(cm)3
2.0
1.5
1.0
0.5
0
H2O
3‰

Fig S14

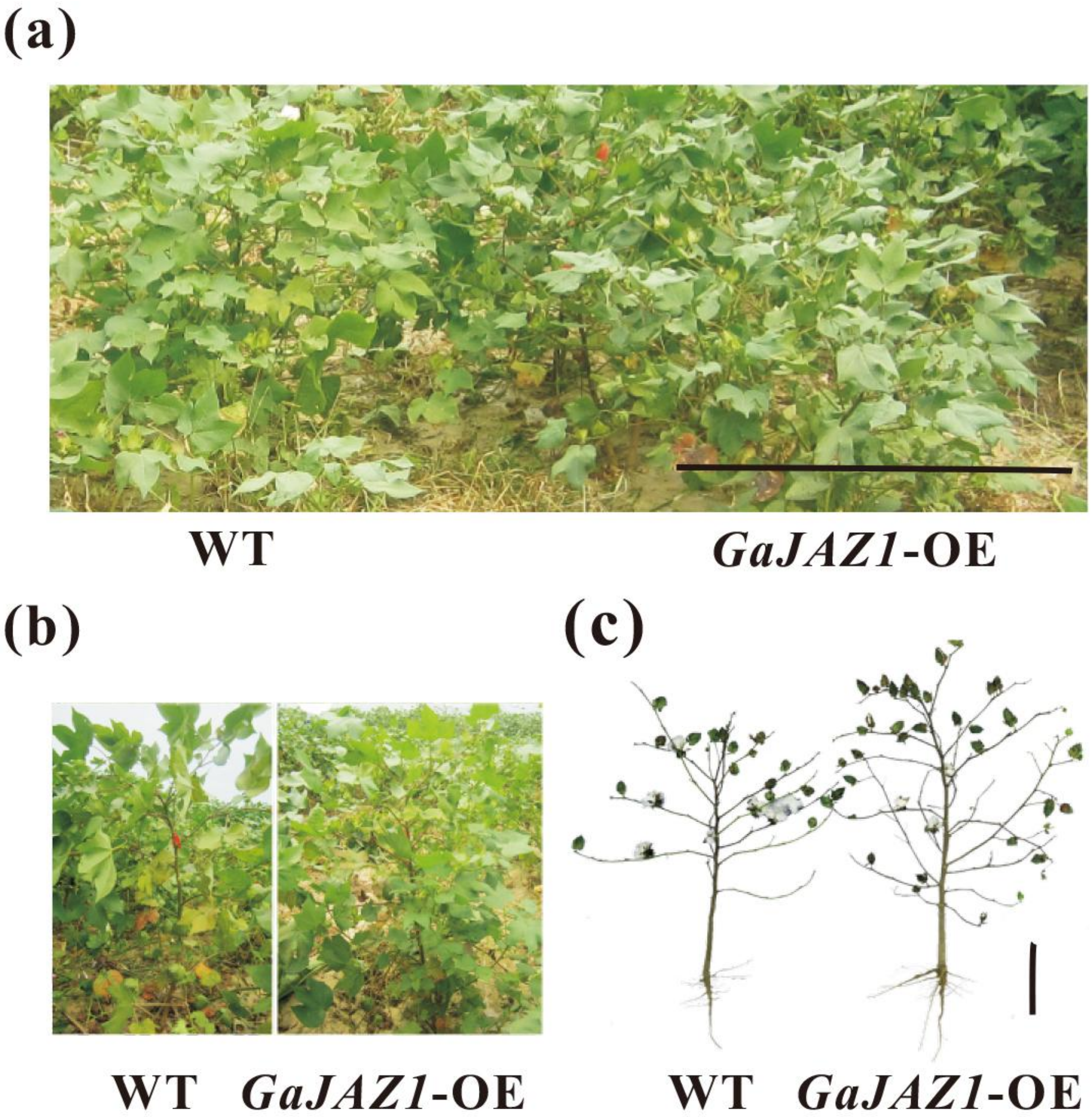

Fig S15

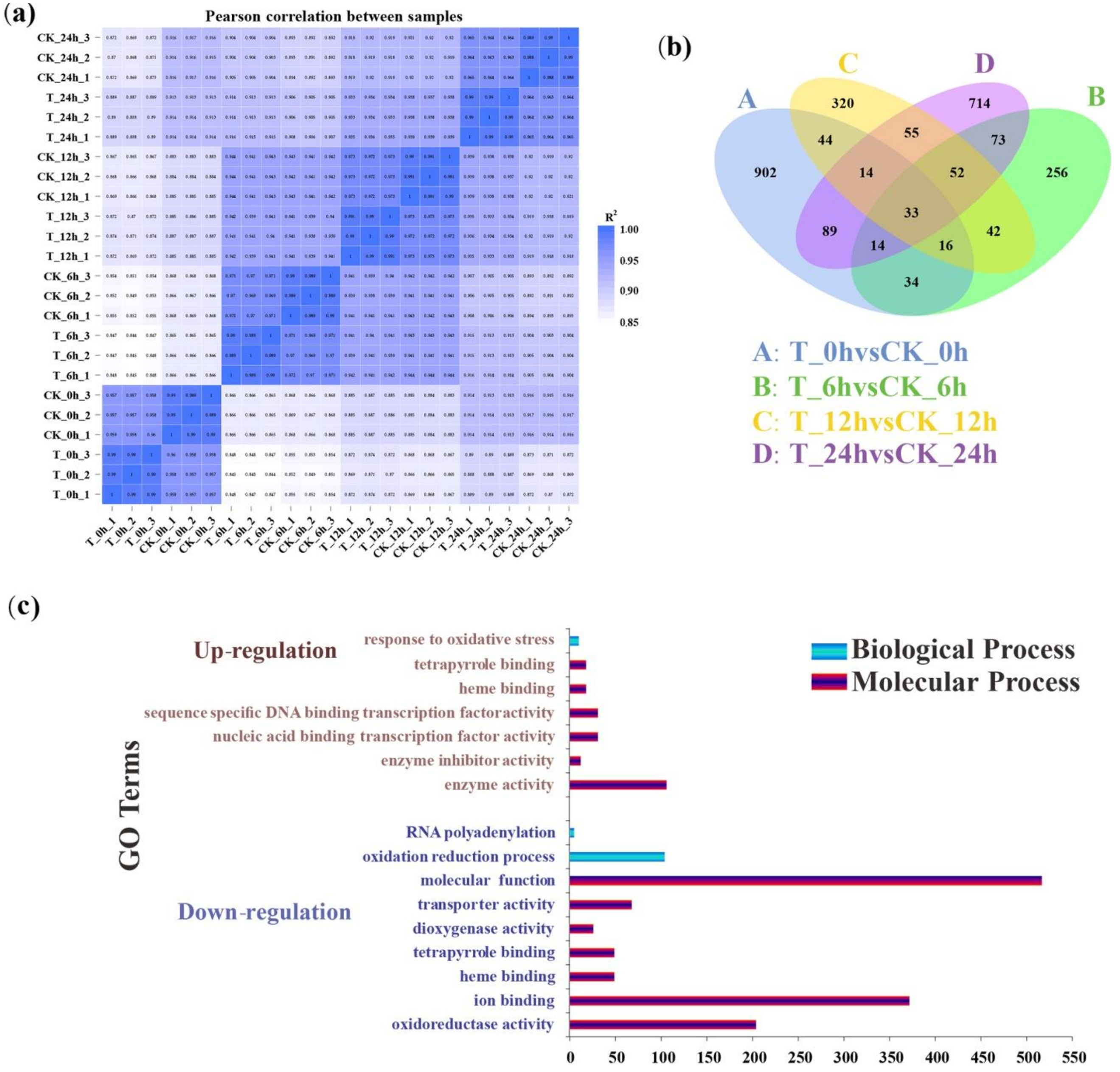
(a)
Pearson correlation between samples
CK_24h_3
CK_24h_2
CK_24h_1
T_24h_3
T_24h_2
T_24h_1
CK_12h_3
CK_12h_2
CK_12h_1
T_12h_3
T_12h_2
T_12h_1
CK_6h_3
CK_6h_2
CK_6h_1
T_6h_3
T_6h_2
T_6h_1
CK_0h_3
CK_0h_2
CK_0h_1
T_0h_3
T_0h_2
T_0h_1
T_0h_1
T_0h_2
T_0h_3
CK_0h_1
CK_0h_2
CK_0h_3
T_6h_1
T_6h_2
T_6h_3
CK_6h_1
CK_6h_2
CK_6h_3
T_12h_1
T_12h_2
T_12h_3
CK_12h_1
CK_12h_2
CK_12h_3
T_24h_1
T_24h_2
T_24h_3
CK_24h_1
CK_24h_2
CK_24h_3
R^2
1.00
0.95
0.90
0.85
(b)
A
C
D
B
320
714
44
55
73
902
14
52
256
33
89
42
14
16
34
A: T_0hvsCK_0h
B: T_6hvsCK_6h
C: T_12hvsCK_12h
D: T_24hvsCK_24h
(c)
Up-regulation
response to oxidative stress
tetrapyrrole binding
heme binding
sequence specific DNA binding transcription factoractivity
nucleic acid binding transcription factor activity
enzyme inhibitor activity
enzyme activity
GO Terms
RNA polyadenylation
oxidation reduction process
molecular function
Down-regulation
transporter activity
dioxygenase activity
tetrapyrrole binding
heme binding
ion binding
oxidoreductase activity
Biological Process
Molecular Process
0
50
100
150
200
250
300
350
400
450
500
550

Fig S16

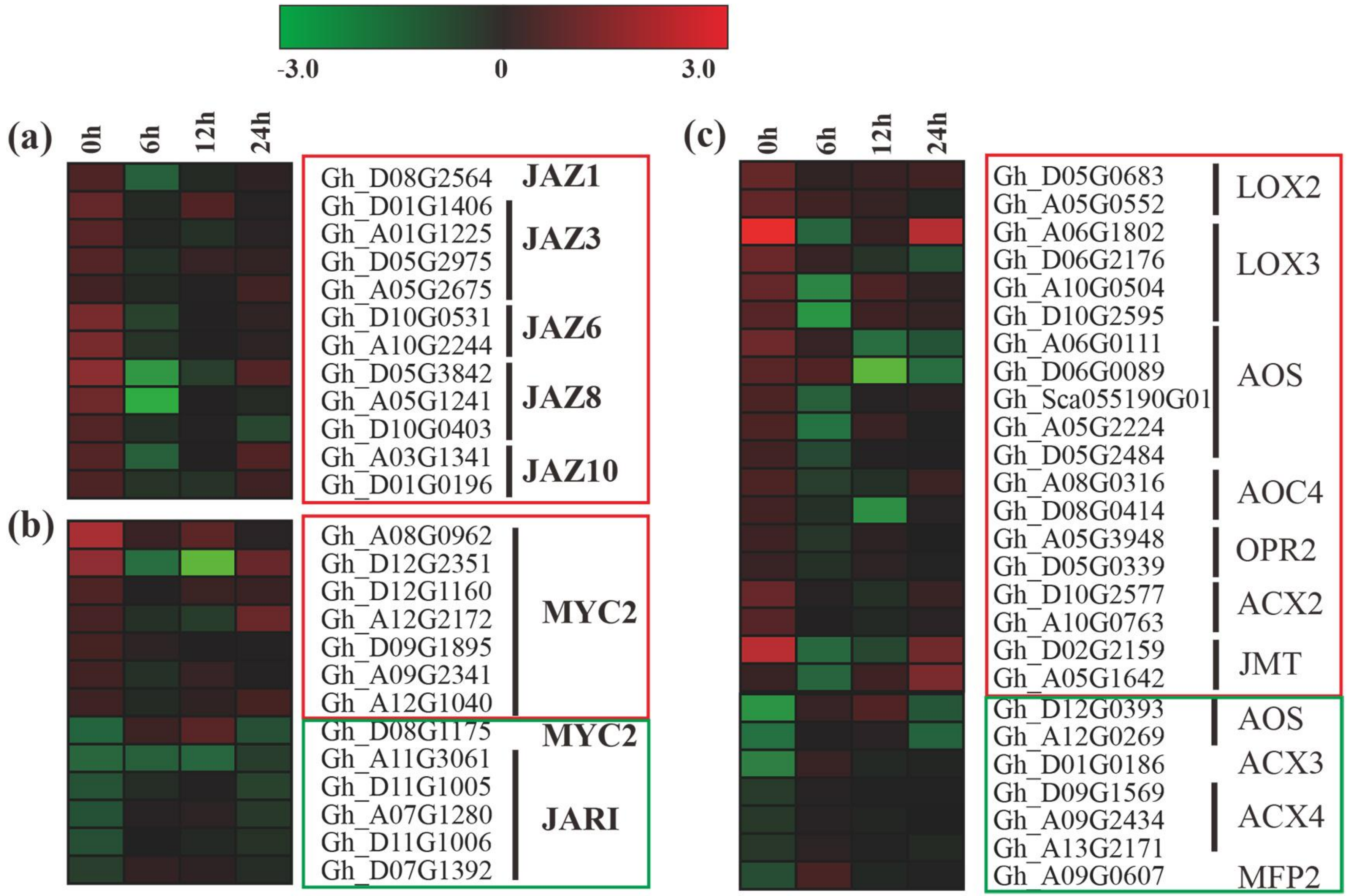

-3.0
0
3.0
(a)
0h
6h
12h
24h
Gh_D08G2564
Gh_D01G1406
Gh_A01G1225
Gh_D05G2975
Gh_A05G2675
Gh_D10G0531
Gh_A10G2244
Gh_D05G3842
Gh_A05G1241
Gh_D10G0403
Gh_A03G1341
Gh_D01G0196
JAZ1
JAZ3
JAZ6
JAZ8
JAZ10
(b)
Gh_A08G0962
Gh_D12G2351
Gh_D12G1160
Gh_A12G2172
Gh_D09G1895
Gh_A09G2341
Gh_A12G1040
Gh_D08G1175
Gh_A11G3061
Gh_D11G1005
Gh_A07G1280
Gh_D11G1006
Gh_D07G1392
MYC2
MYC2
JARI
(c)
0h
6h
12h
24h
Gh_D05G0683
Gh_A05G0552
Gh_A06G1802
Gh_D06G2176
Gh_A10G0504
Gh_D10G2595
Gh_A06G0111
Gh_D06G0089
Gh_Sca055190G01
Gh_A05G2224
Gh_D05G2484
Gh_A08G0316
Gh_D08G0414
Gh_A05G3948
Gh_D05G0339
Gh_D10G2577
Gh_A10G0763
Gh_D02G2159
Gh_A05G1642
Gh_D12G0393
Gh_A12G0269
Gh_D01G0186
Gh_D09G1569
Gh_A09G2434
Gh_A13G2171
Gh_A09G0607
LOX2
LOX3
AOS
AOC4
OPR2
ACX2
JMT
AOS
ACX3
ACX4
MFP2

Fig S17

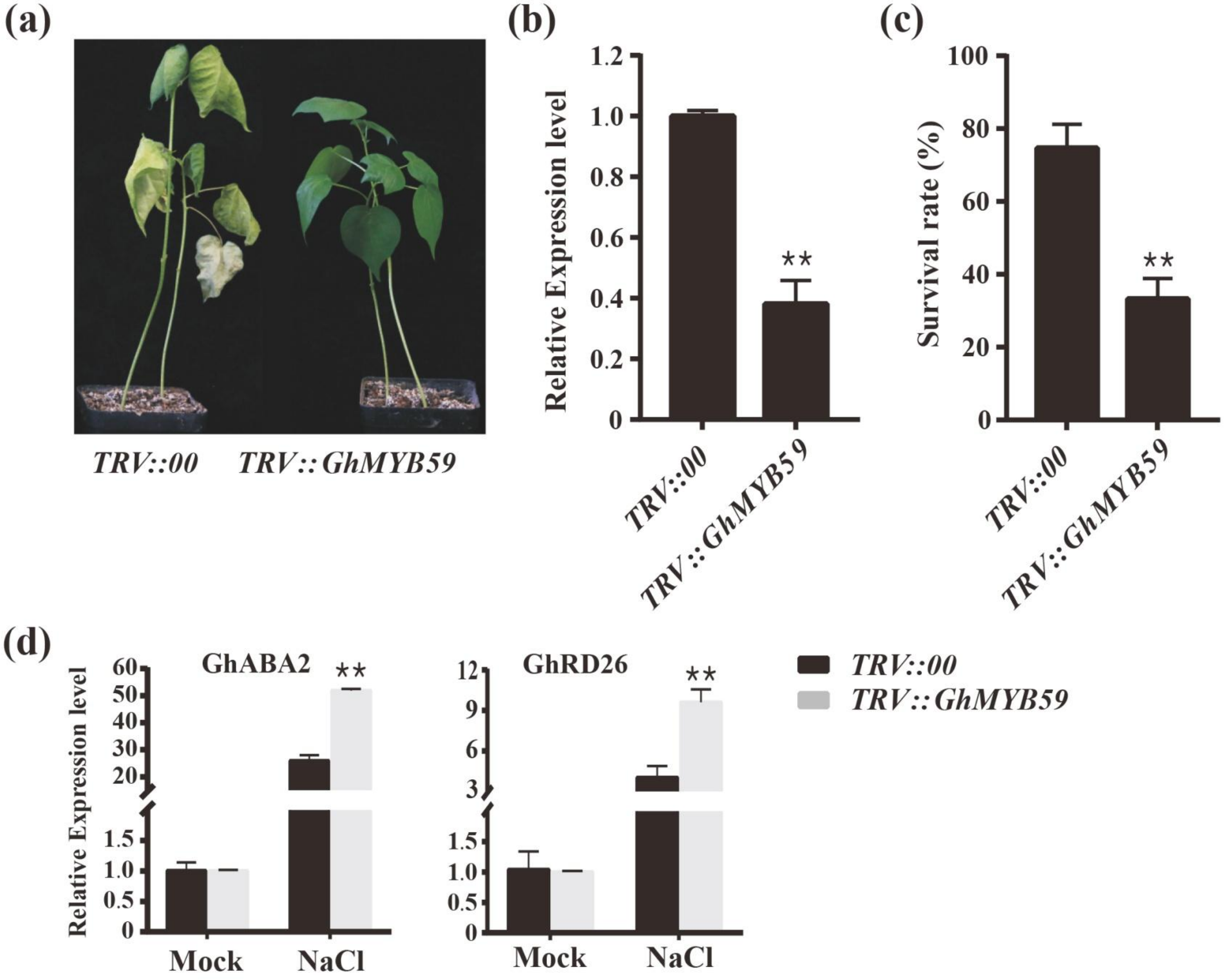

(a)
TRV::00
TRV::GhMYB59
(b)
Relative Expression level
1.2
1.0
0.8
0.6
0.4
0.2
0
**
TRV::00
TRV::GhMYB59
(c)
Survival rate (%)
100
80
60
40
20
0
**
TRV::00
TRV::GhMYB59
(d)
Relative Expression level
GhABA2
60
50
40
30
20
1.5
1.0
0.5
0
**
Mock
NaCl
GhRD26
12
9
6
3
1.5
1.0
0.5
0
**
Mock
NaCl
TRV::00
TRV::GhMYB59

Fig S18

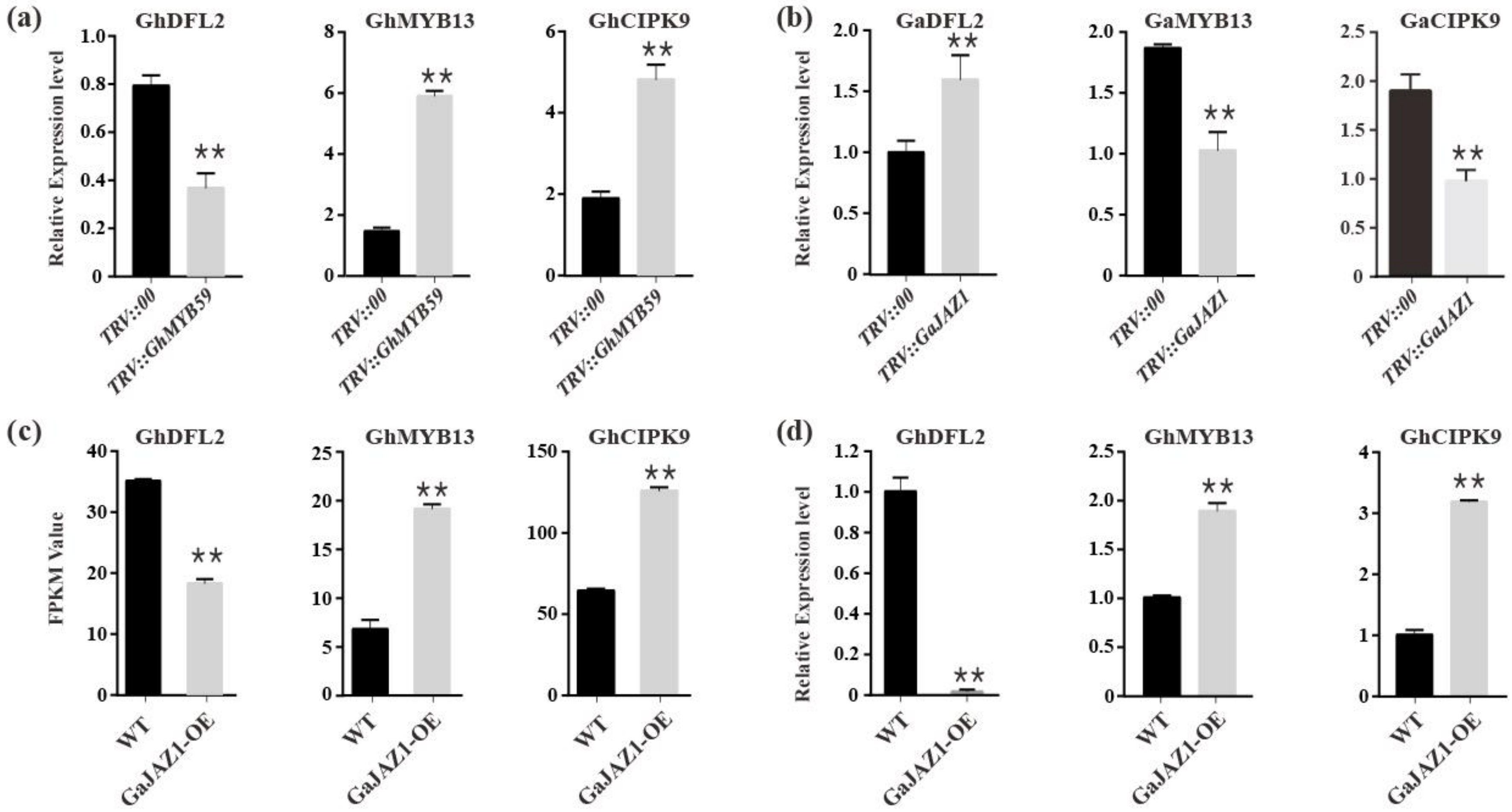

(a)
GhDFL2
GhMYB13
GhCIPK9
Relative Expression level
TRV::00
TRV::GhMYB59
**
(b)
GaDFL2
GaMYB13
GaCIPK9
Relative Expression level
TRV::00
TRV::GaJAZ1
**
(c)
GhDFL2
GhMYB13
GhCIPK9
FPKM Value
WT
GaJAZ1-OE
**
(d)
GhDFL2
GhMYB13
GhCIPK9
Relative Expression level
WT
GaJAZ1-OE
**
