# Supplementary material for: Gossypium hirsutum Salt Tolerance Is Enhanced by Overexpression of G. arboreum JAZ1
Source: Front Bioeng Biotechnol. 2020 Mar 10;8:157. doi: 10.3389/fbioe.2020.00157 (PMC7076078; doi:10.3389/fbioe.2020.00157)
Supplement: Supplementary file 2 [file Data_Sheet_2.PDF]

Figure S1. Sequence alignment of JAZ1s and motif identification.

(a) Multiple sequence alignment of the JAZ1 proteins from *Gossypium arboreum*, *G. hirsutum*, *G. raimondii*, *Vitis vinifera*, *A. thaliana*, *Theobroma cacao*, *Brachypo diumdistachyon*, *Oryza sativa*, *Zea mays*, and *Sorghum bicolour* was obtained by DNAMAN software. TIFY and Jas motifs are each indicated by a red box.

(b) and (c) indicate TIFY and Jas motif identification.

Figure S2. The relative expression of *GhJAZ1* in allotetraploid cotton (*Gossypium hirsutum L*. acc. TM-1) based on a previous report (Zhang *et al.*, 2015).

Figure S3. Expression level of *GaJAZ1* in leaves at 0, 3, 6, 12, and 24 h after high temperature treatment (40 ℃) and low temperature treatment (4 ℃).

Figure S4. The activity of CAT and SOD, MDA contents, antioxidant capacity (T-AOC) in leaves of *TRV::00* and *TRV::GaJAZ1* plants after treatment as shown in (a), (b), (c) and (d). Bars represent SD of three independent biological replicates. The single asterisk indicates statistical significance at $P<0.05$. The double asterisk indicates statistical significance at $P<0.01$.

Figure S5. Subcellular localization of the GaJAZ1 protein.

(a) The vector of 35S:: *GaJAZ1::*GFP.

(b) The fluorescence distribution of 35S:: *GaJAZ1::*GFP and 35S::GFP.

Figure S6. GaMYC2.1 promote the GUS expression driven by GaMYB59 promoter.

(a) GaMYC2.1 promote the expression of GUS driven by GaMYB59 promoter. A 2 kb *GaMYB59* upstream DNA fragment driving GUS was used.

(b) The GUS activity measured by transient GUS activity assays is shown (Tukey's multiple range test).

Figure S7. Expression level of eight individual lines in *GaJAZ1*-OE plants.

(a) Semi-RT-PCR analysis of the eight individual lines in *GaJAZ1*-OE plants.

(b) Quantitative real-time PCR analysis of the relative expression levels of eight individual lines in *GaJAZ1*-OE plants. L1-L8 are eight individual lines. Bar represents SD of three independent biological replicates.

Figure S8. The phenotypes of *GaJAZ1*-OE and WT plants in the field.

Figure S9. Germination rates of WT and *GaJAZ1*-OE seeds under salt treatment.

(a) Germination phenotypes of WT and *GaJAZ1*-OE with the 6‰ or 10‰ NaCl treatments.

(b) Comparison of germination rates under different salt concentrations.

Bars represent SD of three independent biological replicates. The double asterisk indicates statistical significance at $P<0.01$.

Figure S10. Phenotypes of WT and *GaJAZ1*-OE plants at the three growth periods in the greenhouse. Bars =5cm.

Figure S11. Sequence alignment of JAZ1s from *G. arboreum* and *G. hirsutum* by DNAMAN software. TIFY and Jas motifs are indicated by a red box, respectively.

Figure S12. WT and *GhJAZ1*-OE phenotypes in artificial salt pools.

(a) The phenotypes of *GhJAZ1*-OE and WT plants in artificial salt pools (the salt concentration was about 3‰).

(b) Comparison of seedling emergence rates in WT and *GhJAZ1*-OE plants.

Figure S13. Root system in WT and *GaJAZ1*-OE plants after treatment with 3‰ NaCl.

(a), (b), and (c) Average diameter, total surface area, and total volume of root (RootVolume).

Bars represent SD of three independent biological replicates. The single asterisk indicates statistical significance at $P<0.05$. The double asterisk indicates statistical significance at $P<0.01$.

Figure S14. WT and *GaJAZ1*-OE phenotypes in saline-alkaline soil.

(a) and (b) WT and *GaJAZ1*-OE phenotypes during the flowering period.

(c)The maturation phenotypes of WT and *GaJAZ1*-OE plants. Bars =5cm.

Figure S15. Gene profiles in WT and *GaJAZ1*-OE plants after treatment for different time periods.

(a) Pearson correlation between the WT and *GaJAZ1*-OE plants.

(b) Venn diagram showing the overlap of differentially expressed genes between the four time point comparisons; cutoff=2.

(c) The KEGG classification of assembled unigenes from untreated WT and *GaJAZ1*-OE plants.

Figure S16. Compartment-specific gene sets identified in WT and *GaJAZ1*-OE plants.

(a) and (b) Up- and down-regulated genes involved in the JA signalling metabolic pathways.

(c) Up- and down-regulated genes involved in the JA signalling synthesis pathways.

Figure S17. Knockdown of *GhMYB59* increased the plant tolerance to salt stress.

(a) The phenotypes of *TRV::00* and *TRV:: GhMYB59* plants18 days after treatment with 300 mM NaCl.

(b) and (c) The silencing efficiency and survival rates of *GhMYB59* in *TRV::00* and *TRV:: GhMYB59* plants, n=31.

(d) Quantitative real-time PCR analysis of the expression patterns of key genes involved in salt stress responses in *TRV::00*, *TRV:: GhMYB59* after treatment with 300 mM NaCl.

Bars represent SD of three independent biological replicates and four technical repeat experiments. The single asterisk indicates statistical significance at $P<0.05$. The double asterisk indicates statistical significance at $P<0.01$.

Figure S18. Expression level of *DFL2*, *MYB13* and *CIPK9*.

(a) Quantitative real-time PCR analysis of the expression level of *GhDFL2*, *GhMYB13* and *GhCIPK9* in *TRV::00*, *TRV:: GhMYB59*.

(b) Quantitative real-time PCR analysis of the expression level of *GaDFL2, GaMYB13* and *GaCIPK9* in *TRV::00*and *TRV::GaJAZ1* plants.

(c) The scaled FPKM values of *GhDFL2, GhMYB13* and *GhCIPK9* in *GaJAZ1*-OE plants compared to the WT.

(d) Quantitative real-time PCR analysis of the expression level of *GhDFL2, GhMYB13* and *GhCIPK9*.

Bar represents standard deviation (SD) of three independent biological replicates and four technical repeat experiments. The single asterisk indicates statistical significance at $P<0.05$. The double asterisk indicates statistical significance at $P<0.01$.
