# Supplementary material for: Gossypium hirsutum Salt Tolerance Is Enhanced by Overexpression of G. arboreum JAZ1
Source: Front Bioeng Biotechnol. 2020 Mar 10;8:157. doi: 10.3389/fbioe.2020.00157 (PMC7076078; doi:10.3389/fbioe.2020.00157)
Supplement: Supplementary file 3 [file Data_Sheet_3.pdf]

**Table S1.** The chemical component of the Hoagland solution.

| | Chemical component | solution(mmol/L) |
|---|---|---|
| solution Ⅰ | H3BO3 | 0.02 |
| | ZnSO4 ·7H2O | 0.001 |
| | CuSO4 ·5H2O | 0.0002 |
| | MgSO4 ·H2O | 0.001 |
| | (NH4)6Mo7O24 ·4H2O | 0.000005 |
| solution Ⅱ | Ca(NO3)2 ·4H2O | 2.49841 |
| solutionⅢ | KH2PO4 | 0.49967 |
| | KNO3 | 2.5047 |
| solutionⅣ | EDTA ·FeNa | 0.1 |
| solution Ⅴ | MgSO4 ·7H2O | 1.00012 |

**Table S2.** List of the primers.

| Primer Name | Sequence | Experiment |
|---|---|---|
| Promoter-F | TTGAATAGATACGCTGACACGC | Cloning |
| GaJAZ1-F | ATGTTTGGTTCACCGGAATATACAT | Cloning |
| GaJAZ1-R | CTATCCCTTTCTCTTCTCG | Cloning |
| GaMYC2.1-AD-F | GCCATGGAGGCCAGTGAATTCATGAAGGACCATAGGTTAG | Yeast two hybrid assay |
| GaMYC2.1-AD-R | CGATTCATCTGCAGCTCGAGCTTATCTTGCATCTCCAAC | Yeast two hybrid assay |
| GaMYC2.2-AD-F | GCCATGGAGGCCAGTGAATTCATGACTGACTATCGATTTG | Yeast two hybrid assay |
| GaMYC2.2-AD-R | CGATTCATCTGCAGCTCGAGCCTATATCTCACTTCCGATTTTG | Yeast two hybrid assay |
| AtMYC2-AD-F | GCCATGGAGGCCAGTGAATTC ATGACTGATTACCGGCTAC | Yeast two hybrid assay |
| AtMYC2-AD-R | CGATTCATCTGCAGCTCGAG CTTAACCGATTTTTGAAATC | Yeast two hybrid assay |
| GaJAZ1-BD-F | ATGGAGGCCGAATTCATGTTTGGTTCACCGGAATATAC | Yeast two hybrid assay |
| GaJAZ1-BD-R | CAGGTCGACGGATCCCTACTGTAGTGATTCAACAG | Yeast two hybrid assay |
| GaJAZ1-△jas-BD-F | ATGGAGGCCGAATTCATGTTTGGTTCACCGGAATATAC | Yeast two hybrid assay |
| GaJAZ1-△jas-BD-R | TTATGCTAGTTATGCGGCCGCTAGATCGCCGGGAATGGGACG | Yeast two hybrid assay |
| AtJAZ1-BD-F | ATGGAGGCCGAATTCATGTCGAGTTCTATGGAATG | Yeast two hybrid assay |
| AtJAZ1-BD-R | CAGGTCGACGGATCCTCATATTTCAGCTGCTAAAC | Yeast two hybrid assay |
| AtJAZ1-△jas-BD-F | ATGGAGGCCGAATTCATGTCGAGTTCTATGGAATG | Yeast two hybrid assay |
| AtJAZ1-△jas-BD-R | TTATGCTAGTTATGCGGCCGCAAGTTCTGTCAATGGTGTTGGGGAG | Yeast two hybrid assay |
| nluc-GaJAZ1-F | CATTTCATTTGGAGAGAACACGGGGGACGGGGGTACCATGTTTGGTTCACCGGAATATAC | Firefly luciferase complementation |
| nluc-GaJAZ1-R | GAGCCACCGCCCCGGGACGCGTACGAGATCTGGGG GTCGACCTACTGTAGTGATTCAACAG | Firefly luciferase complementation |
| nluc-GaJAZ1-△jas-F | CATTTCATTTGGAGAGAACACGGGGGACGGGGGTACCATGTTTGGTTCACCGGAATATAC | Firefly luciferase complementation |
| nluc-GaJAZ1-△jas-R | GAGCCACCGCCCCGGGACGCGTACGAGATCTGGGGGTCGACTAGATCGCCGGGAATGGGACG | Firefly luciferase complementation |
| cluc-GaMYC2.1-F | CGGAGGTCAGATCTCGTACGCGTCCCGGGGCGGGGGTACCATGAAGGACCATAGGTTAG | Firefly luciferase complementation |
| cluc-GaMYC2.2-F | CGGAGGTCAGATCTCGTACGCGTCCCGGGGCGGG GGTACC ATGACTGACTATCGATTTG | Firefly luciferase complementation |
| cluc-GaMYC2.2-R | GTCGTCGTCGTCCTTGTAGTCCATTTGTTGGGGTCGACCCTATATCTCACTTCCGATTTTG | Firefly luciferase complementation |

| Primer Name | Sequence | Experiment |
|---|---|---|
| GaMYB59-F | TGCTTTGATCATATTCCTTGAAAAT | Reporter-effector |
| GaMYB59-R | GTTTCTTTCTCCTCTAAATCCG | Reporter-effector |
| GaJAZ1-F | GAGAACACGGGGGACTCTAGAATGTTTGGTTCACCGGAATATAC | Reporter-effector |
| GaJAZ1-R | GGACTGACCACCCGGGGATCCCTACTGTAGTGATTCAACAG | Reporter-effector |
| GaJAZ1-△jas-R | GGACTGACCACCCGGGGATCCTAGATCGCCGGGAATGGGACG | Reporter-effector |
| GaMYC2.1-F | GAGAACACGGGGGACTCTAGAATGAAGGACCATAGGTTAG | Reporter-effector |
| GaMYC2.1-R | GGACTGACCACCCGGGGATCCCTTATCTTGCATCTCCAAC | Reporter-effector |
| GaJAZ1-F | GGTAGTCCGAACGAATCCGGCC | Subcellular localization |
| GaJAZ1-R | TCGCCGGGAATGGAACGAACG | Subcellular localization |
| pYL156-GaJAZ1-F | GAATTCATGTTTGGTTCACCGG | Virus-induced gene silencing |
| pYL156-GaJAZ1-R | GGATCCCGGCGCTGCGGCGT | Virus-induced gene silencing |
| pYL156-GhMYB59-F | GAATTCATGAAACTTGTGCAA | Virus-induced gene silencing |
| pYL156-GhMYB59-R | GAATTCATGAAACTTGTGCAAGAAG | Virus-induced gene silencing |
| GaJAZ1-F | GGTAGTCCGAACGAATCCGGCC | qRT-PCR |
| GaJAZ1-R | TCGCCGGGAATGGAACGAACG | qRT-PCR |
| GaLOX3-F | AGAAAAGGAATGCCGATCCT | qRT-PCR |
| GaLOX3-R | AGGACCTGAGCTAGGTGCAA | qRT-PCR |
| GaAOS-F | CTTGACTGCCCATGACTGAA | qRT-PCR |
| GaAOS-R | GCACCACAAACCAAAGGACT | qRT-PCR |
| GaAOC4-F | AGAAGAGCTGCTTTGCAAGC | qRT-PCR |
| GaAOC4-R | CATGTGGCTCACAAGCCTTA | qRT-PCR |
| GaACS6-F | TGAAGAGCAATGCTGGATTG | qRT-PCR |
| GaACS6-R | ACTCGCCACAAGTCCATTTC | qRT-PCR |
| GaERF2-F | GGAGCCAGATCCAGTCAGAG | qRT-PCR |
| GaERF2-R | CTCCTTTTTGGTGACGCATT | qRT-PCR |
| GaERF4-F | CGTCCTCGGTTGTTGATTTT | qRT-PCR |
| GaERF4-R | CAAACGGGGAAAAGAATTCA | qRT-PCR |

| Primer Name | Sequence | Experiment |
|---|---|---|
| GaABR1-F | CCTATGGTTTTTCCGCTTCA | qRT-PCR |
| GaABR1-R | CCAAGAATGCACCGAAAAAT | qRT-PCR |
| GaABA2-F | ATTTTGACCCCATTGTTCCA | qRT-PCR |
| GaABA2-R | TTTCAACACTGCTGAAACCG | qRT-PCR |
| GaCBF4-F | GCTGACTCAGCTTGGAAGCT | qRT-PCR |
| GaCBF4-R | CAGCCGTTCTGAAAGTCTCC | qRT-PCR |
| GaRD26-F | AAAACTGTTTGGTCGGCAAG | qRT-PCR |
| GaRD26-R | TCAATTGAGCCAATGGATCA | qRT-PCR |
| GaCIPK9-F | CTCAGGAGGCCGATCCTACCAA | qRT-PCR |
| GaCIPK9-R | CGGTGGCGCAAGAGTACCATAC | qRT-PCR |
| GaMYB13-F | GCTGCACAGTTACCTGGACGAA | qRT-PCR |
| GaMYB13-R | AAGTTGGTTGTGGGGATGCTGA | qRT-PCR |
| GaDFL2-F | AAGCAGAGCAAGACCAAAGGGG | qRT-PCR |
| GaDFL2-R | AGATTGCATTGCTGTCATCGCT | qRT-PCR |
| GhLOX3-F | ACGTCGACCTTATCCGAGACGA | qRT-PCR |
| GhLOX3-R | CTTCCGGCATCCACTGATCGTT | qRT-PCR |
| GhAOS-F | CTGGTGACTGCGGACATACGTT | qRT-PCR |
| GhAOS-R | CAACACGAGGGCAAGAGGCAAT | qRT-PCR |
| GhACS6-F | GACATTGAAGAGCATCGTGCAGT | qRT-PCR |
| GhACS6-R | AGGGTCTTTGAAGACTGTGGCG | qRT-PCR |
| GhERF2-F | GAAACCAAACTGCCGTTGCGAG | qRT-PCR |
| GhERF2-R | CTGTTCGGCTGTTTCGAACGTG | qRT-PCR |
| GhERF4-F | CATCGGAGAGCGAAACGGAACA | qRT-PCR |
| GhERF4-R | CAGCTTCTTCAGCGGTGTCGTA | qRT-PCR |
| GhABR1-F | AAGCAGCCCTTGGTTTCAGAGG | qRT-PCR |
| GhABR1-R | GAAAACGGGCAACAGGGTTGTG | qRT-PCR |
| GhABA2-F | CATTGCTTTACGTCGGCGTTCC | qRT-PCR |

| Primer Name | Sequence | Experiment |
|---|---|---|
| GhABA2-R | CATAGGAGTCGCAATGCCGTGA | qRT-PCR |
| GhCBF4-F | TCAGAACGGCTGAGCATTCGAG | qRT-PCR |
| GhCBF4-R | CCCAAACAACGCTTCTTCGTCC | qRT-PCR |
| GhRD26-F | TCAAGCAGAGAGCAAAGCACGA | qRT-PCR |
| GhRD26-R | GAACGAGTTAATGCGCGGCAAA | qRT-PCR |
| GhGH3.6-F | ACCATTTGCTGCCATTTGGTGC | qRT-PCR |
| GhGH3.6-R | GCCCTTGGCGGTATACACTGTT | qRT-PCR |
| GhCIPK9-F | AGAATGTTGAGACCGGCGAGTG | qRT-PCR |
| GhCIPK9-R | ACTGTTCGACCATTCGGTGACG | qRT-PCR |
| GhMYB13-F | AGCATCCCCACAACCAACTTCA | qRT-PCR |
| GhMYB13-R | CCACTATTTGCCTCGTACCCGT | qRT-PCR |
| GhDFL2-F | AATGTGGACGTATCGGCTGACC | qRT-PCR |
| GhDFL2-R | TCCCTTCCAAACAACTCGGAGC | qRT-PCR |
| GhMYB59-F | GAATTCATGAAAATGATGCAAGAGG | qRT-PCR |
| GhMYB59-R | GGATCCCCTGTTTCCCCACTTAG | qRT-PCR |
| Gossypium hirsutum histone 3Forward Primer | CCTTGTGGGTCTTTTTGAA | qRT-PCR |
| Gossypium hirsutum histone 3 Reverse Primer | AACTGGATGTCCTTGGGC | qRT-PCR |

**Table S3.** Fiber quality in transgenic cotton and the control (CCRI24) .

| Line | Fiber length (mm) | Length uniformity (%) | Micronaire value | Fiber strength (cN/tex) |
|---|---|---|---|---|
| L2 | 28.2 | 85.3 | 4.3 | 26.6 |
| L4 | 30.0 | 85.7 | 4.3 | 27.5 |
| L5 | 31.3 | 86.1 | 4.0 | 26.7 |
| control | 29.7 | 87.1 | 4.4 | 27.9 |

The plot mean values were based on the average over 15 individual plants.

**Table S4.** Summary of sequencing results.

| Samples | Clean reads | clean bases | Total mapped | Q30 (%) | GC content (%) |
|---|---|---|---|---|---|
| WT-0h | 5.47E+07 | 8.20G | 4.76E+07 | 93.28 | 43.92 |
| *GaJAZ1*-OE-0h | 5.36E+07 | 8.05G | 4.72E+07 | 93.29 | 43.76 |
| WT-6h | 5.53E+07 | 8.29G | 4.29E+07 | 93.65 | 43.86 |
| *GaJAZ1*-OE-6h | 5.02E+07 | 7.52G | 4.42E+07 | 93.37 | 43.68 |
| WT-12h | 5.17E+07 | 7.75G | 4.42E+07 | 92.96 | 43.62 |
| *GaJAZ1*-OE-12h | 5.26E+07 | 7.89G | 4.60E+07 | 93.51 | 43.66 |
| WT-24h | 5.27E+07 | 7.91G | 4.61E+07 | 93.08 | 43.78 |
| *GaJAZ1*-OE-24h | 4.92E+07 | 7.38G | 4.29E+07 | 94.60 | 43.66 |

**Table S5.** Distribution of FPKM interval in *GaJAZ1*-OE and WT plants at different times.

| FPKM Interval | 0~1 | Percent | 1~3 | Percent | 3~15 | Percent | 15~60 | Percent | >60 | Percent |
|---|---|---|---|---|---|---|---|---|---|---|
| WT-0h | 26605 | 34.34% | 11209 | 14.47% | 25295 | 32.65% | 11592 | 14.96% | 2783 | 3.59% |
| *GAJAZ1*-OE-0h | 27175 | 35.07% | 11395 | 14.71% | 25114 | 32.41% | 10985 | 14.18% | 2814 | 3.63% |
| WT-6h | 26687 | 34.44% | 11179 | 14.43% | 24681 | 31.85% | 11817 | 15.25% | 3120 | 4.03% |
| *GAJAZ1*-OE-6h | 27384 | 35.34% | 11241 | 14.51% | 24405 | 31.50% | 11581 | 14.95% | 2873 | 3.71% |
| WT-12h | 25980 | 33.53% | 10778 | 13.91% | 25106 | 32.40% | 12623 | 16.29% | 2997 | 3.87% |
| *GAJAZ1*-OE-12h | 26332 | 33.98% | 10750 | 13.88% | 24900 | 32.13% | 12493 | 16.13% | 3008 | 3.88% |
| WT-24h | 27480 | 35.47% | 11075 | 14.29% | 24456 | 31.56% | 11510 | 14.86% | 2963 | 3.82% |
| *GAJAZ1*-OE-24h | 26822 | 34.62% | 11152 | 14.39% | 24721 | 31.91% | 11785 | 15.21% | 3003 | 3.87% |

**Table S6.** Differentially expressed genes in different groups.

| sample | sample | log2FoldChange≧2 | up | down |
|---|---|---|---|---|
| WT_6h | WT_0h | 4761 | 2532 | 2229 |
| WT_12h | WT_6h | 1413 | 425 | 988 |
| WT_24h | WT_12h | 2580 | 1456 | 1124 |
| *GaJAZ1*-OE_6h | *GaJAZ1*-OE_0h | 5886 | 2582 | 3304 |
| *GaJAZ1*-OE_12h | *GaJAZ1*-OE_6h | 1459 | 599 | 860 |
| *GaJAZ1*-OE_24h | *GaJAZ1*-OE_12h | 1934 | 1194 | 740 |
| *GaJAZ1*-OE_0h | WT_0h | 1146 | 377 | 769 |
| *GaJAZ1*-OE_6h | WT_6h | 520 | 146 | 374 |
| *GaJAZ1*-OE_12h | WT_12h | 576 | 240 | 336 |

**Table S7.** Genes exclusively by salt stress between *GaJAZ1*-OE and WT plants**.**

| Gene ID | log2 (GaJAZ1/WT) | Arabidopsis ID | Annotation (acc. To TAIR) |
|---|---|---|---|
| Gh_A01G0055 | -2.2709 | AT3G19000 | 2-oxoglutarate (2OG) and Fe (II)-dependent oxygenase superfamily protein |
| Gh_A01G0347 | -2.0502 | AT2G21110 | Disease resistance-responsive (dirigent-like protein) family protein |
| Gh_A01G0382 | -5.105 | AT5G46330 | Leucine-rich receptor-like protein kinase family protein |
| Gh_A01G0384 | -2.0615 | AT4G20140 | Leucine-rich repeat transmembrane protein kinase |
| Gh_A01G0467 | -2.8541 | AT4G21700 | Protein of unknown function |
| Gh_A01G0526 | -2.4629 | AT4G21440 | MYB-like 102 |
| Gh_A01G0547 | -2.5331 | AT5G54510 | Auxin-responsive GH3 family protein |
| Gh_A01G0613 | -2.1755 | AT5G36110 | cytochrome P450, family 716, subfamily A, polypeptide 1 |
| Gh_A01G0767 | -2.3773 | AT5G07280 | Leucine-rich repeat transmembrane protein kinase |
| Gh_A01G0966 | -2.5167 | AT2G26530 | Protein of unknown function |
| Gh_A01G1477 | -3.0019 | AT1G68150 | WRKY DNA-binding protein 9 |
| Gh_A01G1655 | -2.2389 | AT5G06839 | bZIP transcription factor family protein |
| Gh_A01G1658 | -2.7324 | AT5G06900 | cytochrome P450, family 93, subfamily D, polypeptide 1 |
| Gh_A01G1659 | -3.0697 | AT5G06900 | cytochrome P450, family 93, subfamily D, polypeptide 1 |
| Gh_A01G1660 | -2.4235 | AT2G42250 | cytochrome P450, family 712, subfamily A, polypeptide 1 |
| Gh_A01G1887 | -2.1581 | AT3G06880 | Transducin/WD40 repeat-like superfamily protein |
| Gh_A01G2141 | 3.2525 | AT1G52340 | NAD(P)-binding Rossmann-fold superfamily protein |
| Gh_A02G0011 | 2.3637 | AT5G01990 | Auxin efflux carrier family protein |
| Gh_A02G0050 | -2.7755 | AT4G36220 | ferulic acid 5-hydroxylase 1 |
| Gh_A02G0330 | -2.1027 | AT1G60680 | NAD(P)-linked oxidoreductase superfamily protein |
| Gh_A02G0741 | -3.368 | AT1G77330 | 2-oxoglutarate (2OG) and Fe(II)-dependent oxygenase superfamily protein |
| Gh_A02G0866 | 2.1881 | AT4G25810 | xyloglucan endotransglycosylase 6 |
| Gh_A02G1014 | -3.0695 | AT5G41610 | cation/H+ exchanger 18 |

| Gene ID | log2 (GaJAZ1/WT) | Arabidopsis ID | Annotation (acc. To TAIR) |
|---|---|---|---|
| Gh_A02G1017 | -2.7688 | AT5G41610 | cation/H+ exchanger 18 |
| Gh_A02G1175 | 2.7957 | AT3G15990 | sulfate transporter 3;4 |
| Gh_A02G1197 | -2.6326 | AT1G52800 | 2-oxoglutarate (2OG) and Fe(II)-dependent oxygenase superfamily protein |
| Gh_A02G1241 | 4.3938 | AT1G72210 | basic helix-loop-helix (bHLH) DNA-binding superfamily protein |
| Gh_A02G1604 | -2.0935 | AT1G27660 | basic helix-loop-helix (bHLH) DNA-binding superfamily protein |
| Gh_A02G1734 | -2.5021 | AT1G06620 | 2-oxoglutarate (2OG) and Fe(II)-dependent oxygenase superfamily protein |
| Gh_A03G0401 | 2.077 | AT1G12740 | cytochrome P450, family 87, subfamily A, polypeptide 2 |
| Gh_A03G0507 | 2.0413 | AT1G12240 | Glycosyl hydrolases family 32 protein |
| Gh_A03G0991 | -2.3202 | AT2G01900 | DNAse I-like superfamily protein |
| Gh_A03G1411 | 3.0364 | AT4G10265 | Wound-responsive family protein |
| Gh_A03G1412 | 3.3112 | AT4G10265 | Wound-responsive family protein |
| Gh_A03G1493 | -2.353 | AT4G38140 | RING/U-box superfamily protein |
| Gh_A03G1537 | 2.371 | AT1G17840 | white-brown complex homolog protein 11 |
| Gh_A03G1776 | Inf | AT4G38840 | SAUR-like auxin-responsive protein family |
| Gh_A03G1935 | -2.3817 | AT2G35060 | K+ uptake permease 11 |
| Gh_A03G2015 | -2.7674 | AT4G23030 | MATE efflux family protein |
| Gh_A03G2044 | -2.0772 | AT4G11650 | osmotin 34 |
| Gh_A03G2148 | -4.9216 | AT4G35150 | O-methyltransferase family protein |
| Gh_A03G2159 | 2.8321 | AT5G02600 | Heavy metal transport/detoxification superfamily protein |
| Gh_A04G0071 | 2.6514 | AT2G21110 | Disease resistance-responsive (dirigent-like protein) family protein |
| Gh_A04G0284 | -2.3029 | AT4G27220 | NB-ARC domain-containing disease resistance protein |
| Gh_A04G0392 | -2.4893 | AT1G55020 | lipoxygenase 1 |
| Gh_A04G0485 | -3.3172 | AT5G06200 | Uncharacterised protein family |
| Gh_A04G0558 | -2.2082 | AT3G52970 | cytochrome P450, family 76, subfamily G, polypeptide 1 |
| Gh_A04G0578 | -2.8724 | AT4G40070 | RING/U-box superfamily protein |
| Gh_A04G0634 | -2.3846 | AT1G61110 | NAC domain containing protein 25 |

| Gene ID | log2 (GaJAZ1/WT) | Arabidopsis ID | Annotation (acc. To TAIR) |
|---|---|---|---|
| Gh_A04G0810 | -2.3143 | AT5G66770 | GRAS family transcription factor |
| Gh_A04G0848 | 2.829 | AT5G56660 | IAA-leucine resistant (ILR)-like 2 |
| Gh_A04G0921 | 2.2106 | AT2G31180 | myb domain protein 14 |
| Gh_A04G0996 | -2.3895 | AT5G42500 | Disease resistance-responsive (dirigent-like protein) family protein |
| Gh_A04G1209 | -2.7992 | AT1G74110 | cytochrome P450, family 78, subfamily A, polypeptide 10 |
| Gh_A04G1216 | -2.64 | AT4G10310 | high-affinity K+ transporter 1 |
| Gh_A04G1361 | -3.2167 | AT2G24130 | Leucine-rich receptor-like protein kinase family protein |
| Gh_A05G0014 | 2.0168 | AT5G57090 | Auxin efflux carrier family protein |
| Gh_A05G0164 | -2.2544 | AT3G24520 | heat shock transcription factor C1 |
| Gh_A05G0423 | -3.9333 | AT5G48930 | hydroxycinnamoyl-CoA shikimate/quinate hydroxycinnamoyl transferase |
| Gh_A05G0834 | -2.0534 | AT4G01470 | tonoplast intrinsic protein 1;3 |
| Gh_A05G0889 | -2.1871 | AT5G60660 | plasma membrane intrinsic protein 2;4 |
| Gh_A05G1449 | -2.3992 | AT3G21690 | MATE efflux family protein |
| Gh_A05G1467 | 2.4921 | AT4G22030 | F-box family protein with a domain of unknown function |
| Gh_A05G1468 | 3.8079 | AT4G22030 | F-box family protein with a domain of unknown function |
| Gh_A05G1469 | 2.6281 | AT4G22030 | F-box family protein with a domain of unknown function |
| Gh_A05G1576 | 2.2426 | AT1G20190 | expansin 11 |
| Gh_A05G1741 | -2.14 | AT2G26570 | Plant protein of unknown function |
| Gh_A05G1779 | 2.0831 | AT4G34150 | Calcium-dependent lipid-binding (CaLB domain) family protein |
| Gh_A05G2391 | -3.0587 | AT1G11300 | protein serine/threonine kinases |
| Gh_A05G2501 | 2.0271 | AT5G04460 | RING/U-box superfamily protein |
| Gh_A05G2516 | -2.3526 | AT4G36220 | ferulic acid 5-hydroxylase 1 |
| Gh_A05G2682 | -2.0144 | AT2G01430 | homeobox-leucine zipper protein 17 |
| Gh_A05G2878 | -2.4283 | AT5G05600 | 2-oxoglutarate (2OG) and Fe(II)-dependent oxygenase superfamily protein |
| Gh_A05G2921 | 2.1169 | AT1G28050 | B-box type zinc finger protein with CCT domain |
| Gh_A05G3413 | -2.3506 | AT4G23920 | UDP-D-glucose/UDP-D-galactose 4-epimerase 2 |

| Gene ID | log2 (GaJAZ1/WT) | Arabidopsis ID | Annotation (acc. To TAIR) |
|---|---|---|---|
| Gh_A05G3493 | 2.6775 | AT2G40610 | expansin A8 |
| Gh_A05G3748 | -2.8209 | AT4G08250 | GRAS family transcription factor |
| Gh_A05G3945 | -2.5551 | AT3G14690 | cytochrome P450, family 72, subfamily A, polypeptide 15 |
| Gh_A05G3970 | -2.5183 | AT5G43470 | Disease resistance protein (CC-NBS-LRR class) family |
| Gh_A06G0018 | 3.1123 | AT1G20190 | expansin 11 |
| Gh_A06G0187 | -2.0365 | AT3G53010 | Domain of unknown function |
| Gh_A06G0228 | -2.0588 | AT3G24240 | Leucine-rich repeat receptor-like protein kinase family protein |
| Gh_A06G0358 | -2.8822 | AT4G08250 | GRAS family transcription factor |
| Gh_A06G0497 | -2.0682 | AT2G26650 | K+ transporter 1 |
| Gh_A06G0563 | -2.0635 | AT4G28890 | RING/U-box superfamily protein |
| Gh_A06G0829 | -3.8829 | AT4G32860 | unknown protein |
| Gh_A06G0972 | -4.5314 | AT5G24800 | basic leucine zipper 9 |
| Gh_A06G0999 | -2.1845 | AT5G09970 | cytochrome P450, family 78, subfamily A, polypeptide 7 |
| Gh_A06G1070 | 2.205 | AT4G23250 | kinases;protein kinases |
| Gh_A06G1542 | 6.0878 | AT2G02990 | ribonuclease 1 |
| Gh_A06G1600 | -3.0978 | AT1G11300 | protein serine/threonine kinases |
| Gh_A06G1649 | -3.6225 | AT5G46330 | Leucine-rich receptor-like protein kinase family protein |
| Gh_A06G1699 | -2.6378 | AT5G48930 | hydroxycinnamoyl-CoA shikimate/quinate hydroxycinnamoyl transferase |
| Gh_A06G1701 | -2.3036 | AT5G48930 | hydroxycinnamoyl-CoA shikimate/quinate hydroxycinnamoyl transferase |
| Gh_A06G1730 | -2.8769 | AT5G47740 | Adenine nucleotide alpha hydrolases-like superfamily protein |
| Gh_A06G1755 | -2.2349 | AT1G12030 | Protein of unknown function |
| Gh_A06G1765 | 3.7816 | AT4G22030 | F-box family protein with a domain of unknown function |
| Gh_A06G1802 | 3.0018 | AT1G17420 | lipoxygenase 3 |
| Gh_A06G1803 | 2.149 | AT1G17420 | lipoxygenase 3 |
| Gh_A07G0229 | -2.7448 | AT5G44700 | Leucine-rich repeat transmembrane protein kinase |
| Gh_A07G0328 | -2.2198 | AT1G15550 | gibberellin 3-oxidase 1 |

| Gene ID | log2 (GaJAZ1/WT) | Arabidopsis ID | Annotation (acc. To TAIR) |
|---|---|---|---|
| Gh_A07G0450 | -3.2086 | AT1G12740 | cytochrome P450, family 87, subfamily A, polypeptide 2 |
| Gh_A07G0553 | -2.2014 | AT1G63800 | ubiquitin-conjugating enzyme 5 |
| Gh_A07G0723 | -3.5072 | AT3G30210 | myb domain protein 121 |
| Gh_A07G1349 | -2.2321 | AT2G45430 | AT-hook motif nuclear-localized protein 22 |
| Gh_A07G1547 | -2.3409 | AT1G66140 | zinc finger protein 4 |
| Gh_A07G1611 | 2.7109 | AT5G15780 | Pollen Ole e 1 allergen and extensin family protein |
| Gh_A07G2016 | -2.1583 | AT1G23740 | Oxidoreductase, zinc-binding dehydrogenase family protein |
| Gh_A07G2033 | 2.8896 | AT5G14650 | Pectin lyase-like superfamily protein |
| Gh_A08G0064 | -2.1085 | AT2G27370 | Uncharacterised protein family |
| Gh_A08G0179 | Inf | AT4G27350 | Protein of unknown function |
| Gh_A08G0182 | Inf | AT1G53050 | Protein kinase superfamily protein |
| Gh_A08G0346 | 3.1213 | AT4G11290 | Peroxidase superfamily protein |
| Gh_A08G0347 | 2.688 | AT3G01190 | Peroxidase superfamily protein |
| Gh_A08G0417 | -2.4695 | AT3G23240 | ethylene response factor 1 |
| Gh_A08G0580 | -2.1428 | AT2G41100 | Calcium-binding EF hand family protein |
| Gh_A08G0962 | 2.0744 | AT1G32640 | Basic helix-loop-helix (bHLH) DNA-binding family protein |
| Gh_A08G0990 | -2.6352 | AT1G01490 | Heavy metal transport/detoxification superfamily protein |
| Gh_A08G1196 | -2.1432 | AT2G45430 | AT-hook motif nuclear-localized protein 22 |
| Gh_A08G1210 | -2.6065 | AT2G45360 | Protein of unknown function |
| Gh_A08G1400 | -2.1714 | AT5G16770 | myb domain protein 9 |
| Gh_A08G1572 | -2.1181 | AT4G12110 | sterol-4alpha-methyl oxidase 1-1 |
| Gh_A08G1613 | 2.2937 | AT4G23030 | MATE efflux family protein |
| Gh_A08G1684 | -2.3102 | AT3G28730 | high mobility group |
| Gh_A08G1778 | -2.1873 | AT1G08090 | nitrate transporter 2:1 |
| Gh_A08G1885 | -2.062 | AT1G54740 | Protein of unknown function |
| Gh_A08G1950 | 2.7909 | AT5G17820 | Peroxidase superfamily protein |

| Gene ID | log2 (GaJAZ1/WT) | Arabidopsis ID | Annotation (acc. To TAIR) |
|---|---|---|---|
| Gh_A08G2059 | -4.0565 | AT4G27190 | NB-ARC domain-containing disease resistance protein |
| Gh_A08G2199 | 4.2063 | AT1G19180 | jasmonate-zim-domain protein 1 |
| Gh_A08G2362 | Inf | AT2G42430 | lateral organ boundaries-domain 16 |
| Gh_A08G2388 | -2.6857 | AT1G63440 | heavy metal atpase 5 |
| Gh_A08G2500 | -2.3885 | AT2G18960 | H(+)-ATPase 1 |
| Gh_A08G2559 | -2.1412 | AT2G37170 | plasma membrane intrinsic protein 2 |
| Gh_A09G0142 | -2.582 | AT1G26800 | RING/U-box superfamily protein |
| Gh_A09G0159 | -2.238 | AT1G26870 | NAC (No Apical Meristem) domain transcriptional regulator superfamily protein |
| Gh_A09G0474 | -2.2588 | AT3G18400 | NAC domain containing protein 58 |
| Gh_A09G0566 | -2.2447 | AT3G54700 | phosphate transporter 1;7 |
| Gh_A09G0903 | -2.1256 | AT1G08320 | bZIP transcription factor family protein |
| Gh_A09G1165 | 2.6971 | AT3G57670 | C2H2-type zinc finger family protein |
| Gh_A09G1179 | -2.248 | AT5G06905 | cytochrome P450, family 712, subfamily A, polypeptide 2 |
| Gh_A09G1181 | -2.6631 | AT5G06900 | cytochrome P450, family 93, subfamily D, polypeptide 1 |
| Gh_A09G1261 | 2.1151 | AT3G52450 | plant U-box 22 |
| Gh_A09G1353 | -2.8609 | AT4G18250 | receptor serine/threonine kinase, putative |
| Gh_A09G1359 | -3.8969 | AT2G39210 | Major facilitator superfamily protein |
| Gh_A09G1486 | 3.7677 | AT2G37430 | C2H2 and C2HC zinc fingers superfamily protein |
| Gh_A09G1649 | -2.7472 | AT1G07430 | highly ABA-induced PP2C gene 2 |
| Gh_A09G1775 | -2.747 | AT5G06900 | cytochrome P450, family 93, subfamily D, polypeptide 1 |
| Gh_A09G1848 | -2.0217 | AT5G07990 | Cytochrome P450 superfamily protein |
| Gh_A09G1849 | -2.7106 | AT5G07990 | Cytochrome P450 superfamily protein |
| Gh_A09G2283 | -2.4721 | AT1G06000 | UDP-Glycosyltransferase superfamily protein |
| Gh_A09G2284 | -2.1314 | AT1G06000 | UDP-Glycosyltransferase superfamily protein |
| Gh_A09G2474 | -2.1262 | AT2G28160 | FER-like regulator of iron uptake |
| Gh_A10G0054 | -2.1453 | AT1G30650 | WRKY DNA-binding protein 14 |

| Gene ID | log2 (GaJAZ1/WT) | Arabidopsis ID | Annotation (acc. To TAIR) |
|---|---|---|---|
| Gh_A10G0156 | -2.1596 | AT1G11300 | protein serine/threonine kinases |
| Gh_A10G0292 | -2.0068 | AT1G68810 | basic helix-loop-helix (bHLH) DNA-binding superfamily protein |
| Gh_A10G0340 | 3.1899 | AT2G40330 | PYR1-like 6 |
| Gh_A10G0436 | -2.2037 | AT1G31320 | LOB domain-containing protein 4 |
| Gh_A10G0493 | -2.3516 | AT3G13840 | GRAS family transcription factor |
| Gh_A10G0697 | -2.9339 | AT1G52790 | 2-oxoglutarate (2OG) and Fe(II)-dependent oxygenase superfamily protein |
| Gh_A10G1055 | -3.0586 | AT1G11300 | protein serine/threonine kinases |
| Gh_A10G1124 | -2.2207 | AT2G32830 | phosphate transporter 1;5 |
| Gh_A10G1129 | -3.8199 | AT3G11080 | receptor like protein 35 |
| Gh_A10G1332 | 2.2328 | AT5G59320 | lipid transfer protein 3 |
| Gh_A10G1537 | 3.9923 | AT3G17070 | Peroxidase family protein |
| Gh_A10G2073 | -2.4607 | AT1G69550 | disease resistance protein (TIR-NBS-LRR class) |
| Gh_A10G2081 | -3.0276 | AT3G25510 | disease resistance protein (TIR-NBS-LRR class), putative |
| Gh_A10G2089 | -2.2808 | AT4G00430 | plasma membrane intrinsic protein 1;4 |
| Gh_A10G2101 | -2.2348 | AT4G20140 | Leucine-rich repeat transmembrane protein kinase |
| Gh_A10G2148 | -2.7922 | AT4G20140 | Leucine-rich repeat transmembrane protein kinase |
| Gh_A10G2231 | -2.2955 | AT2G47000 | ATP binding cassette subfamily B4 |
| Gh_A11G0234 | 4.0773 | AT1G62510 | Bifunctional inhibitor/lipid-transfer protein/seed storage 2S albumin superfamily protein |
| Gh_A11G0378 | -2.617 | AT1G48480 | receptor-like kinase 1 |
| Gh_A11G0410 | -2.8317 | AT4G36740 | homeobox protein 40 |
| Gh_A11G0743 | -2.0484 | AT2G45430 | AT-hook motif nuclear-localized protein 22 |
| Gh_A11G0750 | -2.2767 | AT2G45510 | cytochrome P450, family 704, subfamily A, polypeptide 2 |
| Gh_A11G0773 | -2.4617 | AT3G61060 | phloem protein 2-A13 |
| Gh_A11G0906 | -2.0019 | AT2G46680 | homeobox 7 |
| Gh_A11G0942 | -2.7402 | AT3G53480 | pleiotropic drug resistance 9 |
| Gh_A11G1177 | -2.0585 | AT5G43580 | Serine protease inhibitor, potato inhibitor I-type family protein |

| Gene ID | log2 (GaJAZ1/WT) | Arabidopsis ID | Annotation (acc. To TAIR) |
|---|---|---|---|
| Gh_A11G1197 | -2.1716 | AT4G10500 | 2-oxoglutarate (2OG) and Fe(II)-dependent oxygenase superfamily protein |
| Gh_A11G1397 | -2.7966 | AT3G07870 | F-box and associated interaction domains-containing protein |
| Gh_A11G1398 | -2.8148 | AT3G07870 | F-box and associated interaction domains-containing protein |
| Gh_A11G1521 | -2.2062 | AT5G38280 | PR5-like receptor kinase |
| Gh_A11G1897 | -2.7274 | AT4G37850 | basic helix-loop-helix (bHLH) DNA-binding superfamily protein |
| Gh_A11G2144 | -2.4735 | AT1G52820 | 2-oxoglutarate (2OG) and Fe(II)-dependent oxygenase superfamily protein |
| Gh_A11G2463 | -3.298 | AT5G02070 | Protein kinase family protein |
| Gh_A11G2482 | -2.3294 | AT3G05725 | Protein of unknown function |
| Gh_A11G2552 | -2.6106 | AT1G11300 | protein serine/threonine kinases |
| Gh_A11G2576 | -2.8006 | AT1G28130 | Auxin-responsive GH3 family protein |
| Gh_A11G2620 | 5.1933 | AT4G38700 | Disease resistance-responsive (dirigent-like protein) family protein |
| Gh_A11G2752 | -2.6499 | AT3G56970 | basic helix-loop-helix (bHLH) DNA-binding superfamily protein |
| Gh_A11G2836 | -2.3271 | AT3G14470 | NB-ARC domain-containing disease resistance protein |
| Gh_A11G3000 | 3.9139 | AT1G74500 | activation-tagged BRI1(brassinosteroid-insensitive 1)-suppressor 1 |
| Gh_A11G3009 | -2.2417 | AT2G47140 | NAD(P)-binding Rossmann-fold superfamily protein |
| Gh_A12G0320 | 2.1163 | AT1G20440 | cold-regulated 47 |
| Gh_A12G0375 | -4.4277 | AT4G34760 | SAUR-like auxin-responsive protein family |
| Gh_A12G0581 | -2.3089 | AT1G19630 | cytochrome P450, family 722, subfamily A, polypeptide 1 |
| Gh_A12G0584 | 2.675 | AT3G11480 | S-adenosyl-L-methionine-dependent methyltransferases superfamily protein |
| Gh_A12G0736 | 3.8148 | AT2G22760 | basic helix-loop-helix (bHLH) DNA-binding superfamily protein |
| Gh_A12G0841 | 2.354 | AT5G10130 | Pollen Ole e 1 allergen and extensin family protein |
| Gh_A12G0842 | -2.2831 | AT5G65090 | DNAse I-like superfamily protein |
| Gh_A12G0875 | 2.3532 | AT2G44840 | ethylene-responsive element binding factor 13 |
| Gh_A12G0876 | Inf | AT2G44840 | ethylene-responsive element binding factor 13 |
| Gh_A12G0949 | -2.0901 | AT4G10160 | RING/U-box superfamily protein |
| Gh_A12G1006 | -2.1096 | AT2G46495 | RING/U-box superfamily protein |

| Gene ID | log2 (GaJAZ1/WT) | Arabidopsis ID | Annotation (acc. To TAIR) |
|---|---|---|---|
| Gh_A12G1029 | -2.5287 | AT2G46660 | cytochrome P450, family 78, subfamily A, polypeptide 6 |
| Gh_A12G1033 | -2.2973 | AT2G46680 | homeobox 7 |
| Gh_A12G1034 | -2.1847 | AT2G46690 | SAUR-like auxin-responsive protein family |
| Gh_A12G1508 | Inf | AT5G15290 | Uncharacterised protein family |
| Gh_A12G1680 | -3.9522 | AT4G20240 | cytochrome P450, family 71, subfamily A, polypeptide 27 |
| Gh_A12G1681 | -2.4263 | AT3G26310 | cytochrome P450, family 71, subfamily B, polypeptide 35 |
| Gh_A12G1830 | 2.2242 | AT5G45340 | cytochrome P450, family 707, subfamily A, polypeptide 3 |
| Gh_A12G2247 | -2.1222 | AT3G61850 | Dof-type zinc finger DNA-binding family protein |
| Gh_A12G2286 | -2.5953 | AT1G64065 | Late embryogenesis abundant (LEA) hydroxyproline-rich glycoprotein family |
| Gh_A12G2377 | 3.1624 | AT5G51790 | basic helix-loop-helix (bHLH) DNA-binding superfamily protein |
| Gh_A12G2397 | -2.3417 | AT4G12300 | cytochrome P450, family 706, subfamily A, polypeptide 4 |
| Gh_A12G2445 | 3.3093 | AT2G40540 | potassium transporter 2 |
| Gh_A12G2449 | 3.5709 | AT5G37490 | ARM repeat superfamily protein |
| Gh_A12G2619 | -2.5065 | AT5G50760 | SAUR-like auxin-responsive protein family |
| Gh_A12G2643 | 2.5621 | AT1G18870 | isochorismate synthase 2 |
| Gh_A13G0175 | -3.134 | AT5G49690 | UDP-Glycosyltransferase superfamily protein |
| Gh_A13G0183 | -3.0315 | AT4G17810 | C2H2 and C2HC zinc fingers superfamily protein |
| Gh_A13G0249 | -2.7081 | AT1G68040 | S-adenosyl-L-methionine-dependent methyltransferases superfamily protein |
| Gh_A13G0256 | 2.1589 | AT5G37490 | ARM repeat superfamily protein |
| Gh_A13G0411 | 2.275 | AT5G48490 | Bifunctional inhibitor/lipid-transfer protein/seed storage 2S albumin superfamily protein |
| Gh_A13G0713 | 2.0587 | AT3G29320 | Glycosyl transferase, family 35 |
| Gh_A13G0783 | -2.4303 | AT2G26710 | Cytochrome P450 superfamily protein |
| Gh_A13G0844 | -2.5439 | AT1G06620 | 2-oxoglutarate (2OG) and Fe(II)-dependent oxygenase superfamily protein |
| Gh_A13G0935 | -2.1123 | AT1G56600 | galactinol synthase 2 |
| Gh_A13G1025 | -2.7636 | AT5G06900 | cytochrome P450, family 93, subfamily D, polypeptide 1 |
| Gh_A13G1043 | 2.1719 | AT4G05100 | myb domain protein 74 |

| Gene ID | log2 (GaJAZ1/WT) | Arabidopsis ID | Annotation (acc. To TAIR) |
|---|---|---|---|
| Gh_A13G1388 | -2.6434 | AT3G60470 | Plant protein of unknown function |
| Gh_A13G1611 | -2.0011 | AT2G18660 | plant natriuretic peptide A |
| Gh_A13G1634 | -3.4495 | AT4G32650 | potassium channel in Arabidopsis thaliana 3 |
| Gh_A13G1852 | -2.2624 | AT5G19640 | Major facilitator superfamily protein |
| Gh_A13G2059 | 2.0697 | AT2G40610 | expansin A8 |
| Gh_A13G2173 | 4.7186 | AT3G53990 | Adenine nucleotide alpha hydrolases-like superfamily protein |
| Gh_A13G2193 | -2.2077 | AT5G06060 | NAD(P)-binding Rossmann-fold superfamily protein |
| Gh_A13G2339 | -2.1764 | AT1G52820 | 2-oxoglutarate (2OG) and Fe(II)-dependent oxygenase superfamily protein |
| Gh_D01G0156 | 4.2775 | AT2G20340 | Pyridoxal phosphate (PLP)-dependent transferases superfamily protein |
| Gh_D01G0168 | -2.2059 | AT4G03510 | RING membrane-anchor 1 |
| Gh_D01G0169 | Inf | AT1G53025 | Ubiquitin-conjugating enzyme family protein |
| Gh_D01G0180 | -2.7109 | AT1G03790 | Zinc finger C-x8-C-x5-C-x3-H type family protein |
| Gh_D01G0253 | -2.0388 | AT1G11300 | protein serine/threonine kinases |
| Gh_D01G0263 | -2.2415 | AT1G11300 | protein serine/threonine kinases |
| Gh_D01G0280 | 2.6623 | AT3G12900 | 2-oxoglutarate (2OG) and Fe(II)-dependent oxygenase superfamily protein |
| Gh_D01G0517 | 2.5652 | AT5G54370 | Late embryogenesis abundant (LEA) protein-related |
| Gh_D01G0787 | -2.0437 | AT5G07280 | Leucine-rich repeat transmembrane protein kinase |
| Gh_D01G1015 | -2.0638 | AT2G26530 | Protein of unknown function |
| Gh_D01G1714 | -2.6442 | AT1G68150 | WRKY DNA-binding protein 9 |
| Gh_D01G1748 | 2.3991 | AT4G37850 | basic helix-loop-helix (bHLH) DNA-binding superfamily protein |
| Gh_D01G1760 | -2.2616 | AT4G13420 | high affinity K+ transporter 5 |
| Gh_D01G1763 | -2.1762 | AT4G13420 | high affinity K+ transporter 5 |
| Gh_D01G1908 | -2.4534 | AT5G06900 | cytochrome P450, family 93, subfamily D, polypeptide 1 |
| Gh_D01G1909 | -3.1659 | AT2G42250 | cytochrome P450, family 712, subfamily A, polypeptide 1 |
| Gh_D01G2145 | -2.3031 | AT3G06880 | Transducin/WD40 repeat-like superfamily protein |
| Gh_D02G0598 | 2.2081 | AT1G60360 | RING/U-box superfamily protein |

| Gene ID | log2 (GaJAZ1/WT) | Arabidopsis ID | Annotation (acc. To TAIR) |
|---|---|---|---|
| Gh_D02G0712 | -3.675 | AT5G45340 | cytochrome P450, family 707, subfamily A, polypeptide 3 |
| Gh_D02G0783 | -2.2705 | AT1G77330 | 2-oxoglutarate (2OG) and Fe(II)-dependent oxygenase superfamily protein |
| Gh_D02G1236 | -2.5238 | AT4G31330 | Protein of unknown function |
| Gh_D02G1405 | -2.2174 | AT1G31050 | basic helix-loop-helix (bHLH) DNA-binding superfamily protein |
| Gh_D02G1664 | -2.1441 | AT1G10560 | plant U-box 18 |
| Gh_D02G1728 | Inf | AT1G68510 | LOB domain-containing protein 42 |
| Gh_D02G1798 | -4.0484 | AT1G03840 | C2H2 and C2HC zinc fingers superfamily protein |
| Gh_D02G1848 | -2.7304 | AT4G35160 | O-methyltransferase family protein |
| Gh_D02G1872 | 3.6258 | AT4G10265 | Wound-responsive family protein |
| Gh_D02G1873 | 3.3079 | AT4G10265 | Wound-responsive family protein |
| Gh_D02G1998 | 2.0137 | AT5G64120 | Peroxidase superfamily protein |
| Gh_D02G2374 | -2.3212 | AT2G35060 | K+ uptake permease 11 |
| Gh_D02G2446 | -2.3909 | AT1G30330 | auxin response factor 6 |
| Gh_D03G0206 | -2.0009 | AT4G31500 | cytochrome P450, family 83, subfamily B, polypeptide 1 |
| Gh_D03G0239 | 2.3621 | AT2G14900 | Gibberellin-regulated family protein |
| Gh_D03G0747 | -2.7352 | AT5G53110 | RING/U-box superfamily protein |
| Gh_D03G0754 | -2.1619 | AT5G08590 | SNF1-related protein kinase 2.1 |
| Gh_D03G1207 | -2.4473 | AT1G07620 | GTP-binding protein Obg/CgtA |
| Gh_D03G1484 | -2.3988 | AT4G23030 | MATE efflux family protein |
| Gh_D03G1533 | 4.6961 | AT1G62790 | Bifunctional inhibitor/lipid-transfer protein/seed storage 2S albumin superfamily protein |
| Gh_D03G1568 | -2.7336 | AT5G47450 | tonoplast intrinsic protein 2;3 |
| Gh_D03G1604 | -2.4419 | AT1G61590 | Protein kinase superfamily protein |
| Gh_D03G1706 | -2.2533 | AT1G52800 | 2-oxoglutarate (2OG) and Fe(II)-dependent oxygenase superfamily protein |
| Gh_D03G1794 | -2.2656 | AT1G52820 | 2-oxoglutarate (2OG) and Fe(II)-dependent oxygenase superfamily protein |
| Gh_D04G0262 | 2.8011 | AT1G28130 | Auxin-responsive GH3 family protein |
| Gh_D04G0470 | 2.2097 | AT5G15130 | WRKY DNA-binding protein 72 |

| Gene ID | log2 (GaJAZ1/WT) | Arabidopsis ID | Annotation (acc. To TAIR) |
|---|---|---|---|
| Gh_D04G0732 | -2.3664 | AT5G40960 | Protein of unknown function |
| Gh_D04G0816 | -2.3263 | AT1G11300 | protein serine/threonine kinases |
| Gh_D04G1016 | -2.0636 | AT3G52970 | cytochrome P450, family 76, subfamily G, polypeptide 1 |
| Gh_D04G1303 | -2.2477 | AT2G01570 | GRAS family transcription factor family protein |
| Gh_D04G1304 | -2.8865 | | Nodulation-signaling pathway 2 protein |
| Gh_D04G1434 | -2.147 | AT4G28110 | myb domain protein 41 |
| Gh_D04G1494 | -2.3841 | AT3G43660 | Vacuolar iron transporter (VIT) family protein |
| Gh_D04G1548 | -2.7928 | AT5G49040 | Disease resistance-responsive (dirigent-like protein) family protein |
| Gh_D04G1853 | -3.7419 | AT4G10310 | high-affinity K+ transporter 1 |
| Gh_D05G0054 | -2.2809 | AT3G46530 | NB-ARC domain-containing disease resistance protein |
| Gh_D05G0541 | -2.0048 | AT5G48930 | hydroxycinnamoyl-CoA shikimate/quinate hydroxycinnamoyl transferase |
| Gh_D05G0858 | -2.2028 | AT2G28160 | FER-like regulator of iron uptake |
| Gh_D05G0956 | -3.2455 | AT2G36830 | gamma tonoplast intrinsic protein |
| Gh_D05G1022 | -2.1879 | AT2G38300 | myb-like HTH transcriptional regulator family protein |
| Gh_D05G1036 | -2.2869 | AT3G51680 | NAD(P)-binding Rossmann-fold superfamily protein |
| Gh_D05G1137 | 2.5328 | AT1G80840 | WRKY DNA-binding protein 40 |
| Gh_D05G1582 | 2.8212 | AT4G28240 | Wound-responsive family protein |
| Gh_D05G1642 | 5.4778 | AT4G22030 | F-box family protein with a domain of unknown function |
| Gh_D05G1754 | 2.6457 | AT1G20190 | expansin 11 |
| Gh_D05G1780 | -2.4309 | AT1G75750 | GAST1 protein homolog 1 |
| Gh_D05G1896 | -2.0727 | AT4G31940 | cytochrome P450, family 82, subfamily C, polypeptide 4 |
| Gh_D05G1897 | -3.6758 | AT4G31940 | cytochrome P450, family 82, subfamily C, polypeptide 4 |
| Gh_D05G1973 | 2.0238 | AT4G34150 | Calcium-dependent lipid-binding (CaLB domain) family protein |
| Gh_D05G1997 | 3.9908 | AT2G14900 | Gibberellin-regulated family protein |
| Gh_D05G2218 | -4.1551 | AT4G08250 | GRAS family transcription factor |
| Gh_D05G2433 | Inf | AT1G20190 | expansin 11 |

| Gene ID | log2 (GaJAZ1/WT) | Arabidopsis ID | Annotation (acc. To TAIR) |
|---|---|---|---|
| Gh_D05G2505 | 2.4411 | AT5G59845 | Gibberellin-regulated family protein |
| Gh_D05G2912 | -2.2868 | AT2G38080 | Laccase/Diphenol oxidase family protein |
| Gh_D05G2949 | -2.379 | AT1G18650 | plasmodesmata callose-binding protein 3 |
| Gh_D05G3827 | -2.0162 | AT1G29280 | WRKY DNA-binding protein 65 |
| Gh_D06G0152 | -3.5577 | AT3G50330 | basic helix-loop-helix (bHLH) DNA-binding superfamily protein |
| Gh_D06G0177 | 2.0503 | AT4G34150 | Calcium-dependent lipid-binding (CaLB domain) family protein |
| Gh_D06G0183 | -3.344 | AT3G53010 | Domain of unknown function |
| Gh_D06G0388 | -3.2249 | AT4G08250 | GRAS family transcription factor |
| Gh_D06G0637 | -2.3914 | AT4G28890 | RING/U-box superfamily protein |
| Gh_D06G0842 | -2.2531 | AT2G26290 | root-specific kinase 1 |
| Gh_D06G1673 | -2.2825 | AT4G03965 | RING/U-box superfamily protein |
| Gh_D06G2051 | -2.1872 | AT1G17620 | Late embryogenesis abundant (LEA) hydroxyproline-rich glycoprotein family |
| Gh_D06G2071 | -2.0807 | AT5G48930 | hydroxycinnamoyl-CoA shikimate/quinate hydroxycinnamoyl transferase |
| Gh_D06G2077 | -2.7209 | AT5G48930 | hydroxycinnamoyl-CoA shikimate/quinate hydroxycinnamoyl transferase |
| Gh_D06G2078 | -2.0549 | AT5G48930 | hydroxycinnamoyl-CoA shikimate/quinate hydroxycinnamoyl transferase |
| Gh_D06G2222 | -2.8934 | AT1G12030 | Protein of unknown function |
| Gh_D07G0079 | -2.1237 | AT2G20030 | RING/U-box superfamily protein |
| Gh_D07G0265 | -2.13 | AT4G30960 | SOS3-interacting protein 3 |
| Gh_D07G0385 | -2.2939 | AT1G15550 | gibberellin 3-oxidase 1 |
| Gh_D07G0404 | -3.3623 | AT5G51800 | Protein kinase superfamily protein |
| Gh_D07G0781 | -3.3427 | AT3G30210 | myb domain protein 121 |
| Gh_D07G1078 | -2.0493 | AT1G08320 | bZIP transcription factor family protein |
| Gh_D07G1169 | 2.7072 | AT5G60520 | Late embryogenesis abundant (LEA) protein-related |
| Gh_D07G1527 | 2.2872 | AT5G44700 | Leucine-rich repeat transmembrane protein kinase |
| Gh_D07G1641 | 3.0099 | AT5G03795 | Exostosin family protein |
| Gh_D07G1705 | -2.41 | AT1G66140 | zinc finger protein 4 |

| Gene ID | log2 (GaJAZ1/WT) | Arabidopsis ID | Annotation (acc. To TAIR) |
|---|---|---|---|
| Gh_D07G1809 | 2.4975 | AT5G15780 | Pollen Ole e 1 allergen and extensin family protein |
| Gh_D07G1898 | -2.4363 | AT3G14630 | cytochrome P450, family 72, subfamily A, polypeptide 9 |
| Gh_D07G1902 | -2.9714 | AT5G14180 | Myzus persicae-induced lipase 1 |
| Gh_D07G1920 | -8.0056 | AT3G07810 | RNA-binding (RRM/RBD/RNP motifs) family protein |
| Gh_D07G1930 | 4.2189 | AT3G63380 | ATPase E1-E2 type family protein / haloacid dehalogenase-like hydrolase family protein |
| Gh_D07G1947 | -2.207 | AT4G39950 | cytochrome P450, family 79, subfamily B, polypeptide 2 |
| Gh_D07G2208 | -2.2829 | AT2G36910 | ATP binding cassette subfamily B1 |
| Gh_D07G2239 | -2.0525 | AT1G23740 | Oxidoreductase, zinc-binding dehydrogenase family protein |
| Gh_D07G2291 | -3.3757 | AT5G14340 | myb domain protein 40 |
| Gh_D07G2498 | -3.0089 | AT3G23240 | ethylene response factor 1 |
| Gh_D08G0007 | -2.3199 | AT2G37170 | plasma membrane intrinsic protein 2 |
| Gh_D08G0103 | -2.0352 | AT2G27370 | Uncharacterised protein family |
| Gh_D08G0238 | -2.2791 | AT2G18960 | H(+)-ATPase 1 |
| Gh_D08G0442 | 4.0783 | AT1G05260 | Peroxidase superfamily protein |
| Gh_D08G0443 | Inf | AT1G05260 | Peroxidase superfamily protein |
| Gh_D08G0451 | -2.1501 | AT1G02400 | gibberellin 2-oxidase 6 |
| Gh_D08G0487 | -2.0187 | AT5G18840 | Major facilitator superfamily protein |
| Gh_D08G0629 | 2.3523 | AT2G44840 | ethylene-responsive element binding factor 13 |
| Gh_D08G0650 | -3.1873 | AT2G42440 | Lateral organ boundaries (LOB) domain family protein |
| Gh_D08G0925 | -2.3182 | AT1G11300 | protein serine/threonine kinases |
| Gh_D08G1222 | -2.6261 | AT5G53110 | RING/U-box superfamily protein |
| Gh_D08G1534 | 4.1949 | AT2G44840 | ethylene-responsive element binding factor 13 |
| Gh_D08G1535 | 4.1623 | AT2G44840 | ethylene-responsive element binding factor 13 |
| Gh_D08G1696 | -2.3895 | AT4G17785 | myb domain protein 39 |
| Gh_D08G1697 | -2.3251 | AT5G16770 | myb domain protein 9 |
| Gh_D08G1799 | 5.4095 | AT4G17500 | ethylene responsive element binding factor 1 |

| Gene ID | log2 (GaJAZ1/WT) | Arabidopsis ID | Annotation (acc. To TAIR) |
|---|---|---|---|
| Gh_D08G1879 | -3.115 | AT4G12110 | sterol-4alpha-methyl oxidase 1-1 |
| Gh_D08G1950 | -2.142 | AT1G63440 | heavy metal atpase 5 |
| Gh_D08G2042 | -2.2891 | AT5G24530 | 2-oxoglutarate (2OG) and Fe(II)-dependent oxygenase superfamily protein |
| Gh_D08G2048 | -2.6246 | AT2G31160 | Protein of unknown function |
| Gh_D08G2330 | 3.0716 | AT5G17820 | Peroxidase superfamily protein |
| Gh_D08G2656 | -2.4765 | AT2G42610 | Protein of unknown function |
| Gh_D09G0151 | -3.5098 | AT1G26870 | NAC (No Apical Meristem) domain transcriptional regulator superfamily protein |
| Gh_D09G0567 | -2.7184 | AT3G54700 | phosphate transporter 1;7 |
| Gh_D09G0856 | -2.9325 | AT5G22890 | C2H2 and C2HC zinc fingers superfamily protein |
| Gh_D09G0859 | -2.4953 | AT2G28160 | FER-like regulator of iron uptake |
| Gh_D09G0928 | -2.5101 | AT1G08320 | bZIP transcription factor family protein |
| Gh_D09G1019 | 2.0635 | AT5G60520 | Late embryogenesis abundant (LEA) protein-related |
| Gh_D09G1261 | 2.3139 | AT2G35930 | plant U-box 23 |
| Gh_D09G1579 | -2.3294 | AT5G66770 | GRAS family transcription factor |
| Gh_D09G1631 | 2.3016 | AT3G17730 | NAC domain containing protein 57 |
| Gh_D09G1714 | 2.7474 | AT5G52400 | cytochrome P450, family 715, subfamily A, polypeptide 1 |
| Gh_D09G1743 | -3.2574 | AT5G59220 | highly ABA-induced PP2C gene 1 |
| Gh_D09G1969 | -2.1035 | AT5G07990 | Cytochrome P450 superfamily protein |
| Gh_D09G1999 | -2.2571 | AT1G06000 | UDP-Glycosyltransferase superfamily protein |
| Gh_D09G2000 | -3.1718 | AT1G06000 | UDP-Glycosyltransferase superfamily protein |
| Gh_D10G0061 | -2.6617 | AT1G30650 | WRKY DNA-binding protein 14 |
| Gh_D10G0086 | -2.1599 | AT5G47560 | tonoplast dicarboxylate transporter |
| Gh_D10G0452 | -2.0576 | AT1G31320 | LOB domain-containing protein 4 |
| Gh_D10G0689 | -2.5424 | AT1G52820 | 2-oxoglutarate (2OG) and Fe(II)-dependent oxygenase superfamily protein |
| Gh_D10G1381 | -3.2589 | AT2G15080 | receptor like protein 19 |
| Gh_D10G1462 | -2.5221 | AT1G11300 | protein serine/threonine kinases |

| Gene ID | log2 (GaJAZ1/WT) | Arabidopsis ID | Annotation (acc. To TAIR) |
|---|---|---|---|
| Gh_D10G1706 | -2.6234 | AT3G16660 | Pollen Ole e 1 allergen and extensin family protein |
| Gh_D10G1784 | 2.4402 | AT3G17070 | Peroxidase family protein |
| Gh_D10G1865 | 3.1968 | AT5G05260 | cytochrome p450 79a2 |
| Gh_D10G2000 | -3.6013 | AT4G31500 | cytochrome P450, family 83, subfamily B, polypeptide 1 |
| Gh_D10G2050 | -2.1062 | AT5G24910 | cytochrome P450, family 714, subfamily A, polypeptide 1 |
| Gh_D10G2235 | -3.2976 | AT1G74170 | receptor like protein 13 |
| Gh_D10G2258 | -4.0747 | AT4G31500 | cytochrome P450, family 83, subfamily B, polypeptide 1 |
| Gh_D10G2359 | -2.4141 | AT4G00430 | plasma membrane intrinsic protein 1;4 |
| Gh_D10G2360 | -2.1178 | AT4G00430 | plasma membrane intrinsic protein 1;4 |
| Gh_D10G2380 | -3.973 | AT4G20140 | Leucine-rich repeat transmembrane protein kinase |
| Gh_D11G0124 | -2.0777 | AT5G24030 | SLAC1 homologue 3 |
| Gh_D11G0275 | 2.8058 | AT3G10600 | cationic amino acid transporter 7 |
| Gh_D11G0316 | -2.0956 | AT2G47140 | NAD(P)-binding Rossmann-fold superfamily protein |
| Gh_D11G0439 | -3.1213 | AT3G02880 | Leucine-rich repeat protein kinase family protein |
| Gh_D11G0479 | -2.5899 | AT2G33580 | Protein kinase superfamily protein |
| Gh_D11G0495 | -2.2205 | AT4G37050 | PATATIN-like protein 4 |
| Gh_D11G0704 | -2.6645 | AT5G63090 | Lateral organ boundaries (LOB) domain family protein |
| Gh_D11G0743 | -2.001 | AT5G61430 | NAC domain containing protein 100 |
| Gh_D11G0746 | -2.2832 | AT3G01090 | SNF1 kinase homolog 10 |
| Gh_D11G0802 | 2.1581 | AT2G44840 | ethylene-responsive element binding factor 13 |
| Gh_D11G0871 | -2.3442 | AT2G45510 | cytochrome P450, family 704, subfamily A, polypeptide 2 |
| Gh_D11G1188 | -2.1831 | AT1G02810 | Plant invertase/pectin methylesterase inhibitor superfamily |
| Gh_D11G1339 | -2.9414 | AT4G17810 | C2H2 and C2HC zinc fingers superfamily protein |
| Gh_D11G1353 | -2.555 | AT4G10500 | 2-oxoglutarate (2OG) and Fe(II)-dependent oxygenase superfamily protein |
| Gh_D11G1542 | -2.4411 | AT3G07870 | F-box and associated interaction domains-containing protein |
| Gh_D11G1989 | -2.3114 | AT2G14960 | Auxin-responsive GH3 family protein |

| Gene ID | log2 (GaJAZ1/WT) | Arabidopsis ID | Annotation (acc. To TAIR) |
|---|---|---|---|
| Gh_D11G2073 | -2.3082 | AT4G37850 | basic helix-loop-helix (bHLH) DNA-binding superfamily protein |
| Gh_D11G2781 | -2.2865 | AT5G02070 | Protein kinase family protein |
| Gh_D11G2926 | 4.956 | AT4G38700 | Disease resistance-responsive (dirigent-like protein) family protein |
| Gh_D11G2943 | -2.1175 | AT1G28130 | Auxin-responsive GH3 family protein |
| Gh_D11G3081 | -2.2958 | AT3G56970 | basic helix-loop-helix (bHLH) DNA-binding superfamily protein |
| Gh_D11G3107 | -2.0258 | AT4G27220 | NB-ARC domain-containing disease resistance protein |
| Gh_D11G3156 | -2.052 | AT3G14470 | NB-ARC domain-containing disease resistance protein |
| Gh_D11G3200 | -2.7544 | AT3G14470 | NB-ARC domain-containing disease resistance protein |
| Gh_D11G3217 | -2.1908 | AT1G52340 | NAD(P)-binding Rossmann-fold superfamily protein |
| Gh_D11G3255 | -5.2797 | AT3G52970 | cytochrome P450, family 76, subfamily G, polypeptide 1 |
| Gh_D11G3268 | -2.3296 | AT2G36830 | gamma tonoplast intrinsic protein |
| Gh_D11G3390 | -2.3306 | AT3G14470 | NB-ARC domain-containing disease resistance protein |
| Gh_D11G3457 | -3.208 | AT5G67150 | HXXXD-type acyl-transferase family protein |
| Gh_D12G0121 | -2.0056 | AT2G17290 | Calcium-dependent protein kinase family protein |
| Gh_D12G0234 | -2.8666 | AT2G23610 | methyl esterase 3 |
| Gh_D12G0346 | 2.8462 | AT1G20450 | Dehydrin family protein |
| Gh_D12G0567 | 2.5974 | AT1G75750 | GAST1 protein homolog 1 |
| Gh_D12G0671 | Inf | AT5G42940 | RING/U-box superfamily protein |
| Gh_D12G0699 | 2.0894 | AT4G37530 | Peroxidase superfamily protein |
| Gh_D12G0899 | -2.071 | AT2G21990 | Protein of unknown function |
| Gh_D12G0918 | 2.9732 | AT5G10130 | Pollen Ole e 1 allergen and extensin family protein |
| Gh_D12G0958 | 3.2389 | AT2G44840 | ethylene-responsive element binding factor 13 |
| Gh_D12G0959 | 3.2313 | AT2G44840 | ethylene-responsive element binding factor 13 |
| Gh_D12G1152 | -3.1672 | AT2G46680 | homeobox 7 |
| Gh_D12G1632 | -2.2357 | AT5G15290 | Uncharacterised protein family |
| Gh_D12G1793 | -2.2656 | AT3G02100 | UDP-Glycosyltransferase superfamily protein |

| Gene ID | log2 (GaJAZ1/WT) | Arabidopsis ID | Annotation (acc. To TAIR) |
|---|---|---|---|
| Gh_D12G1840 | -2.5758 | AT3G26310 | cytochrome P450, family 71, subfamily B, polypeptide 35 |
| Gh_D12G2243 | -2.0659 | AT4G11655 | Uncharacterised protein family |
| Gh_D12G2370 | -2.0279 | AT1G33030 | O-methyltransferase family protein |
| Gh_D12G2561 | -2.0757 | AT3G24020 | Disease resistance-responsive (dirigent-like protein) family protein |
| Gh_D12G2576 | 4.336 | AT5G37490 | ARM repeat superfamily protein |
| Gh_D12G2605 | 2.4121 | AT5G14740 | carbonic anhydrase 2 |
| Gh_D13G0266 | -2.403 | AT1G68040 | S-adenosyl-L-methionine-dependent methyltransferases superfamily protein |
| Gh_D13G0791 | -2.9487 | | rRNA N-glycosidase (Fragment) |
| Gh_D13G0883 | -3.7541 | AT3G51550 | Malectin/receptor-like protein kinase family protein |
| Gh_D13G0962 | -3.1692 | AT2G21210 | SAUR-like auxin-responsive protein family |
| Gh_D13G0971 | -2.1694 | AT5G06060 | NAD(P)-binding Rossmann-fold superfamily protein |
| Gh_D13G0980 | 2.0244 | AT3G53990 | Adenine nucleotide alpha hydrolases-like superfamily protein |
| Gh_D13G1271 | -2.2182 | AT1G71930 | vascular related NAC-domain protein 7 |
| Gh_D13G1294 | 3.1484 | AT4G21440 | MYB-like 102 |
| Gh_D13G1655 | -3.4943 | AT3G13610 | 2-oxoglutarate (2OG) and Fe(II)-dependent oxygenase superfamily protein |
| Gh_D13G1900 | -2.2548 | AT1G09380 | nodulin MtN21 /EamA-like transporter family protein |
| Gh_D13G2317 | -2.0965 | AT5G22220 | E2F transcription factor 1 |
| Gh_D13G2460 | 2.0773 | AT2G40610 | expansin A8 |
| Gh_D13G2465 | -2.0002 | AT1G52820 | 2-oxoglutarate (2OG) and Fe(II)-dependent oxygenase superfamily protein |
| Gh_Sca005458G01 | 4.0805 | AT3G48270 | cytochrome P450, family 71, subfamily A, polypeptide 26 |
| Gh_Sca005510G01 | -2.7631 | AT2G26710 | Cytochrome P450 superfamily protein |
| Gh_Sca007264G01 | -2.6294 | AT1G09155 | phloem protein 2-B15 |
| Gh_Sca010862G01 | -2.3571 | AT4G21700 | Protein of unknown function |
| Novel00107 | -2.3809 | -- | hypothetical protein F383_20901 |
| Novel00838 | -3.1975 | -- | PREDICTED: cytochrome P450 CYP749A22-like |
| Novel00877 | -2.1451 | -- | Cytochrome P450 protein |

| Gene ID | log2 (GaJAZ1/WT) | Arabidopsis ID | Annotation (acc. To TAIR) |
|---|---|---|---|
| Novel01012 | -3.6931 | -- | PREDICTED: putative receptor-like protein kinase At4g00960 isoform X2 |
| Novel01626 | 5.8859 | -- | PREDICTED: auxin-responsive protein IAA29 |
| Novel01663 | -2.1177 | -- | auxin response factor, partial |
| Novel02264 | -2.8278 | -- | putative WRKY transcription factor 16 -like protein |
| Novel02986 | 4.499 | -- | Homeobox prospero |
| Novel03310 | -2.8644 | -- | PREDICTED: LRR receptor-like serine/threonine-protein kinase EFR |
| Novel03492 | -2.4159 | -- | PREDICTED: cytochrome P450 CYP749A22-like |
| Novel03493 | -2.7515 | -- | PREDICTED: cytochrome P450 CYP749A22-like |
| Novel03664 | 6.7947 | -- | PREDICTED: F-box/kelch-repeat protein At5g60570-like isoform X1 |
| Novel03971 | -2.316 | -- | Cytochrome P450 protein |
| Novel05745 | -2.1509 | -- | PREDICTED: cytochrome P450 CYP749A22-like |
| Novel05750 | -5.8342 | -- | PREDICTED: auxin-induced protein 15A-like |
| Novel06076 | -2.8456 | -- | Gibberellin 20 oxidase 1 -like protein |
| Novel06255 | -3.1009 | -- | PREDICTED: putative RING-H2 finger protein ATL21B |
| Novel06283 | -3.3245 | -- | NADH-ubiquinone oxidoreductase chain 4L |
| Novel06462 | -2.8766 | -- | PREDICTED: receptor-like protein 12 |
